# Sea cucumber genome provides insights into saponin biosynthesis and aestivation regulation

**DOI:** 10.1038/s41421-018-0030-5

**Published:** 2018-06-26

**Authors:** Yuli Li, Ruijia Wang, Xiaogang Xun, Jing Wang, Lisui Bao, Ramesha Thimmappa, Jun Ding, Jingwei Jiang, Liheng Zhang, Tianqi Li, Jia Lv, Chuang Mu, Xiaoli Hu, Lingling Zhang, Jing Liu, Yuqiang Li, Lijie Yao, Wenqian Jiao, Yangfan Wang, Shanshan Lian, Zelong Zhao, Yaoyao Zhan, Xiaoting Huang, Huan Liao, Jia Wang, Hongzhen Sun, Xue Mi, Yu Xia, Qiang Xing, Wei Lu, Anne Osbourn, Zunchun Zhou, Yaqing Chang, Zhenmin Bao, Shi Wang

**Affiliations:** 1 MOE Key Laboratory of Marine Genetics and Breeding, Ocean University of China, Qingdao, 266003 China; 2 Laboratory for Marine Biology and Biotechnology, Qingdao National Laboratory for Marine Science and Technology, Qingdao, 266237 China; 3 The Ben May Department for Cancer Research, The University of Chicago, Chicago, IL 60637 USA; 4 Department of Metabolic Biology, John Innes Centre, Norwich Research Park, Norwich, NR4 7UH United Kingdom; 5 College of Fisheries and Life Science, Dalian Ocean University, Dalian, 116023 China; 6 Liaoning Key Lab of Marine Fishery Molecular Biology, Liaoning Ocean and Fisheries Science Research Institute, Dalian, 116023 China; 7 Laboratory for Marine Fisheries Science and Food Production Processes, Qingdao National Laboratory for Marine Science and Technology, Qingdao, 266237 China

## Abstract

Echinoderms exhibit several fascinating evolutionary innovations that are rarely seen in the animal kingdom, but how these animals attained such features is not well understood. Here we report the sequencing and analysis of the genome and extensive transcriptomes of the sea cucumber *Apostichopus japonicus*, a species from a special echinoderm group with extraordinary potential for saponin synthesis, aestivation and organ regeneration. The sea cucumber does not possess a reorganized *Hox* cluster as previously assumed for all echinoderms, and the spatial expression of *Hox7* and *Hox11/13b* potentially guides the embryo-to-larva axial transformation. Contrary to the typical production of lanosterol in animal cholesterol synthesis, the oxidosqualene cyclase of sea cucumber produces parkeol for saponin synthesis and has “plant-like” motifs suggestive of convergent evolution. The transcriptional factors *Klf2* and *Egr1* are identified as key regulators of aestivation, probably exerting their effects through a clock gene-controlled process. Intestinal hypometabolism during aestivation is driven by the DNA hypermethylation of various metabolic gene pathways, whereas the transcriptional network of intestine regeneration involves diverse signaling pathways, including Wnt, Hippo and FGF. Decoding the sea cucumber genome provides a new avenue for an in-depth understanding of the extraordinary features of sea cucumbers and other echinoderms.

## Introduction

Echinoderms, which first appeared in the early Cambrian period^1^, represent the second largest group of deuterostomes, and together with their sister phylum Hemichordata, they occupy a critical phylogenetic position for understanding the evolutionary origin of chordates^2^. The radiation of echinoderms was believed to be responsible for the Mesozoic Marine Revolution^3^, and they can adapt to various oceanic environments, even to the biotic desert of the deep sea. Their superb adaptation attributes include several fascinating evolutionary innovations that are rarely seen in the animal kingdom such as a pentaradial body plan with rigid calcitic skeletons^4^, biosynthesis of saponins^5^, and extraordinary potential for regeneration^6^. However, our understanding of their biology and evolution is hindered by poor sampling of their genomes^7–10^, and their relatively large, highly heterozygous genomes generally challenge our ability to obtain high-quality assemblies^8,11,12^.

Sea cucumbers (Supplementary Figure S1), praised as the “jewel of the seabed”^13^, are a special echinoderm group that possesses the well-known striking features of echinoderms and are more distinctive than other echinoderm groups in aspects of saponin biosynthesis, aestivation and visceral regeneration. Sea cucumbers can produce saponins (also known as holothurins), which act as chemical defense agents against predators and parasites^14,15^ and are also highly valued bioactive compounds with many appealing biomedical properties, such as anti-cancer, anti-inflammatory, anti-bacterial and immunomodulatory effects (sea cucumbers are sometimes referred to as “Marine Ginseng” in Asian countries)^16–18^. Saponins are widespread in plants but are rarely found in the animal kingdom^19,20^, and how sea cucumbers gained the ability to synthesize saponins remains enigmatic. In response to high temperatures, sea cucumbers can protect themselves by entering a physiological state called aestivation, which is a process that can last for up to 4 months^21^ and is characterized by inactivity, feeding cessation, intestine degeneration and metabolic rate depression^22,23^. Sea cucumbers also possess another defensive mechanism called evisceration, with which they can expel internal organs (e.g., intestine and respiratory tree) out of their body when they get stressed, and the missing organs can be regenerated concurrently within 7 days^24,25^. Although the aestivation and regeneration properties of sea cucumbers have received increasing research attention^23^, the molecular regulatory mechanisms underlying these interesting phenomena remain obscure.

The sea cucumber *Apostichopus japonicus* (Selenka 1867), represents one of the most dominant and economically important species in the Western Pacific Ocean along the coasts of China, Japan, Korea and Russia, with considerable edible and medicinal value^26^. It is also among the best molecularly characterized sea cucumber species^27–30^, making it a good candidate for whole-genome sequencing. Recent sequencing efforts have been devoted to understanding its morphological evolution and visceral regeneration^10,12^, but their saponin synthesis and aestivation abilities remain poorly explored at whole genome levels. Here, we report sequencing of the genome and extensive transcriptomes of *A*. *japonicus*. Our analysis revealed novel genomic features and molecular changes that may contribute to the evolutionary innovations of sea cucumber or echinoderm-characteristic adaptive traits, providing insights into saponin synthesis and aestivation regulation.

## Results

### Genome sequencing, assembly and characterization

**Fig. 1:**
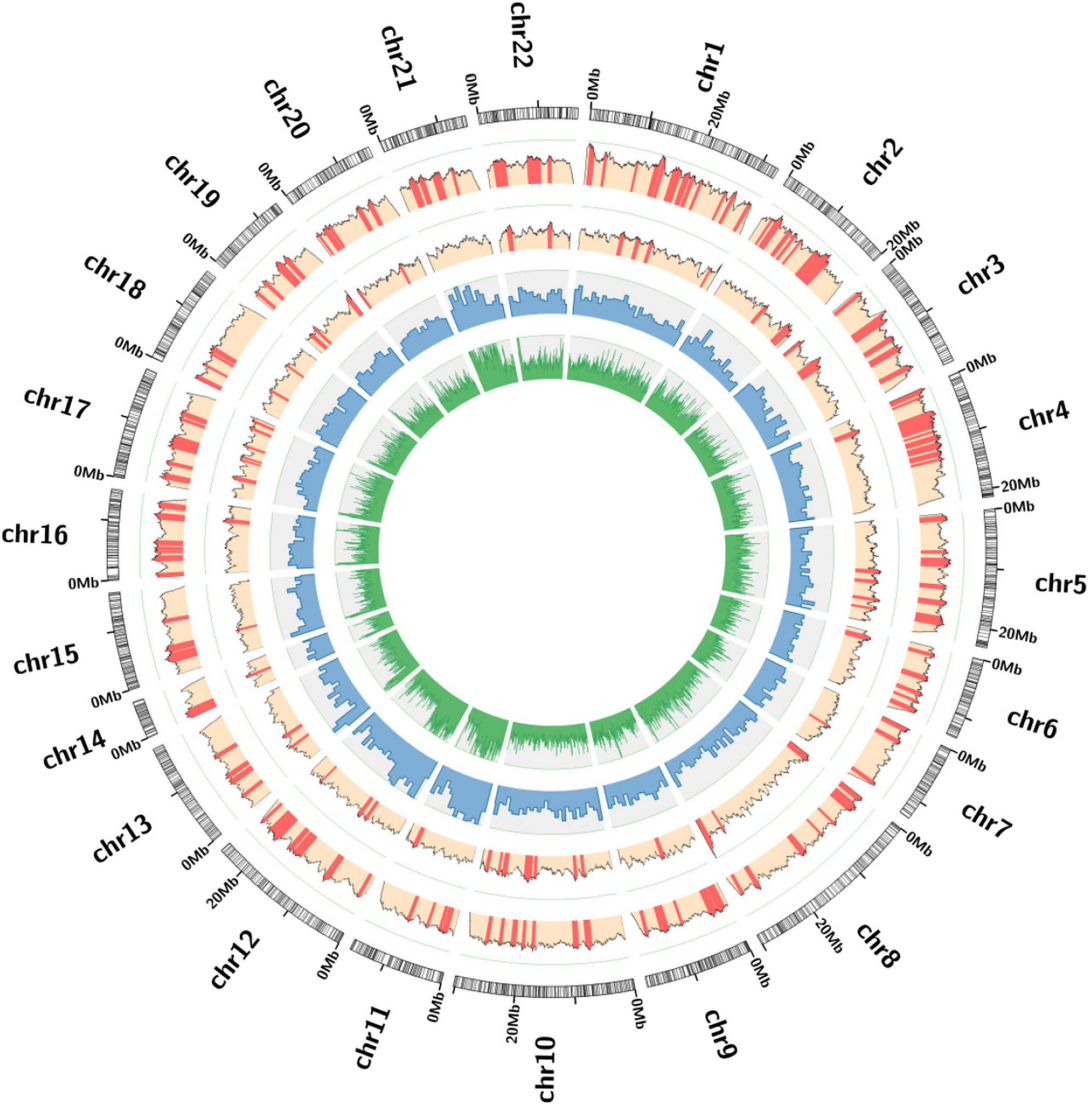
Genome landscape and polymorphism analysis of the sea cucumber *Apostichopus japonicus*. From outer to inner circles: I, marker distribution on 22 chromosomes at the Mb scale; II and III, single nucleotide polymorphism (SNP) density across genome (II) or CDS (III) drawn in 1-Mb sliding windows with a 50-kb step (yellow columns), and polymorphism hotspot regions (*p* *<* 1e–4) are colored red; IV and V, gene density and repeat density across the genome, respectively, drawn in 1-Mb non-overlapping windows

Like other marine invertebrates^31–34^, echinoderm genomes are generally difficult to sequence and assemble due to their relatively large genomes and/or remarkably high genome heterozygosity^8^. By taking advantage of both Illumina short-read and PacBio long-read sequencing technologies, we performed whole-genome shotgun sequencing of a wild individual of *A*. *japonicus*, which produced 352 Gb of clean data, corresponding to an average genome coverage of 370× (Supplementary Table S1). The final genome assembly was 952 Mb with a contig N50 of 45 kb and a scaffold N50 of 196 kb, and over 90% of the assembly was covered by the longest 4,784 scaffolds (>63 kb) (Supplementary Tables S2, S3). The total assembly length was close to the estimates from k-mer analysis (~1.0 Gb; Supplementary Figure S2) and flow cytometry analysis^35^ (~0.9 Gb; Supplementary Figure S3). The integrity of the assembly was demonstrated by mapping 97.4–99.3% of the transcriptome data sets (Supplementary Table S4). The assembly was further anchored to chromosomes based on a high-density genetic linkage map^28^ by assigning 1,949 scaffolds to 22 linkage groups (Fig. 1; Supplementary Table S5).

The sea cucumber genome contains 29,451 protein-coding genes supported by known protein sequences and/or transcriptomic data (Supplementary Table S6). Functional analysis via comparison with various public protein databases annotated all of the predicted genes (Supplementary Figure S4; Supplementary Tables S7, S8). The sea cucumber genome contains 254 Mb of repetitive sequences accounting for 26% of the genome. This percentage is lower than those of other echinoderm genomes (26-41%; Supplementary Table S9). DNA transposons represent the most abundant repeat type (3.5%), followed by long interspersed elements (2.1%) and tandem repeats (2.0%) (Supplementary Table S9; Supplementary Figure S5).

We conducted a comprehensive comparison of our assembly with the two *A*. *japonicus* assemblies generated in previous studies^10,12^. In terms of assembly quality, ours and Zhang’s assembly is much better than Jo’s version (Supplementary Table S10). Although Zhang’s assembly has higher contiguity than ours, gene completeness is comparable between the two assemblies (Supplementary Table S10), suggesting that the quality of both assemblies is sufficiently high for many downstream analyses. The majority of genes and pathways mentioned by Zhang et al.^10^ relating to morphological evolution and visceral regeneration could be found in our assembly with largely consistent biological and/or evolutionary characteristics (Supplementary Tables S11–S14).

Polymorphism analysis identified 2.43 million single-nucleotide polymorphisms (SNPs) in the assembled individual. A genome-wide scan of polymorphism based on the assembled individual and 8 additional resequenced individuals (Supplementary Table S15) identified 110 highly polymorphic genomic regions (≥500 kb) in the genome (Fig. 1). SNP density in coding sequences (CDSs) varies dramatically among genes, ranging from 0 to 222 SNPs per kb (Fig. 1). We identified 3,241 highly polymorphic genes (HPGs) that are statistically significant relative to the chromosomal background (Supplementary Table S16). Functional enrichment analysis of these HPGs reveals that they are involved in diverse cellular functions or biological processes such as catabolic processes, reproductive processes, regulation of homeostatic processes, and regulation of signaling (Supplementary Table S17). These HPGs may contribute to the sea cucumber’s superb adaptation by enhancing the plasticity of its coding repertoire.

### *Hox*/*ParaHox* clusters and body plan evolution

**Fig. 2:**
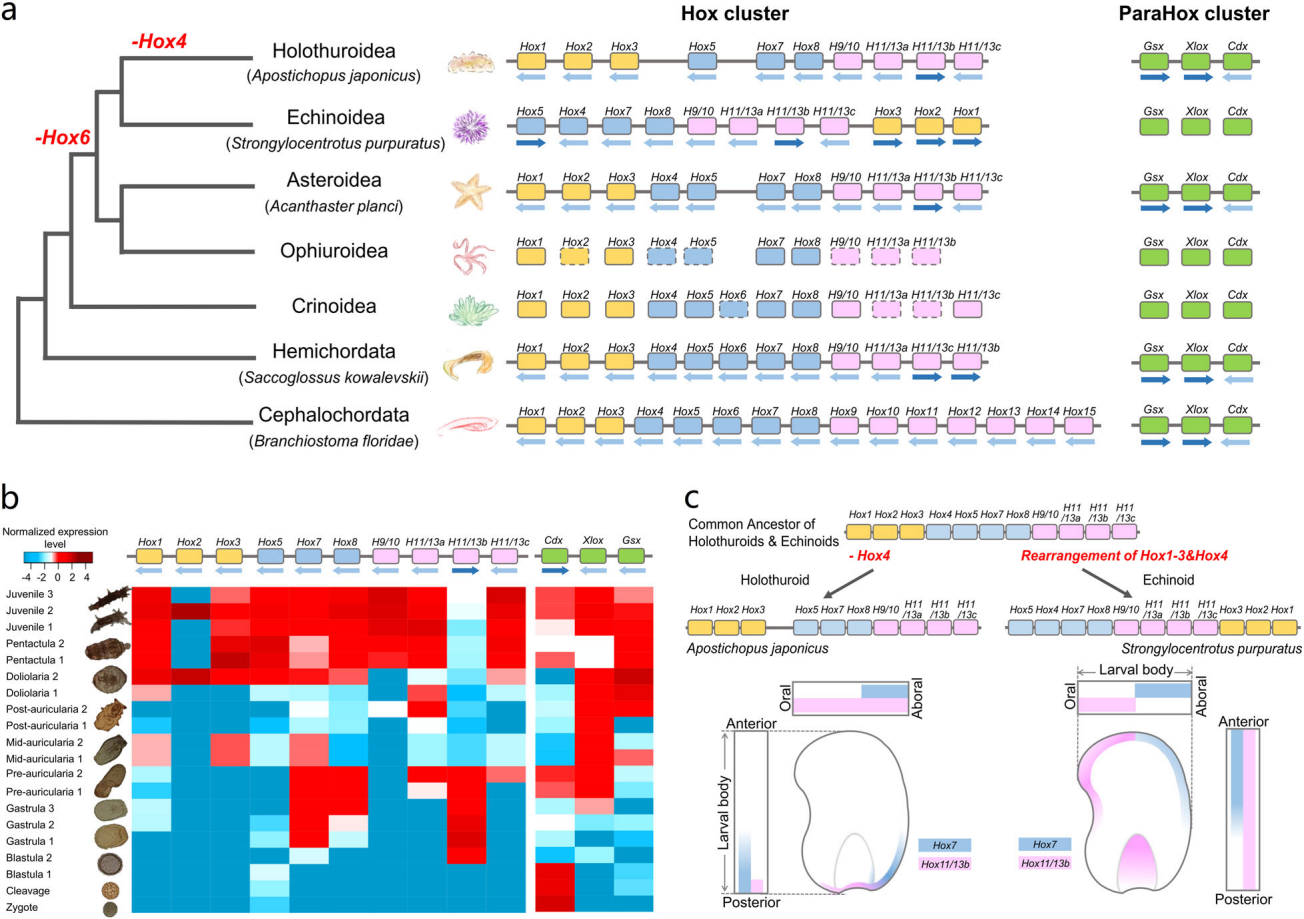
The cluster organization and tempo-spatial expression of *Hox* genes during the development of *A. japonicus*. **a** Cluster organization of the *Hox* and *ParaHox* genes of the sea cucumber *A*. *japonicus* and other echinoderms^37,41,112^. The sea cucumber has a typical *Hox* cluster similar to that of the sea star *A*. *planci*, invalidating the previous hypothesis that *Hox* clusters of all echinoderms are reorganized^39,40^. The genes whose identity existed ambiguously are shown with rectangles in dashed lines. **b** Temporal expression of the sea cucumber *Hox* and *ParaHox* cluster genes. Contrary to their *ParaHox* counterpart, expression of the sea cucumber *Hox* cluster during development does not exhibit temporal colinearity as typically found in chordate *Hox* clusters. Compared with other *Hox* genes, *Hox7* and *Hox11/13b* show prominent expression during gastrulation, likely participating in determination of the larval body plan. **c** Inferred Hox cluster evolution and spatial expression of *Hox7* and *Hox11/13b* in sea cucumber and sea urchins. Presumably the common ancestor of holothuroids and echinoids contained a typical *Hox* cluster without *Hox6*, which is largely preserved in the sea cucumber lineage (except the loss of *Hox4*) but had undergone a few rearrangements in the sea urchin lineage. The spatial expression of *Hox7* and *Hox11/13b* shows colinearity during gastrulationalong the anterior/posterior (A/P) axis for the sea cucumber *A*. *japonicus*^45^ and along the oral/aboral (O/A) axis for sea urchins^46–48^. This finding probably suggests the important roles of the two *Hox* genes in guiding axial transformation from the embryonic to larval stage in echinozoans

Echinoderms, albeit evolving from the same bilaterian ancestor that gave rise to chordates, display a variety of highly derived, Cambrian-derived body architectures^36^ for which the molecular driving force(s) remain poorly understood^37^. Understanding the organization of echinoderm *Hox* clusters is of particular interest, as *Hox* genes play a key role in animal body plan determination^38^. Much of our previous knowledge came from the sea urchin *Strongylocentrotus* *purpuratus*, leading to a prevailing hypothesis that the echinoderm pentameral body plan was a consequence of a reorganized *Hox* cluster^39,40^. This hypothesis was, however, recently challenged by the finding of an intact *Hox* cluster in the sea star *Acanthaster* *planci*^37,41^. Coinciding with the new report by Zhang et al.^10^, we identified a *Hox* cluster and a *ParaHox* cluster in the sea cucumber genome, and the gene composition and orientation of both clusters was highly consistent with those of the sea star *Hox* and *ParaHox* clusters (Fig. 2a; Supplementary Figure S6). The finding of typical *Hox* clusters in both sea cucumber and sea star suggests that it was unlikely that the ancestor of echinoderms possessed a reorganized *Hox* cluster as found in sea urchin, thus invalidating the previous hypothesis correlating the derived pentameral body plan with the reorganized *Hox* cluster^40^. Despite having a typical *Hox* cluster, the sea cucumber lacks *Hox4* and *Hox6*, and the latter is also absent in Echinoidea, Asteroidea and Ophiuroidea, implicating that the loss of *Hox6* might have already occurred before the split of Echinozoa and Asterozoa.

During development, most echinoderms rearrange their body plans through drastic axial transformation, e.g., from embryonic anterior/posterior (A/P) axis to larval/adult oral/aboral (O/A) axis, with changes from 0° in holothurians, 90° in echinoids to 180° in crinoids^42^. To gain insights into this poorly understood process, we investigated the expression dynamics of all *Hox* genes during sea cucumber embryonic and larval stages. We found that contrary to their *ParaHox* counterpart, the expression of the sea cucumber *Hox* cluster during development did not exhibit temporal colinearity as typically found in chordate *Hox* clusters^43^ (Fig. 2b), which is possibly related to the derived body plan. Compared with other *Hox* genes that are mostly expressed after metamorphosis, sea cucumber *Hox7* and *Hox11/13b* show prominent expression during gastrulation (Fig. 2b), and they are also the only two genes significantly expressed in sea urchin embryos^44^. Further investigation of the spatial expression of *Hox7* and *Hox11/13b* in sea cucumber^45^ and sea urchins^46–48^ revealed spatial colinearity during gastrulation but in different axial directions, i.e., A/P axis for sea cucumber and O/A axis for sea urchin (Fig. 2c), corresponding to their larval/adult axial directions (i.e., no axial change in sea cucumber, whereas there was a 90° change in sea urchin). This interesting finding suggests that *Hox7* and *Hox11/13b* might play an important role in guiding axial transformation from the embryonic to larval stage. Considering that axial transformation before larval metamorphosis only occurs in the sea urchin lineage, it is likely that such a unique transformation in sea urchin could be related to the reorganization of its *Hox* cluster (i.e., the translocation of *Hox1-Hox3*; Fig. 2c).

### Saponin biosynthesis and convergent evolution

**Fig. 3:**
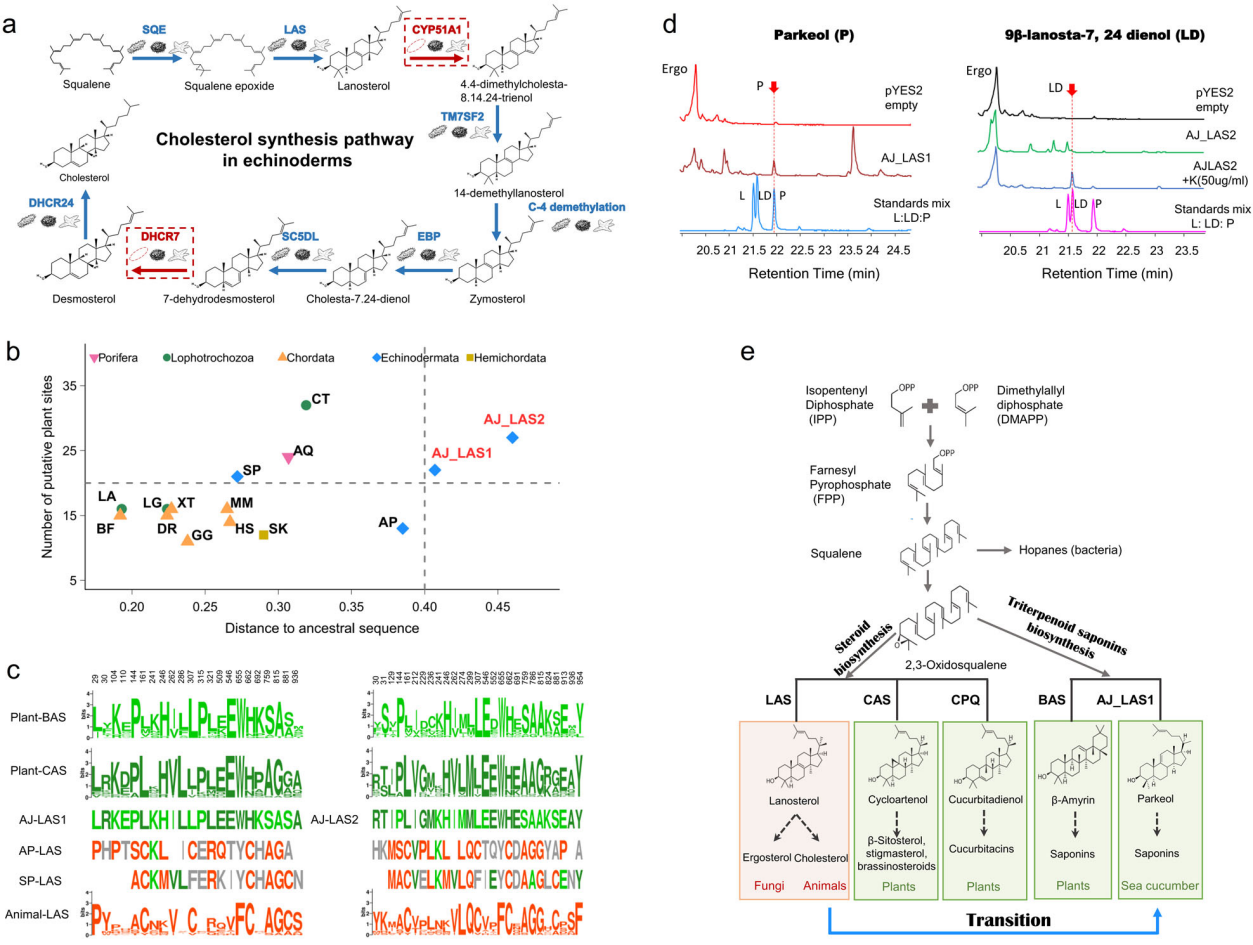
Saponin biosynthesis and convergent evolution of *LAS* genes in *A. japonicus*. **a** Gene representation of the canonical animal cholesterol synthesis pathway in the sea cucumber genome. The sea cucumber lacks two genes, *Cyp51* and *Dhcr7*, suggesting that it might have lost its de novo cholesterol synthesis ability, consistent with the previous observation of extremely low cholesterol content in sea cucumber^50^. **b** Evolutionary analysis of the lanosterol synthase (*LAS*) genes in sea cucumber and other animals. Compared to the inferred ancestral bilaterian LAS sequence (see Methods for details), LAS1 and LAS2 in sea cucumber show the highest sequence divergence and possess more putative plant sites than most other animals. **c** Overview of plant-like motifs in the sea cucumber LAS sequences and comparison with those of the animal consensus LAS sequence and plant consensus BAS and CAS sequences. The plant-like motifs in the sea cucumber LAS sequences are not present in sea urchin and starfish, suggesting the de novo acquirement of these motifs in the sea cucumber lineage. **d** Product determination by yeast expression of sea cucumber LAS1 and LAS2. Contrary to the general expectation that animal LAS produces lanosterol, neither sea cucumber LAS1 nor LAS2 produces lanosterol. Sea cucumber LAS1 produces parkeol (previously identified as the triterpene precursor of sea cucumber saponins;^51^), whereas LAS2 produces 9β-lanosta-7, 24 dienol. **e** Summary of the pathways leading to saponin biosynthesis and steroid biosynthesis. In contrast to the plant kingdom, saponin biosynthesis is rarely found in the animal kingdom^19,20^. The extraordinary ability of saponin synthesis in sea cucumber is enabled by its modification of lanosterol synthase, which possibly occurred through convergent evolution

Saponins are secondary metabolites that are commonly present in the plant kingdom but are only found in a few animal lineages, including sea cucumber^19^. Sea cucumber saponins (also known as holothurins) belong to the triterpene glycoside family of natural products^16^. Current knowledge about saponin biosynthesis has come mostly from plant studies^19,20^, and how sea cucumbers gained the ability to synthesize saponins is a source of intrigue. As both sterols and triterpenes are synthesized via the mevalonate (MVA) pathway^49^, we first investigated the integrity of the MVA pathway in sea cucumber for the route of cholesterol synthesis in animals, and we found that the two genes *Cyp51* and *Dhcr7* were absent in the sea cucumber genome (Fig. 3a; Supplementary Table S18). This finding, in contrast to the observation of full gene sets in sea urchin *S*. *purpuratus* and starfish *A*. *planci* (Fig. 3a; Supplementary Table S18), suggests that the sea cucumber *A*. *japonicus* might have lost the ability to synthesize cholesterol de novo, which is consistent with previous observations of extremely low cholesterol levels in sea cucumber^50^. In addition, the absence of *Cyp51* (i.e., C-14 sterol demethylase) in the sea cucumber genome also supports the previous speculation that the blockage of C-14 demethylation leads to the accumulation of 14α-methylated Δ^9(11)^-sterols in cell membranes of sea cucumber, contributing to resistance to their own toxins^15^.

Sterols and triterpenes share the common biosynthetic precursor 2,3-oxidosqualene, which can be cyclized by different oxidosqualene cyclases (OSCs) to produce sterols [lanosterol synthase (LAS) in fungi and animals and cycloartenol synthase (CAS)/cucurbitadienol synthase (CPQ) in plants] or triterpenes [e.g., β-amyrin synthase (BAS) in plants]^20^ (Fig. 3e). We found two predicted OSC genes (named in this study as *LAS1* and *LAS2*) in the sea cucumber genome (Supplementary Figures S7, S8). Evolutionary analysis suggests that the sea cucumber *LAS* genes show high evolutionary rates compared with diverse animal groups, and contain many plant-like motifs that are not present in sea urchin and starfish (Fig. 3b, c; Supplementary Figure S9). Contrary to the general expectation that cyclization of 2,3-oxidosqualene by animal LAS produces lanosterol, functional analysis of yeast expressing *A*. *japanicus* LAS1 and LAS2 revealed that neither produces lanosterol. Instead the main products were identified as parkeol (LAS1) and 9β-lanosta-7, 24-dienol (LAS2) (Fig. 3d; Supplementary Figures S10-S12). Parkeol has previously been suggested to be the triterpene precursor of saponins in sea cucumbers^51^, although whether 9β-lanosta-7, 24 dienol is also a saponin precursor remains to be determined. Previous studies suggested that the LASs of different eukaryotic lineages emerged from an ancestral plant-like CAS that is capable of producing both parkeol and cycloartenol^52^. A switch of LAS product specificity from lanosterol to parkeol may reflect the convergent evolution of sea cucumber LAS (prone to plant-like CAS; Fig. 3e), enabling the evolutionary appearance of effective chemical tools of defense in sea cucumbers.

### Key regulators and transcriptional network of aestivation

**Fig. 4:**
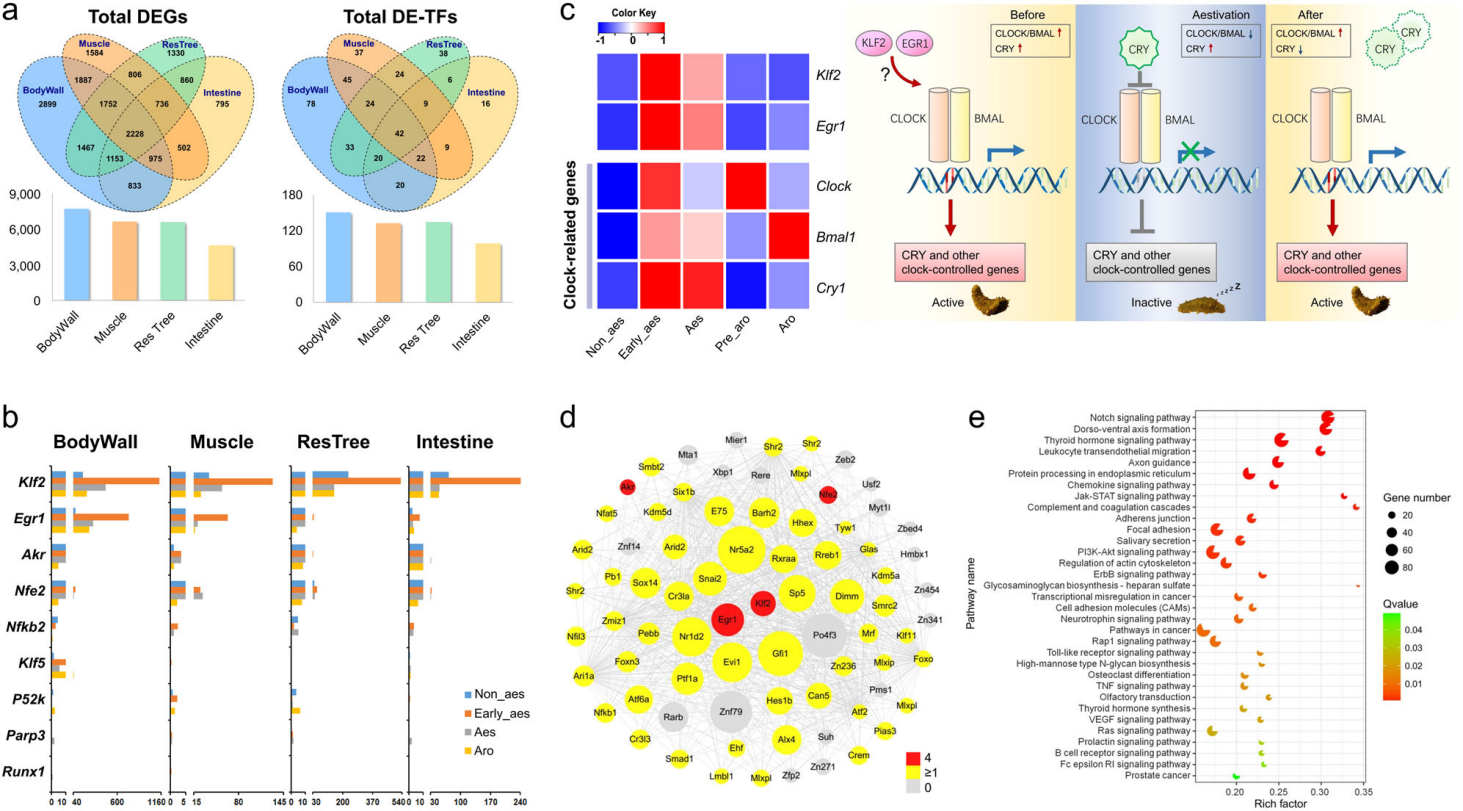
Key regulators and transcriptional network of aestivation in *A. japonicus*. **a** Identification of differentially expressed genes (DEGs) and differentially expressed transcriptional factors (DE-TFs) in four organs (body wall, muscle, respiratory tree and intestine) during different states of aestivation. Venn diagrams and histograms show the shared gene numbers between organs and the absolute gene numbers in each organ. Among the four organs, body wall shows the most DEGs and DE-TFs, representing the most responsive organ during sea cucumber aestivation. **b** Expression profiles of nine TFs showing differential expression during aestivation in all four organs. Compared with other TFs, *Klf2* and *Egr1* are the most significant TFs, especially in body wall, likely playing important roles in the regulation of aestivation. Aestivation states: non-aestivation (Non_aes); early aestivation (Early_aes); deep aestivation (aes); and arousal from aestivation (Aro). **c** Expression heatmap of *Klf2*, *Egr1* and clock-related genes during different aestivation states (according to quantitative PCR results), and the inferred clock gene-controlled regulation model. *Klf2* and *Egr1* may trigger the upregulation of *Cry1* (either directly or indirectly through *Clock* and *Bmal1*) during sea cucumber aestivation, which propels the animal into an extended sleep phase, and decreased *Cry1* expression makes the animal awaken from aestivation. Aestivation states are the same as depicted in (**b**) except Pre_aro representing initial arousal from aestivation. **d** Co-expression TF network of the aestivation-responsive model AM7. *Klf2* and *Egr1* are recognized as hub transcription factors in the network. The TFs showing differential expression in all organs are labeled in red, whereas for the remaining DE-TFs showing differential expression in at least one organ are labeled in yellow. **e** KEGG enrichment analysis of the AM7 module. The AM7 module governs diverse gene pathways, including those participating in cell proliferation and differentiation, seasonal rhythmicity and immune responses, suggesting the complex mechanism of molecular regulation during sea cucumber aestivation. The circle size and filled portion represent the gene numbers (from the AM7 module) and percentage of differentially expressed genes (DGEs) in a given pathway, respectively. The statistical significance is colored according to *Q* values

Studies on aestivation to date have focused primarily on terrestrial animals such as amphibians and land snails^53^, with relatively little attention paid to understanding animal aestivation in marine environments^23,54^. To understand the regulatory mechanism of sea cucumber aestivation (Supplementary Figure S13), we conducted large-scale transcriptome sequencing by generating 39 data sets (Supplementary Table S20) from multiple organs (body wall, muscle, respiratory tree and intestine) across different aestivation states. Body wall showed the most differential expressed genes (DEGs) and differentially expressed transcriptional factors (DE-TFs), followed by muscle, respiratory tree and intestine (Fig. 4a; Supplementary Figures S14, S15; Supplementary Tables S21–S25), suggesting that body wall may be more sensitive in response to thermal stress than other organs. Nine TFs showed differential expression during aestivation in all four organs, of which *Klf2* and *Egr1* were the most significant TFs especially in body wall (Fig. 4b). *Egr1* and members of the *Klf* family are known to participate in biorhythm regulation^55–58^, e. g., *Egr1* can regulate the clock gene *Cry1*^55^, the key repressor gene that can propel the animal into sleep phase (through the inhibition of key activators *Clock* and *Bmal1*^59^). We therefore investigated the expression patterns of clock-related genes during the aestivation of sea cucumber. To ensure a reliable evaluation and inference, two analytical approaches (RNAseq and quantitative PCR) were adopted, both of which produced largely consistent results (Fig. 4c; Supplementary Figure S16). Expression patterns of *Cry1* in body wall coincide with the aestivation states of sea cucumber, with increased expression during early and deep aestivation and decreased expression after aestivation (Fig. 4c; Supplementary Figure S16). The expression of key activators *Clock* and *Bmal1* is suppressed during deep aestivation (corresponding to the enhanced role of the repressor *Cry1*; Fig. 4c; Supplementary Figure S16), and is re-activated with emerging arousal from aestivation (corresponding to the decreasing effect of *Cry1*; Fig. 4c; Supplementary Figure S16). These observations suggest that sea cucumber aestivation, such as other types of dormancy^60,61^, might be a clock gene-controlled process with an extended sleep phase possibly triggered by *Egr1* and/or *Klf2* (Fig. 4c).

To understand the gene regulatory network of aestivation, we constructed a gene co-expression network using the 39 transcriptome data sets, and identified 6 aestivation-related modules, with AM7 as the most significant module across three organs (body wall, muscle and respiratory tree; Supplementary Figure S17; Supplementary Table S27). In particular, the transcription factors *Klf2* and *Egr1* were recognized as hub transcription factors in the AM7 network (Fig. 4d; Supplementary Table S28), further supporting their role as key regulators of aestivation. Pathway enrichment analysis revealed that the AM7 module governs complex molecular regulation involving with diverse gene pathways (Fig. 4e; Supplementary Table S29), including Notch, Jak-STAT and PI3K-Akt signaling pathways for cell proliferation, differentiation and apoptosis^62^, the thyroid hormone signaling pathway for seasonal rhythmicity^63^, the neurotrophin signaling pathway for neuronal survival, growth and differentiation^64^, and chemokine, complement and Toll-like receptor signaling pathways for immune responses^65^. Taken together, our study presents the first step to identify the key regulators and gene network for sea cucumber’s aestivation, and our novel findings provide insights into how sea cucumber optimizes its physiological states for long-term viability during aestivation.

### Regulation of intestine hypometabolism and regeneration

**Fig. 5:**
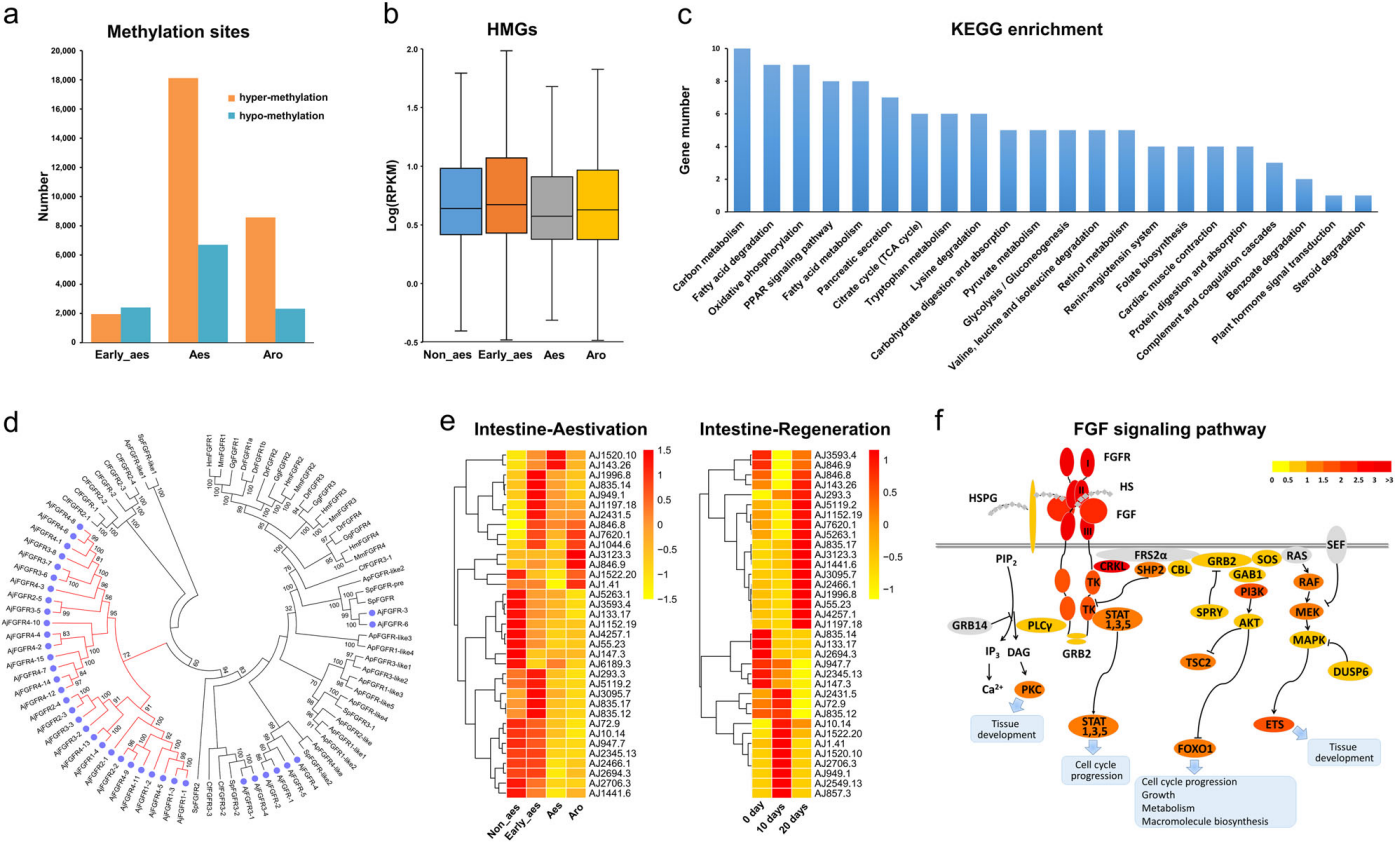
Epigenetic regulation of intestine hypometabolism and participation of the expanded *Fgfr* family in intestine regeneration of *A. japonicus*. **a** Identification of differentially methylated sites during different aestivation states. The intestine of sea cucumber shows prominent hypermethylation during aestivation. **b** Expression profiles of significantly hypermethylated genes (HMGs), showing the overall transcriptional suppression of these HMGs. **c** KEGG enrichment analysis of HMGs. HMGs are involved in numerous metabolic pathways, suggesting that intestine hypometabolism is caused by transcriptional suppression of metabolic pathways mediated through DNA hypermethylation. **d** The phylogeny of the *Fgfr* gene family in sea cucumber and other animals. The *Fgfr* gene family shows significant expansion in the sea cucumber genome (38 in contrast to 4–13 in other echinoderms or chordates). The expanded gene members in sea cucumber mostly form a separate clade (indicated by the red cluster). Numbers above the branches are support percentages for 1000 bootstrap replicates. The accession numbers or IDs of corresponding genes displayed in the tree are provided in Supplementary Table S37. **e** Expression heat maps of the *Fgfr* genes in sea cucumber intestine during different stages of aestivation and regeneration. Expression of the *Fgfr* genes is mostly suppressed during aestivation (corresponding to intestine atrophy), whereas it is activated during the regeneration process. **f** Expression of the FGF signaling pathway^72^ during intestine regeneration in sea cucumber. The potential roles of the FGF signaling pathway in intestine regeneration are supported by observation of the activation of various downstream cascades during the intestine regeneration process in sea cucumber. Fold-change (regeneration stages vs. the control stage) is color-coded

The intestine of sea cucumber has long attracted great research interests due to its extraordinary potential for degeneration and regeneration^23^. During aestivation, sea cucumber experiences intestine atrophy and depresses its global metabolic rate, and such intestine degeneration can fully recover after arousal from aestivation. Previous preliminary studies suggested that DNA methylation may play an important role during sea cucumber aestivation^66,67^, but to date there is still lack of direct evidence linking DNA methylation with hypometabolism and potential targeted genes remain to be uncovered. We conducted DNA methylome profiling of intestine samples from normal and different aestivation states using the MethylRAD technique^68^ and found that a remarkable increase in DNA methylation level during aestivation (Fig. 5a). We identified 411 hypermethylated genes (HMGs) during aestivation (Supplementary Table S30), 64% of which showed transcriptional suppression during aestivation (Fig. 5b). Functional enrichment analysis of the identified HMGs revealed that they are involved in numerous metabolic pathways such as carbon metabolism, fatty acid metabolism, pyruvate metabolism and retinol metabolism (Fig. 5c). This finding suggests that the phenomenon of intestine hypometabolism in sea cucumber results from transcriptional suppression of various metabolic pathways mediated through DNA hypermethylation.

To understand the transcriptional regulation of intestine regeneration, we conducted transcriptome profiling of normal and regenerated intestines at 10 and 20 dpe (days post-evisceration) (Supplementary Figure S18; Supplementary Table S31). We identified 6,511 DGEs (Supplementary Tables S32, S33; Supplementary Figure S19), and co-expression network analysis identified two regeneration-related modules that involved diverse signaling pathways, including Wnt and Hippo, which are well-known for participating in intestine regeneration in sea cucumber and other animals^69,70^ (Supplementary Figures S20-S24; Supplementary Tables S34-S36). Among the identified DGEs, the significant expansion of the fibroblast growth factor receptor (*Fgfr*) gene family in the sea cucumber genome (38 in contrast to 4–13 in other echinoderms or chordates; Fig. 5d) is of particular interest, as this family is known to be crucial for animal organogenesis and regeneration by mediating the FGF signaling pathway^71,72^. Expression analysis of *Fgfr* genes suggests that their transcription is mostly suppressed during aestivation (corresponding to intestine atrophy), whereas it is activated during the regeneration process (Fig. 5e). Their potential roles in mediating the FGF signaling pathway for regeneration are further supported by observation of the activation of various downstream cascades of this pathway during the intestine regeneration process (Fig. 5f).

## Discussion

Echinoderms exhibit several fascinating evolutionary innovations that are rarely seen in the animal kingdom, and how they attained such extraordinary biological features through evolution has been a long-standing unsolved mystery. Here we present a high quality of the sea cucumber *A*. *japonicus* genome assembly and extensive transcriptomes, with the assembly quality comparable or superior to many previously published echinoderm genomes^8,11,12^. Our analysis of the sea cucumber genome revealed novel genomic features and molecular changes that may contribute to the evolutionary appearance of striking biological features. The sea cucumber has a typical *Hox* cluster similar to that of sea star *A*. *planci*, invalidating the previous hypothesis that *Hox* clusters of all echinoderms are reorganized^39,40^. We also revealed that the spatial expression patterns of *Hox7* and *Hox11/13b* were potentially responsible for embryo-to-larva axial transformation in echinoderms. The extraordinary ability of sea cucumbers to make saponins is enabled by its modification of lanosterol synthase, which possibly occurred through convergent evolution. This implies that even for very complex metabolic pathways, modifying just one key gene could lead to the generation of a new adaptive trait in an organism. *Klf2* and *Egr1* were identified as putative key regulators during sea cucumber aestivation, probably exerting their effects through a clock gene-controlled process. While circadian regulation has been extensively studied in many organisms, the exact ways of how these key regulators exert their effects in sea cucumber’s aestivation and whether in the same way as in circadian regulation remain to be thoroughly explored. We found that intestine hypometabolism during aestivation is driven by the DNA hypermethylation-mediated expression suppression of various metabolic gene pathways, providing direct evidence linking DNA methylation with hypometabolism. The intestine regeneration involves diverse signaling pathways including Wnt, Hippo and FGF. The outstanding expansion of the *Fgfr* gene family may promote regeneration potential and contribute to the extraordinary regeneration capacity of sea cucumber. The sea cucumber genome, together with extensive transcriptomes, represents an invaluable resource and provides a new avenue for understanding the evolutionary appearance and molecular regulation of these extraordinary biological features in sea cucumbers and other echinoderms.

## Materials and methods

### Genome sequencing and genome size estimation

Genomic DNA was extracted from the gonad tissue of a wild female sea cucumber individual using the conventional phenol/chloroform extraction method^73^. To address the challenge of high genome heterozygosity in echinoderms^8^, we used a combinational sequencing strategy both with traditional paired-end/mate pair sequencing and long read sequencing. Consequently, we constructed short-insert paired-end libraries (180 bp, 350 bp, 450 bp, 500 bp and 550 bp) using the Illumina standard protocol (San Diego, USA) and long-insert mate-pair libraries (5 Kb, 10 Kb, 15 Kb and 20 Kb) following the Cre-lox recombination-based protocol^74^. Then these libraries were sequenced using an Illumina HiSeq 2000 platform, which produced 349.34 Gb of raw data. Long read sequencing was done by a Pacific Biosciences (PacBio) system, which generated 23.22 Gb of raw data with an average read length of ~9 Kb. After quality filtering, 352.38 Gb of high-quality data were used for genome assembly.

The genome size of *A*. *japonicus* was estimated using flow cytometry^75^ and k-mer analysis^76^. Flow cytometry analysis of *A*. *japonicus* was conducted as previously described^75^ using the scallop *Chlamys farreri* as an internal reference standard. The distribution of 19-mers was calculated based on paired-end reads derived from the DNA libraries with insert sizes of 180 bp, 350 bp and 500 bp. The genome size was estimated using the following formula:^77^ genome size = (total number of 19-mers) / (position of peak length).

### Genome assembly and genome-quality assessment

To conquer high genome heterozygosity, we adopted a hybrid assembly strategy to generate long genome contigs. The genome assembly backbone was first constructed using Illumina short-insert paired-end reads by MaSuRCA with its default parameters^78^, and the long PacBio sequencing reads were recruited to link and extend the backbones by SSpace-long^79^ with parameters –k 1 –l 1 and PBJelly2^80^. A homemade Perl script was used to recover part of abandoned sspace-LongRead links due to strict internal settings, which led to an improved assembly. Due to the unsatisfactory scaffolding efficiency of PacBio long reads for the sea cucumber genome^8^, we also adopted a classic strategy using traditional mate pair sequencing in the scaffolding step. In total, 140.85 Gb of Illumina mate pair sequencing reads with large insert lengths ranging from 5 kb to 20 kb were used to connect and integrate the assembled contigs into scaffolds through SSpace-standard with parameters −k 2 −a 0.7 −x 0 −m 50^79^. A total of 1,238,063 assembled mRNAs contigs were also used to evaluate the gene coverage of the genome assembly.

### Linkage map-based chromosome anchoring

A total of 3,063 2b-RAD^81^ marker sequences were obtained from a high-density linkage map of *A*. *japonicus*^28^ and aligned back to the assembly using SOAP^82^ with the parameter settings of -M 4 –r 0 –v 2. Only markers with a unique location were used for anchoring and orienting scaffolds to different linkage groups. Scaffolds in conflict with the genetic map (such as markers from a different linkage on the same scaffold) were checked manually with 10 kb mate-paired reads.

### Genome annotation

Repeat sequences were predicted via two approaches (homology-based method and de novo prediction). RepeatMasker was used for homology-based transposable element (TE) prediction: we ran searches against RepBase sequences with default parameters^83^. De novo TEs in the genome were detected by RepeatMasker based on a de novo repeat library, constructed by RepeatModeller (http://www.repeatmasker.org/RepeatModeler.html). These unclassified elements were further classified using the following strategy: (a) Unknown repeat sequences were aligned to Repbase using blastn; (b) Remaining unknown repeats were aligned to RepBase using tblastx; (c) Remaining unknown repeats were aligned to Uniprot database; and (d) Remaining repeats were classified by TE-class using multiple classification methods. The results from the above strategies were combined to generate final repeat database.

To predict the genes in the *A*. *japonicus* genome, three approaches (de novo, transcriptome-based and homolog-based predictions) were employed. For de novo predictions, we used three *ab initio* gene prediction tools—FGENESH using settings of printmRNA = yes, printExons = yes, Organism = Sea Urchin / *S. purpuratus*^84^, GENEMARK with --ES parameter^85^ and Augustus^86^, to predict coding genes. A primary model predicted by CEGMA was used as an Augustus training set. To improve the prediction accuracy, 7,739 *A*. *japonicus* expressed sequence tags (ESTs) with a total length of 1.171 Mb were obtained from NCBI. These ESTs were integrated with unigenes assembled by Trinity^87^ and then used to train a prediction model in PASA^88^ with parameters of -C -R --ALIGNERS blat. Protein sequences from twelve sequenced genomes (*Branchiostoma* *floridae*, *Caenorhabditis* *elegans*, *Crassostrea* *gigas*, *Ciona* *intestinalis*, *Drosophila* *melanogaster*, *Danio* *rerio*, *H*. *robusta*, *Helobdella* *sapiens*, *Mus* *musculus*, *Nematostella* *vectensis*, *Patinopecten* *yessoensis*, and *S*. *purpuratus*) were downloaded from Ensembl (Release 85) or JGI or EchinoBase. The protein sequences of the selected species were aligned to the repeat-masked sea cucumber genome using the Exonerate^89^ tool with parameters of --model protein2genome --refineboundary 1000 --showvulgar no --showalignment no --showquerygff no --showtargetgff yes.

The non-redundant consensus set of gene structures was integrated in EVidenceModeler (EVM)^90^ using all the gene evidence predicted above with parameters of --segmentSize 100000 --overlapSize 10000. The locations of the untranslated regions (UTRs) were added using PASA^88^ based on transcripts alignment. Next, we removed the gene models that were not supported by protein or transcript alignment, or were not supported by more than 2 de novo prediction methods. In total, 29,451 protein-coding genes were retained, constituting the final gene set of *A*. *japonicus*.

Functional annotation of protein-coding genes was first performed by means of BLASTP (*E* value threshold: 1e–05) against the protein databases SwissProt and Nr. Annotation information from the best BLASTP hits was retained for the sea cucumber gene set. Protein domains were annotated by searching the InterPro (v29.0) database. Gene Ontology (GO) terms for each gene were retrieved from the corresponding InterPro entry. The sea cucumber gene set was also mapped to the KEGG pathway database (release 53) to identify the best match for each gene.

### Transcriptome analysis of developmental stages

Embryos (zygote, blastulae and gastrulae), larvae (auricularia, doliolaria, and pentactula) and juveniles of *A*. *japonicus* were collected based on artificial fertilization of sex-matured adults and larval cultivation according to Zhang et al.^91^. Total mRNA was extracted from each sample ( > 1000 embryos/larvae per developmental stage) by following the protocol described by Du et al.^27^. All RNA-seq libraries were constructed using the NEB Next mRNA Library Prep Kit by following the manufacturer’s instructions and then were subjected to paired-end 100-bp (PE100) sequencing on the Illumina HiSeq 2000 platform. Sequencing reads were aligned to the *A*. *japonicus* genome using STAR aligner^92^ with its default parameters. Gene expression levels in terms of RPKM were estimated by HTseq^93^ and custom Perl scripts.

### Polymorphism analysis

To characterize the nucleotide polymorphism in the *A*. *japonicus* genome, reads from eight resequenced individuals were aligned to the assembled genome using BWA software^94^ with the settings of -n 15 -o 1 -e 10. Afterwards, SAMtools^95^ was used to sort alignments and filter PCR duplicates. SNPs were called using SAMtools mpileup and bcftools with a minimal mapping quality of 50, and sites with a read depth lower than 4 or higher than 4 times the average sequencing depth were filtered out.

SNP density was defined as the number of SNP sites per unit region among all 6 sequenced individuals. SNP density across the genome was calculated in 1-Mb sliding windows with a step size of 50 kb, and SNP density in the CDS region of each gene was also estimated. Genomic regions or CDSs with high SNP density were subjected to one-sided Fisher’s exact test compared to the corresponding chromosomal background, and the cutoff *p*-value was set to 1e–4. The distribution of SNP density in chromosomes or genes was visualized using Circos software^96^.

### Homeobox gene analysis

To identify the *Hox* and *ParaHox* genes, homeodomains were searched in the *A*. *japonicus* genome using BLAST with an *E* value threshold of 1e−5 against all homeodomain sequences retrieved from the HomeoDB database (http://homeodb.zoo.ox.ac.uk/)^97^ and were further confirmed by comparing the results to the Conserved Domains Database (http://www.ncbi.nlm.nih.gov/cdd). Homeobox genes were classified based on BLAST results, molecular phylogeny and manual inspection of conserved residues. The homeodomain regions of *Hox* genes were used to construct the phylogenetic tree using the Neighbor-Joining method^98^ in MEGA7^99^. Evolutionary distances were computed using the p-distance method^100^. All positions containing gaps and missing data were eliminated and the robustness of the resulting phylogenies was tested by a reanalysis of 1,000 bootstrap replicates^101^. Heat maps of *Hox* and *ParaHox* gene expression were drawn using custom R scripts that call the heatmap.2 function of gplots.

### Evolutionary analysis of MVA pathway genes in echinoderms

Known genes participating in the human MVA pathway were downloaded from NCBI protein database and aligned to the full gene sets of three echinoderms (*A*. *japonicus*, *S*. *purpuratus* and *A*. *planci*) by BLASTP with 1e-5. BLAST hits in every echinoderm species were further checked by domain searching to ensure the presence of expected protein domains.

OSC (BAS, CAS, LAS) protein sequences in animals and plants were first aligned using MEGA 7.0^99^, and positions with more than two-thirds gaps were removed. The amino acid percentage in every remaining position was calculated for plant BAS genes, plant CAS genes and animal LAS genes. The amino acids found in an animal lineage with a percentage smaller than 20% in animal LAS genes and larger than 55% in plant BAS or CAS genes, were defined as plant-like sites. The ancestral animal sequence of the LAS gene was deduced based on the following criterion: the amino acid was the predominant type in at least two of three animal groups (non-bilaterian, protostomia and deuterostomia). The distance between the LAS gene in every animal species and the ancestral LAS sequence was calculated using MEGA 7.0^99^ with the parameters of bootstrap method (1000 replications), Poisson model and pairwise deletion.

### Functional analysis of AJ-LAS genes using yeast expression system

#### Gene synthesis

For the two OSC sequences (AJ-LAS1 and AJ-LAS2) identified in the *A*. *japonicus* genome, we opted to synthesize them as gblocks from IDT (Integrated DNA Technologies) since their sequences were of good quality and appropriate length. Each of the OSCs was divided into two ~1.2 Kb fragments with an overlapping region of 50 bp in the middle (Supplementary Table S19). We employed this strategy to allow homologous recombination of these two fragments along with an empty vector resulting in the construction of an in-frame expression vector in the yeast cell.

#### Yeast cloning

All cloning and expression analysis was carried out in the yeast strain GIL77 (gal2 hem3-6 erg7 ura3-167)^102^. Expression vectors were constructed using in vivo homologous recombination in yeast. The open reading frames (ORFs) of OSCs were amplified from gblocks using the oligonucleotides listed in Supplementary Table S19. Each primer contained a region that overlapped with the pYES2 vector sequences (the 5′ end of the forward primer overlapped with the GAL1 promoter sequence, and the 5′ of the reverse primer overlapped with the CYC1 terminator sequence). The 3′ ends of the primers matched the beginning and end of the respective OSCs gblock fragments. The gblocks of each of the OSCs were amplified using the primers listed in Supplementary Table S19. The obtained PCR fragments were co-transformed into the GIL77 strain along with XbaI/HindIII-linearized pYES2 vector. Yeast transformation was performed using standard protocols (Yeastmaker™ Yeast transformation system 2, Clontech Laboratories). This resulted in vivo recombination between the pYES2 vector and the gblock OSC fragments. Plasmids were recovered from yeast transformants, transformed back into *E*. *coli* and sequence verified by sequencing the whole gene using three different primers spanning the entire length of the gene. The sequencing primers are listed in the Supplementary Table S19.

#### Yeast expression

For expression analysis, yeast strains were grown at 28 °C in 5 ml cultures in selective medium (SD-URA + 2% glucose + supplements) until saturation (~2 days). The supplements included the following: ergosterol (Fluka), 20 µg/ml; hemin (Sigma-Aldrich), 13 µg/ml; and Tween- 80 (Sigma-Aldrich), 5 mg/ml. Cells were then pelleted, washed in ddH_2_O, transferred to induction medium (SD-URA + 2% Galactose) and incubated for an additional 2 days for the accumulation of triterpenes. They were then pelleted and washed once with ddH_2_O before triterpene extraction.

#### Triterpene extraction and GC-MS analysis

The yeast pellets were mixed with 0.5 ml saponification reagent (20% KOH in 50% ethanol) and incubated at 65 °C for 2 h before extraction with an equal volume of hexane. The extraction step was repeated two additional times to maximize triterpene recovery. The extract was then dried down and the residue was dissolved in 500 µl of hexane. For rapid qualitative analysis, extracts were run on TLC plates (Silica gel on Al foil, 10 cm × 5 cm, FLUKA, Cat #70644) using a hexane: ethyl acetate (6:1) solvent system. Compounds were visualized by spraying the plates with acetic acid: H_2_SO_4_: p-anisaldehyde (48:1:1 v/v) and heating to 120 °C for 5 min on a TLC plate heater. For GC-MS analysis, 100 µl aliquots of hexane extract were transferred to 150 µl inserts and subjected to GC-MS analysis. Samples were run on a HP-5MS column (30 m × 0.25 mm i.d., 0.25 µm film) (Agilent). The injector port, source and transfer line temperatures were set at 250 °C and an oven temperature program from 80 °C (2 min) to 290 °C (30 min) at 20 °C/min was used. The carrier gas was helium and the flow rate was 1.2 ml/min. Samples were injected in splitless mode with 3 µl sample volume. The output was used to search the NISTv8 library to assign identity to peaks in the GC-MS traces. Product abundance was calculated as the percentage of total cyclic products using integrated peak areas. All experiments were repeated to confirm the reproducibility of triterpene profiles.

#### Inhibition of endogenous lanosterol-14α demethylase activity

Initial LAS1 or LAS2 expression experiments indicated a high degree of modification of the LAS1 products by endogenous sterol pathway enzymes. Here we hypothesized that the step immediately downstream of lanosterol synthase in yeast involves lanosterol-14α demethylase (CYP51, erg11), which might be a limiting step in further downstream modifications. Inhibition of erg11 might lead to an accumulation of LAS1 products without further modifications. Initial optimization experiments revealed that 50 µg/ml ketoconazole was sufficient for the complete inhibition of endogenous erg11 activity. Ketoconazole was dissolved in DMSO and applied to yeast cultures during the induction phase of the culturing process.

#### Triterpene standards

Lanosterol (Cat# L5768) was purchased from Sigma-Aldrich, whereas parkeol and lanostadienol were purified and characterized according to published literatures^103,104^. The standards were dissolved and diluted to 0.5 mg/ml in hexane and used in GC-MS. Ketoconazole (Catlog # K1003) was purchased from Sigma dissolved in DMSO and used in yeast media.

### Transcriptome analysis of sea cucumber aestivation

Adult sea cucumbers (80–120 g body weight) were collected from the coast of Liaoning, China (121°33′47″E, 38°51′55″N). The animals were acclimated in seawater aquaria (~500 l) at 15 °C for one week before use and were fed mixed feed once a day during this period. The feed ingredients included fresh sea mud (40%), *Sargassum thunbergii* (30%), and sea cucumber compound feed (30%, An-yuan company, China). Several individuals were maintained as the non-aestivation control group, and the other were slowly induced into aestivation by increasing the water temperature from 15 °C to 25 °C at a rate of 0.5 °C per day. Animals in this state were labeled as the early aestivation group. The rest were maintained at 25 °C for 15 days, after which some of the animals were sampled as the deep-aestivation group. The remaining animals were subjected to a decrease in water temperature back to 18 °C (at a rate of 0.5 °C per day), held at 18 °C for 2 days and then sampled as the arousal-from-aestivation group^105^. For each state, transcriptome sequencing was independently conducted for two to three individuals (i.e., biological replicates) to ensure reliable quantification of gene expression. Total mRNA was extracted from four organs (body wall, muscle, respiratory tree and intestine) following the protocol described by Du et al^27^. All RNA-seq libraries were constructed using the NEB Next mRNA Library Prep Kit following the manufacturer’s instructions and then were subjected to paired-end 100-bp (PE100) sequencing on the Illumina HiSeq 2000 platform. Sequencing reads were aligned to the *A*. *japonicus* genome using STAR aligner^92^ with its default parameters. Gene expression levels in terms of RPKM were estimated by HTseq^93^ and custom Perl scripts.

The transcription factor (TF) sequences of 8 selected animals (*H. sapiens*, *M. musculus*, *Gallus gallus*, *Xenopus tropicalis*, *D. rerio*, *D. melanogaster*, *C. elegans* and *C. intestinalis*) were downloaded from AnimalTFDB 2.0 (http://bioinfo.life.hust.edu.cn/AnimalTFDB/) and Ensembl (http://asia.ensembl.org/index.html), and the homologous proteins in sea cucumber were identified by comparing these known TF sequences against the A. japonicus genome using the BLAST algorithm with an *E* value threshold of 1e-10. The obtained candidate genes were further verified based on their annotations. Differentially expressed genes (DEGs) and differentially expressed TFs (DE-TFs) were detected using the edgeR package (*p* < 0.05)^106^.

The co-expression gene network was constructed by WGCNA software^107^ using 39 gene expression data sets from four organs (body wall, muscle, respiratory tree and intestine) across four different states (non-aestivation; early aestivation; deep aestivation; and arousal from aestivation), with the parameters of minimum module size = 300, cutting height = 0.997, and deepSplit = F. Cytoscape^108^ was employed for visualization of the co-expression network. To identify the aestivation-related module, over-representation analysis of the aestivation DEGs was performed for each module using a hypergeometric test with *p* values adjusted by the Benjamini–Hochberg method for multiple-test correction. The hubness of a gene in the aestivation-related module (AM7) was measured by its connection strength with other genes in the module and was determined by intra-modular connectivity (K_within_)^109^. KEGG enrichment analysis of AM7 was performed using the EnrichPipeline^110^. Directed acycline graphs (DAG) of GO terms corresponding to biological process were generated using OmicShare tools (www.omicshare.com/tools).

### Quantitative PCR validation

To further verify the expression patterns of aestivation-related genes (*Klf2*, *Egr1*, *Clock*, *Bmal1* and *Cry1*), quantitative PCR (qPCR) analyses were conducted. As we ran out of original samples for RNAseq analyses, another set of aestivation-related samples was adopted for qPCR validation. The adult sea cucumbers were acclimated in seawater aquaria (~500 l) at 15 °C for 1 week before use and were fed mixed feed once a day during this period. Several individuals were maintained at 15 °C as the control non-aestivation group, and the others were slowly induced into aestivation by increasing the water temperature from 15 °C to 22 °C at a rate of 1 °C per day and held at 22 °C for 1 week. According to the degree of intestine degeneration, animals in this state were labeled as the early aestivation group. The rest were maintained in water with the temperature increased to 28 °C at a rate of 1 °C per day and held at 28 °C for 1 week, and some of the animals in this state with complete degeneration of intestines were sampled as the deep-aestivation group. The remaining animals were subjected to a decrease in water temperature back to 22 °C (at a rate of 1 °C per day) and held at 22 °C for 1 week, and animals in this state were sampled as the pre-arousal group according to the degree of intestine restoration. The water temperature was continuously decreased to 15 °C at a rate of 1 °C per day and held at 15 °C for 1 week, and then, sea cucumbers with complete restoration of intestines were sampled as arousal from aestivation animals. For each state, total mRNA was extracted from body wall using an RNeasy Lipid Tissue Mini Kit (QIAGEN). Real-time PCR was conducted using the SYBR® Premix Ex Taq™ II (Tli RNaseH Plus) on an ABI7500 real-time PCR System. The running program was as follows: 30 s at 95 °C, followed by 40 cycles of 15 s at 95 °C, 35 s at 55 °C and 25 s at 72 °C. *Dpolm* and *Grb2* were used as an endogenous control for the normalization of gene expression^111^. Gene-specific primers were designed using Primer Premier 5.0, and the primer sequences are listed in Supplementary Table S26. For each gene of interest, 12 samples per stage were assayed. All PCR reactions were conducted in triplicate. Melt curve analysis was performed at the end of each PCR to confirm PCR specificity. The mRNA expression of each gene was quantified relative to that of reference genes *Dpolm* and *Grb2* using the ΔΔCt method. Grubbs test was used for the detection of outliers. Statistical analyses of the data were performed with the SPSS (version 16.0) statistical software package using independent t-tests. Differences were considered significant if *p* *<* 0.05.

### DNA methylation analysis of intestine during aestivation

Genomic DNA was extracted from intestine (three samples at each aestivation state) using the standard phenol/chloroform extraction method. MethylRAD libraries were constructed by following the protocol described by Wang et al.^68^ and were subjected to single end sequencing (1 × 50 bp) on an Illumina HiSeq2500 sequencer. Raw reads were first preprocessed to remove any sequences with ambiguous basecalls (N), long homopolymer regions (>10 bp) and excessive low-quality positions (>20% positions with quality score < 10). Then, high-quality reads were mapped to the target MspJI sites extracted from the *A*. *japonicus* genome using the SOAP program (parameters: -r 0 –v 2 –M 4). The sites detected in at least one group of three samples with coverage > 2 were used for differential DNA methylation analysis. The sum of all methylation sites in one gene was used to define its methylation level. Differential DNA methylation analysis between groups was conducted based on the method described by Wang et al^68^. Hyper-methylation genes with an average RPKM > 2 were used for KEGG enrichment analysis by EnrichPipeline^110^.

### Transcriptome analysis of intestine regeneration

Adult sea cucumbers (70–100 g) were collected from the coast of Liaoning, China (121°33′47″E, 38°51′55″N) and acclimated in seawater aquaria (~500 l) at 15 °C for 2 weeks prior to treatment, while being fed mixed feed once a day. After acclimation, the regeneration group (30 sea cucumbers) was treated to induce evisceration by intra-coelomic injection of 0.35 M KCl. The animals were left undisturbed in aquaria to allow regeneration for 10 days, which corresponded to cell dedifferentiation stages for intestine regeneration. Intestine tissues of two or three individuals from the non-injured group, the 10-day regeneration group and the 20-day regeneration group were collected for transcriptome sequencing. Total mRNA was extracted following the protocol described by Du et al^27^. All RNA-seq libraries were constructed using the NEB Next mRNA Library Prep Kit following the manufacturer’s instructions and then were subjected to paired-end 100-bp (PE100) sequencing on the Illumina HiSeq 2000 platform. Sequencing reads were aligned to the *A*. *japonicus* genome using STAR aligner^92^ with its default parameters. Gene expression levels in terms of RPKM were estimated by HTseq^93^ and custom Perl scripts.

Differentially expressed genes (DEGs) in intestine regeneration were detected according to the procedure described in the edgeR package (*p* *<* 0.05)^106^. GO and KEGG enrichment analysis of DEGs was performed using the EnrichPipeline^110^. The genes in all stages of samples with sum (RPKM) > 2 were used for network construction by WGCNA^107^ with the parameters of minimum module size = 300, cutting height = 0.99, and deepSplit = F. To identify the regeneration -related module, over-representation analysis of the regeneration DEGs was performed for each module using a hypergeometric test. Cytoscape^108^ was employed for visualization of the co-expression networks. KEGG enrichment analysis was performed using the EnrichPipeline^110^.

### Phylogenetic analysis of *Fgfr* genes

To understand the phylogeny of *Fgfr* genes of *A*. *japonicus*, the homologs from several selected animals including sea urchin (*S. purpuratus*), scallop (*C. farreri*), human (*H. sapiens*), mouse (*M. musculus*), chicken (*G. gallus*) and zebrafish (*D. rerio*), were retrieved from NCBI (http://www.ncbi.nlm.nih.gov) and Ensembl (http://useast.ensembl.org). All the retrieved FGFR amino acid sequences were used for phylogenetic analysis with the *A*. *japonicus* FGFRs. Phylogenetic trees were constructed using MEGA7 with the Neighbor-Joining method^98,99^. Bootstrapping with 1000 replications was conducted to evaluate the robustness of the phylogenetic tree.

### Data availability

This genome project has been registered in NCBI under the BioProject accession no. PRJNA413998. The sequencing data of the genome, transcriptomes and methylomes have been deposited in NCBI Sequence Read Archive under the accession numbers. SRX3311475, SRX3311477, SRX3311479-SRX3311482, SRX3311484, SRX3311485, SRX3311487, SRX3311488 (genome); SRX3302890-SRX3302909, SRX3299141-SRX3299148 (transcriptomes) and SRX3311539 (methylomes).

## Electronic supplementary material

Supplementary Information

Table S5

Table S14

Table S16

Table S18

Table S21

Table S30

Table S32

Table S37
