# Supplementary material for: Sea cucumber genome provides insights into saponin biosynthesis and aestivation regulation
Source: Cell Discov. 2018 Jun 26;4:29. doi: 10.1038/s41421-018-0030-5 (PMC6018497; doi:10.1038/s41421-018-0030-5)
Supplement: Supplementary file 1 — Supplementary Information [file 41421_2018_30_MOESM1_ESM.pdf]

Supplementary Materials for

Sea cucumber genome provides insights into saponin biosynthesis and aestivation regulation

This PDF file includes:

Supplementary Figures

Figures S1 to S24

Supplementary Tables

Tables S1 to S4, S6 to S13, S15, S17, S19, S20, S22-S29, S31, S33-S36

References (1-7)

Other Supplementary Materials for this manuscript include the following:

Table S5, S14, S16, S18, S21, S30, S32, S37

Supplementary Figures.

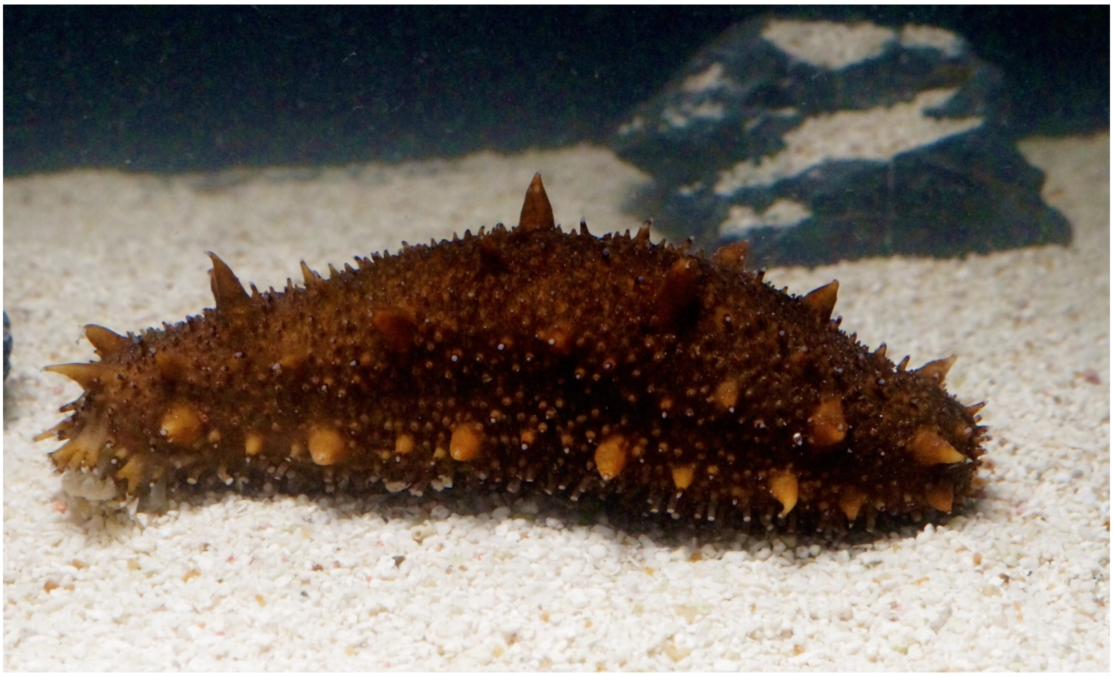

Figure S1 The photograph of the sea cucumber, *Apostichopus japonicus.*

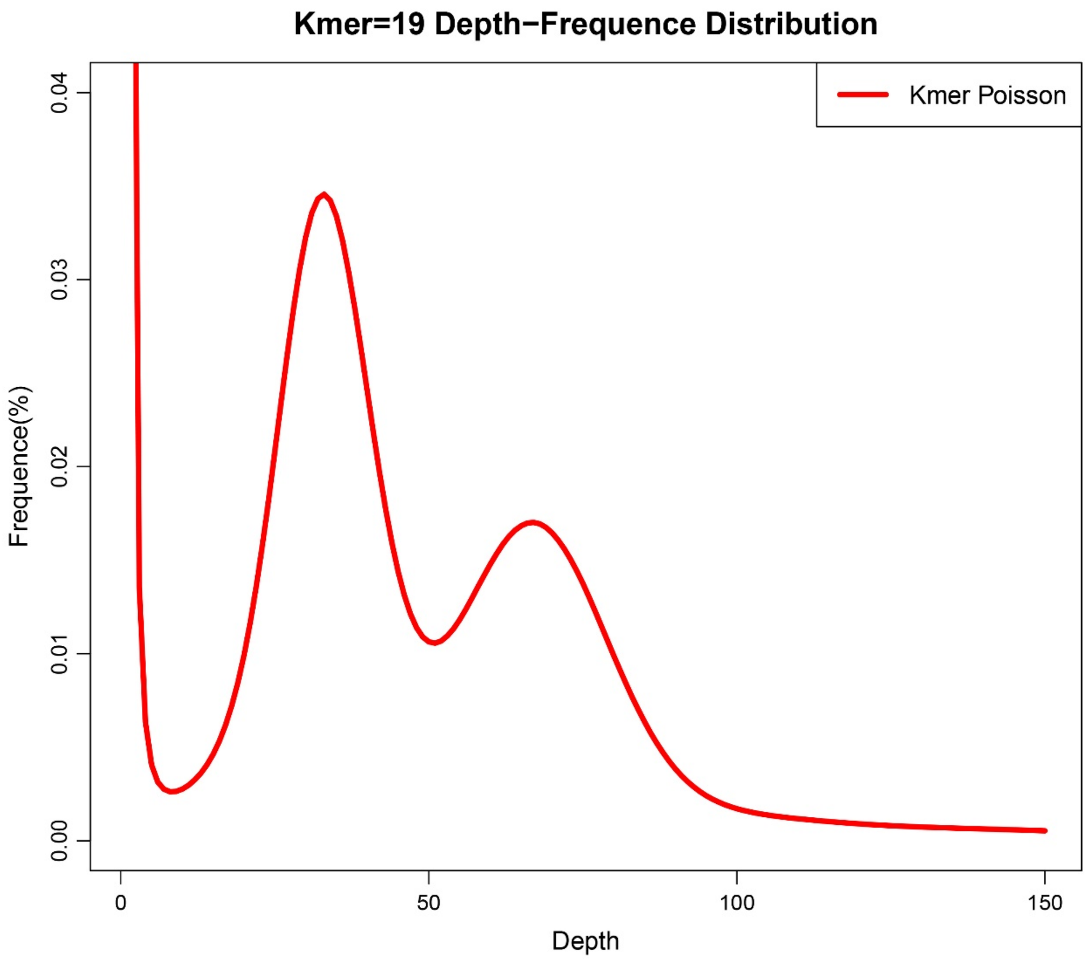

Figure S2 Distribution of 19-mer frequency in *A. japonicus* genome.

The 19-mer depth distribution curve was generated from high-quality short-insert reads of 180 bp, 300bp and 500bp. This K-mer distribution can be used to roughly determine the heterozygous rate and the genome size. If the heterozygous rate is higher, then another peak will be presented at 1/2 of Peak depth. Two peaks were observed at 33× and 67× respectively.

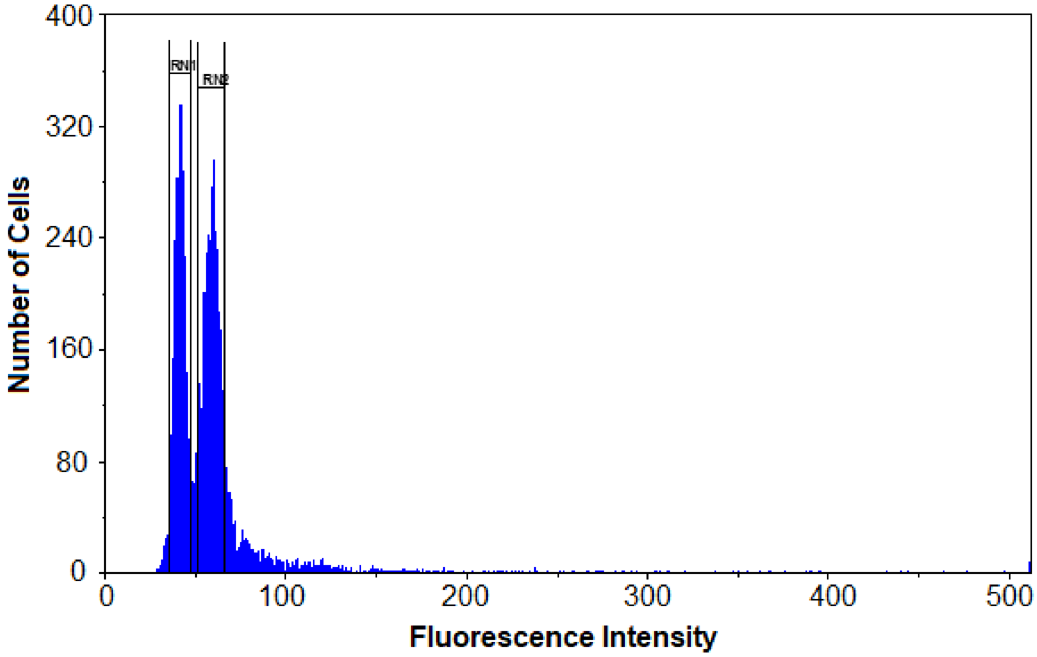

Figure S3 Genome size determination by flow cytometry.

The genome size of *A. japonicus* is estimated by the flow cytometry (0.878pg/1C, left peak), which is equivalent to 0.852 gigabases (Gb) per haploid genome based on the formula: 1 pg=0.978 Gb [1]. The scallop *C. farreri* (1.273pg/1C, right peak) was used as an internal reference standard.

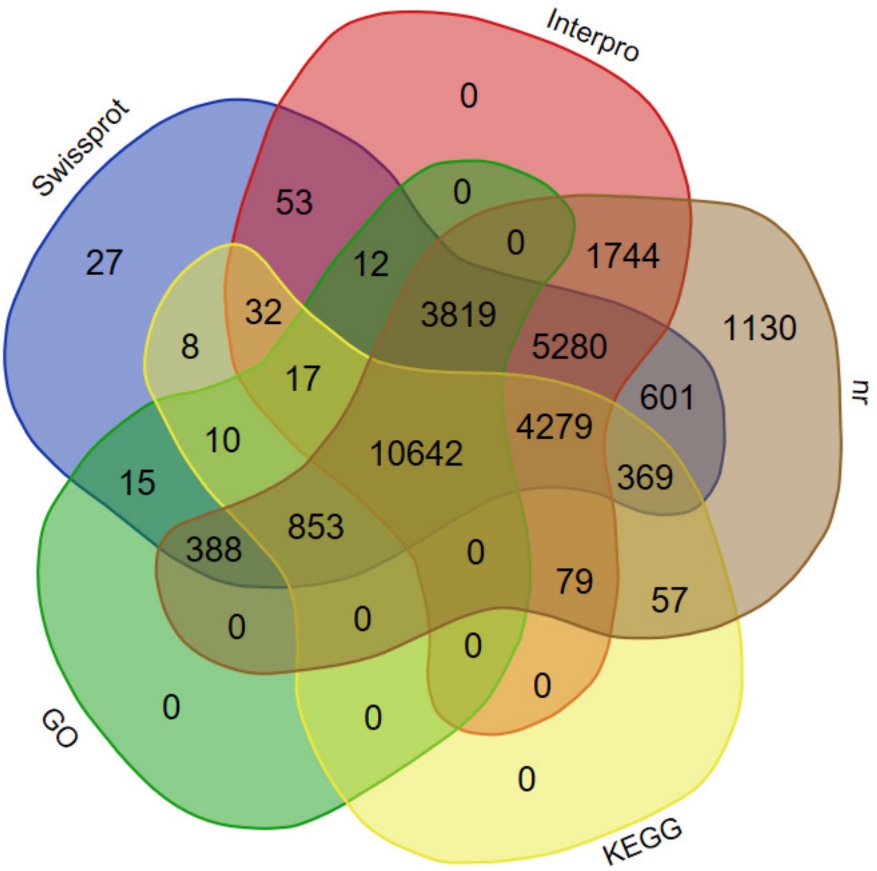

Figure S4 Gene Function Annotation supported by different databases.

Venn diagram of gene function annotation showed that over 96% of 29,451 genes are annotated by at least two databases

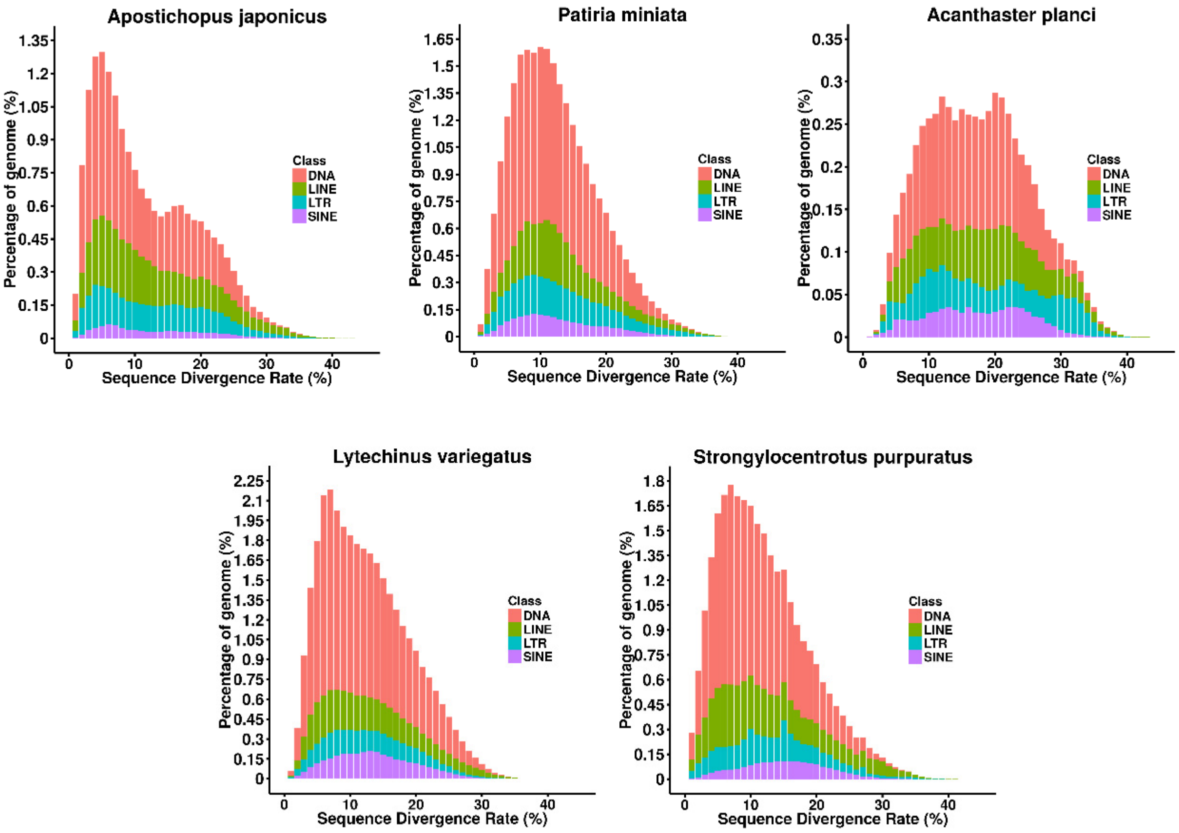

Figure S5 Divergence rates of transposable elements (TEs).

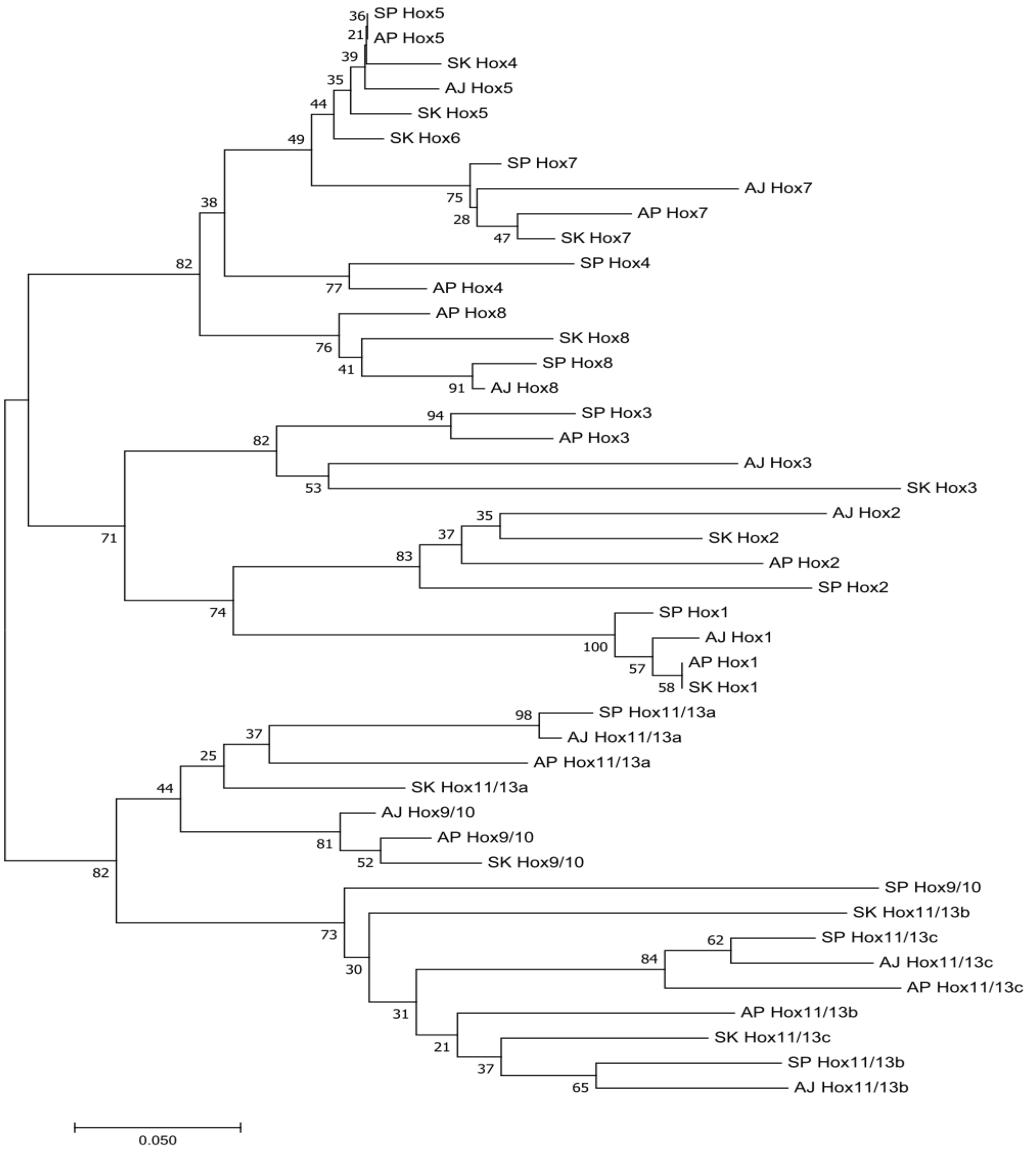

Figure S6 Phylogenetic relationships among ambulacraria *Hox* genes.

The homeodomain regions of *Hox* genes are adopted to construct the phylogenetic tree using the Neighbor-Joining method [2]. The evolutionary distances were computed using the p-distance method [3] and are in the units of the number of amino acid differences per site. Numbers above branches are supporting percentages of 1,000 bootstrap replicates [4]. Abbreviations of species in ambulacraria (echinodermata and hemichordata): AJ: *A. japonicus*, SP: *Strongylocentrotus purpuratus*, AP: *Acanthaster planci*, and SK: *Saccoglossus kowalevskii*.

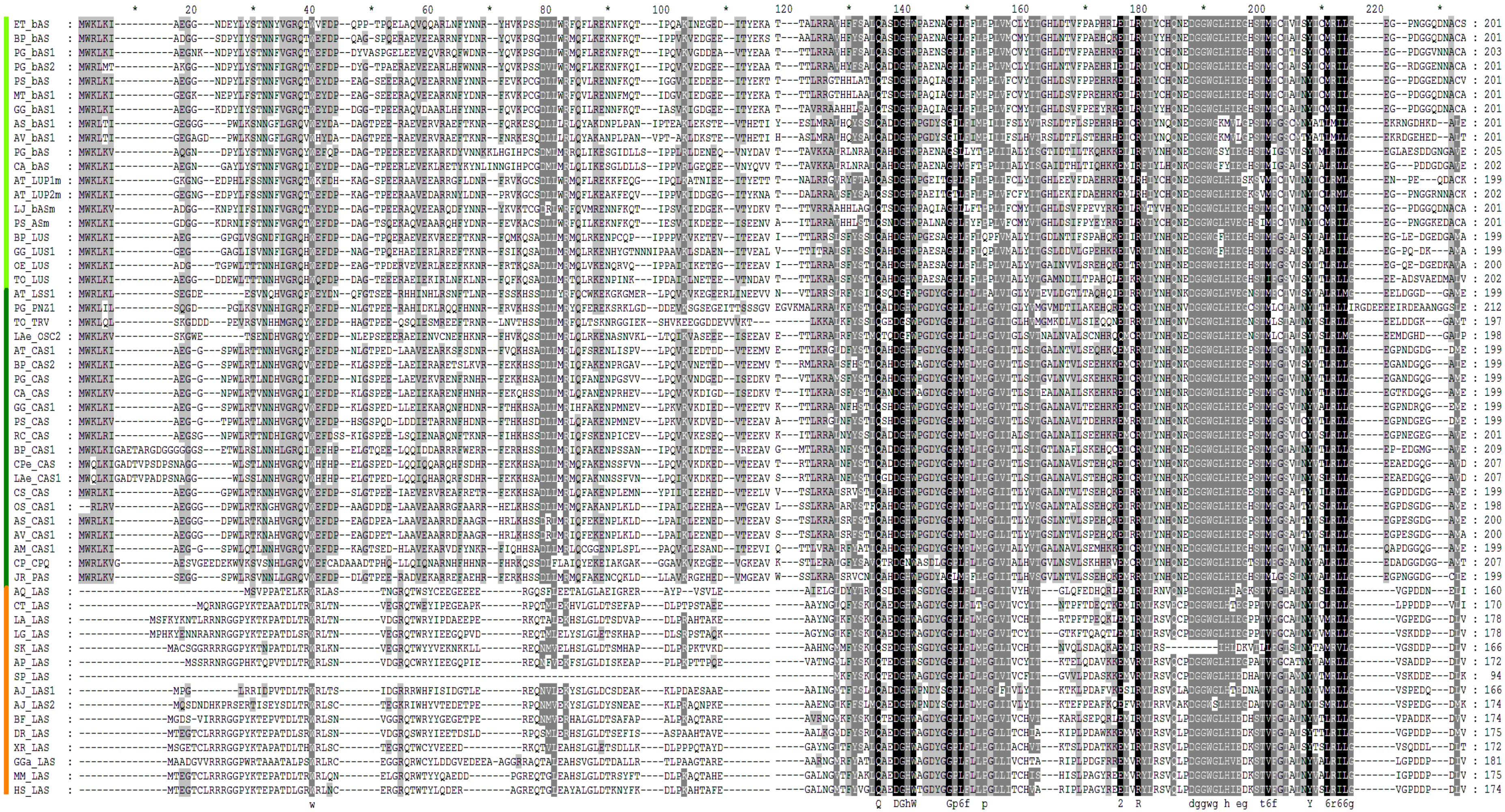
ET_bAS
BP_bAS
PG_bAS1
PG_bAS2
PS_bAS
MT_bAS1
GG_bAS1
AS_bAS1
AV_bAS1
PG_bAS
CA_bAS
AT_LUP1m
AT_LUP2m
LJ_bASm
PS_ASm
BP_LUS
GG_LUS1
OE_LUS
TO_LUS
AT_LSS1
PG_PNZ1
TO_TRV
LAe_OSC2
AT_CAS1
BP_CAS2
PG_CAS
CA_CAS
GG_CAS1
PS_CAS
RC_CAS
BP_CAS1
CPe_CAS
LAe_CAS1
CS_CAS
OS_CAS1
AS_CAS1
AV_CAS1
AM_CAS1
CP_CPQ
JR_PAS
AQ_LAS
CT_LAS
LA_LAS
LG_LAS
SK_LAS
AP_LAS
SP_LAS
AJ_LAS1
AJ_LAS2
BF_LAS
DR_LAS
XR_LAS
GGa_LAS
MM_LAS
HS_LAS

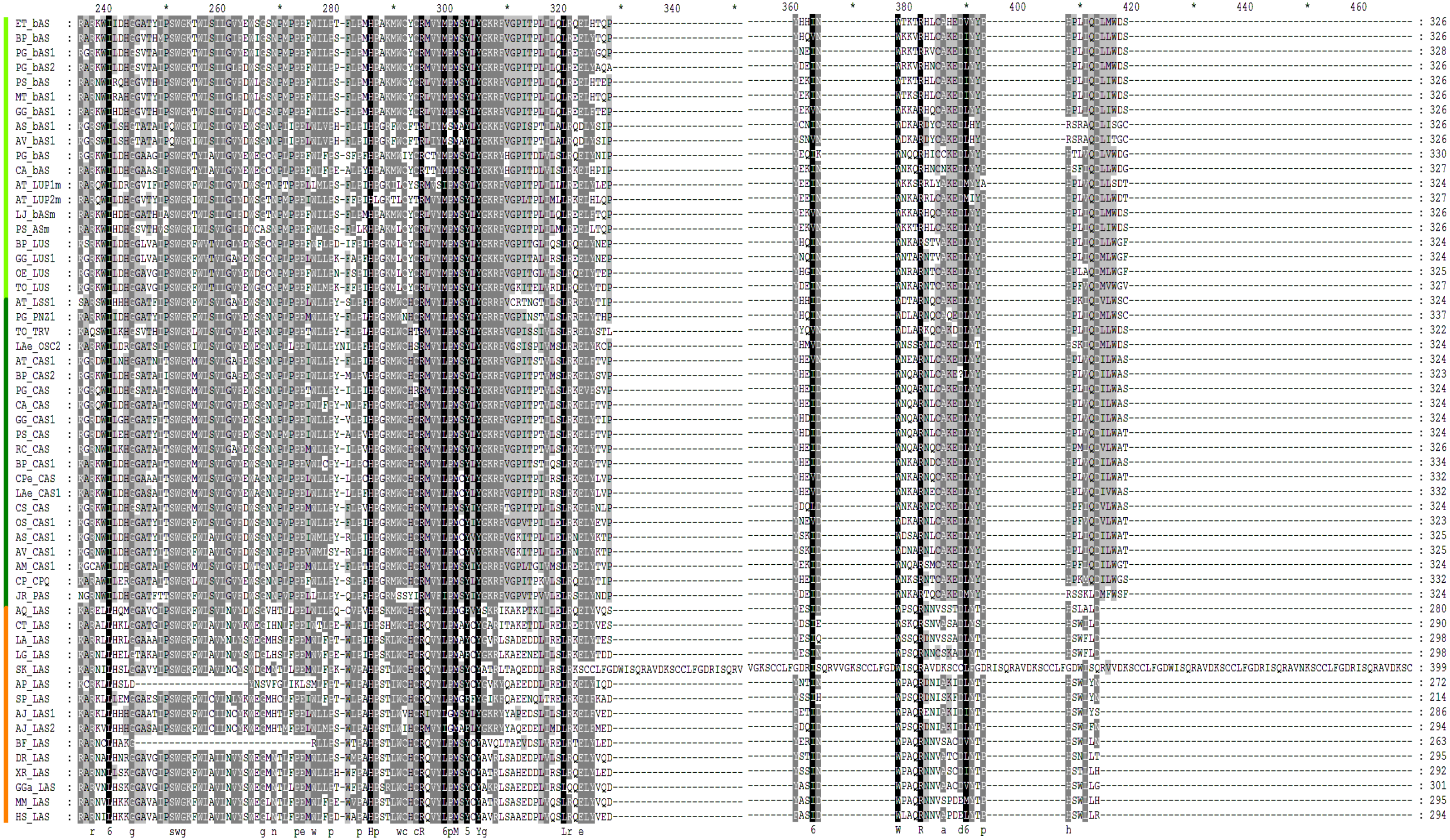

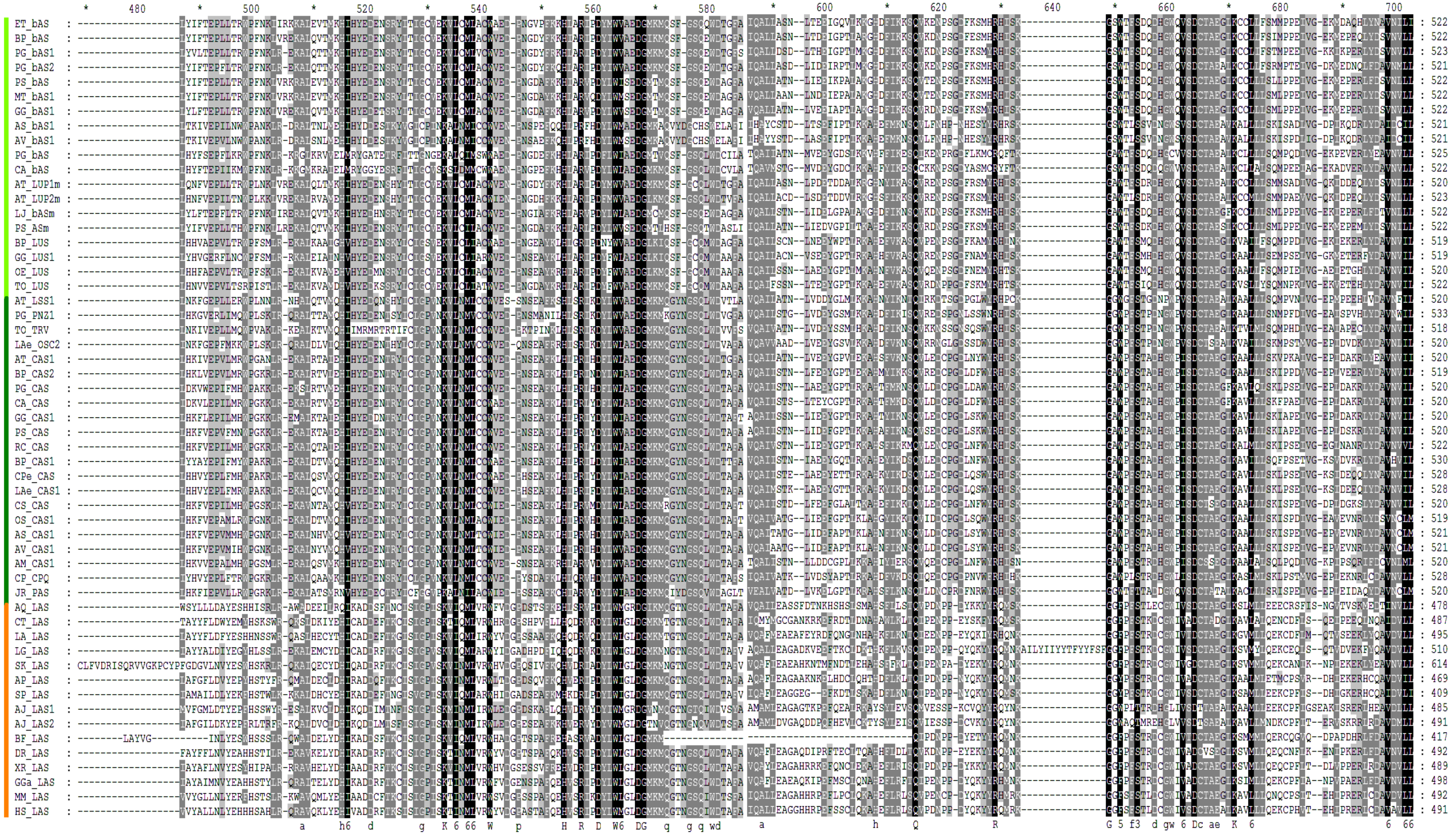

480
500
520
540
560
580
600
620
640
660
680
700
ET_bAS : 522
BP_bAS : 522
PG_bAS1 : 523
PG_bAS2 : 521
PS_bAS : 522
MT_bAS1 : 522
GG_bAS1 : 522
AS_bAS1 : 521
AV_bAS1 : 521
PG_bAS : 525
CA_bAS : 522
AT_LUP1m : 520
AT_LUP2m : 523
LJ_bASm : 522
PS_ASm : 522
BP_LUS : 519
GG_LUS1 : 519
OE_LUS : 520
TO_LUS : 522
AT_LSS1 : 520
PG_PNZ1 : 533
TO_TRV : 518
LAe_OSC2 : 520
AT_CAS1 : 520
BP_CAS2 : 519
PG_CAS : 520
CA_CAS : 520
GG_CAS1 : 520
PS_CAS : 520
RC_CAS : 522
BP_CAS1 : 530
CPe_CAS : 528
LAe_CAS1 : 528
CS_CAS : 520
OS_CAS1 : 519
AS_CAS1 : 521
AV_CAS1 : 521
AM_CAS1 : 520
CP_CPQ : 528
JR_PAS : 520
AQ_LAS : 478
CT_LAS : 487
LA_LAS : 495
LG_LAS : 510
SK_LAS : 614
AP_LAS : 469
SP_LAS : 409
AJ_LAS1 : 485
AJ_LAS2 : 491
BF_LAS : 417
DR_LAS : 492
XR_LAS : 489
GGa_LAS : 498
MM_LAS : 492
HS_LAS : 491

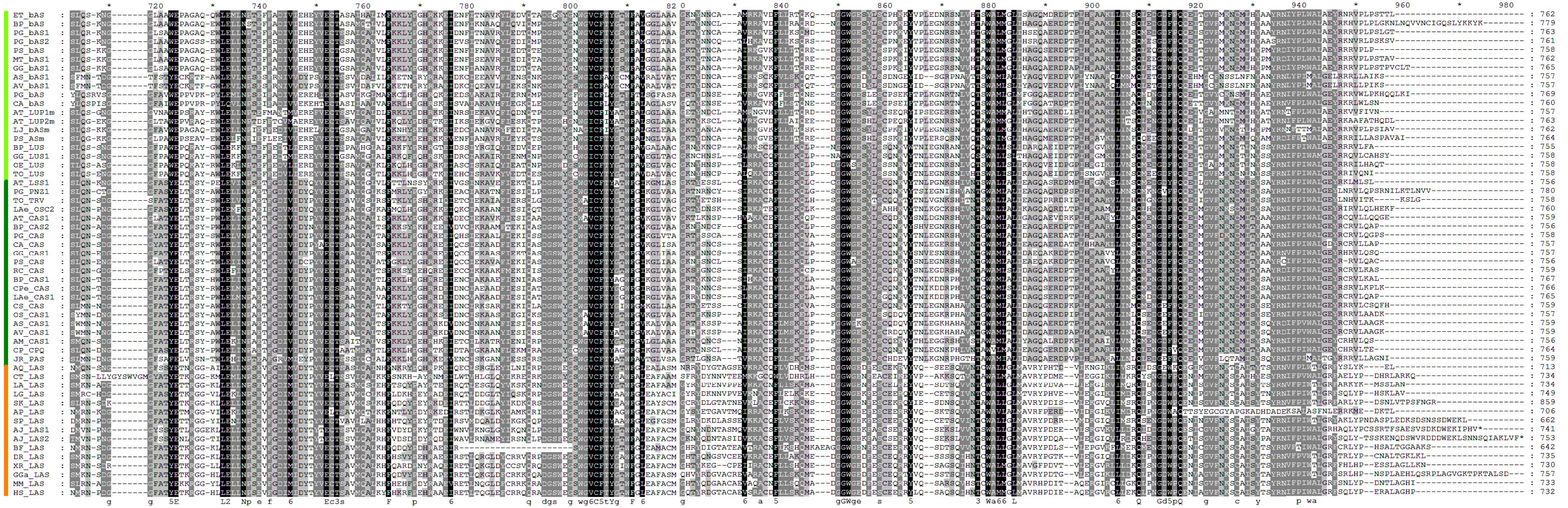

Figure S7 Alignment of oxidosqualene cyclases sites among plants and animals.

Alignment of oxidosqualene cyclases sites among plants and animals was performed by ClustalW [5]. Sequences of the lanosterol synthase (LAS) genes of sea cucumber and other animals were labelled in orange line, compared with cycloartenol synthase (CAS)/cucurbitadienol synthase (CPQ) of plants in dark green and β-amyrin synthase (BAS) of plants in light green. Abbreviations of plants, ET: *Euphorbia tirucalli*, BP: *Betula platyphylla*, PG: *Panax ginseng*, PS:*Pisum sativum*, MT: *Medicago truncatula*, GG:*Glycyrrhiza glabra*, AS: *Avena strigose*, AV: *Avena ventricosa*, CA: *Centella asiatica*, AT: *Arabidopsis thaliana*, LJ: *Lotus japonicas*, OE: *Olea europaea*, TO: *Taraxacum officinale*, LAe: *Luffa aegyptiaca*, RC: *Ricinus communis*, CPe: *Cucurbita pepo* , CS: , OS:, AM:, CP:,JR:. Abbreviations of animals, AQ: *Amphimedon queenslandica*, CT: *Capitella teleta*, LA: *Lingula anatina*, LG: *Lottia gigantea*, SK: *Saccoglossus kowalevskii*, AP: *Acanthaster planci*, SP: *Strongylocentrotus purpuratus*, AJ: *A. japonicus*, BF: *Branchiostoma floridae*, DR: *Drosophila melanogaster*, XR:, GGa: *Gallus gallus*, MM: *Mus musculus*, HS: *Homo sapiens*.

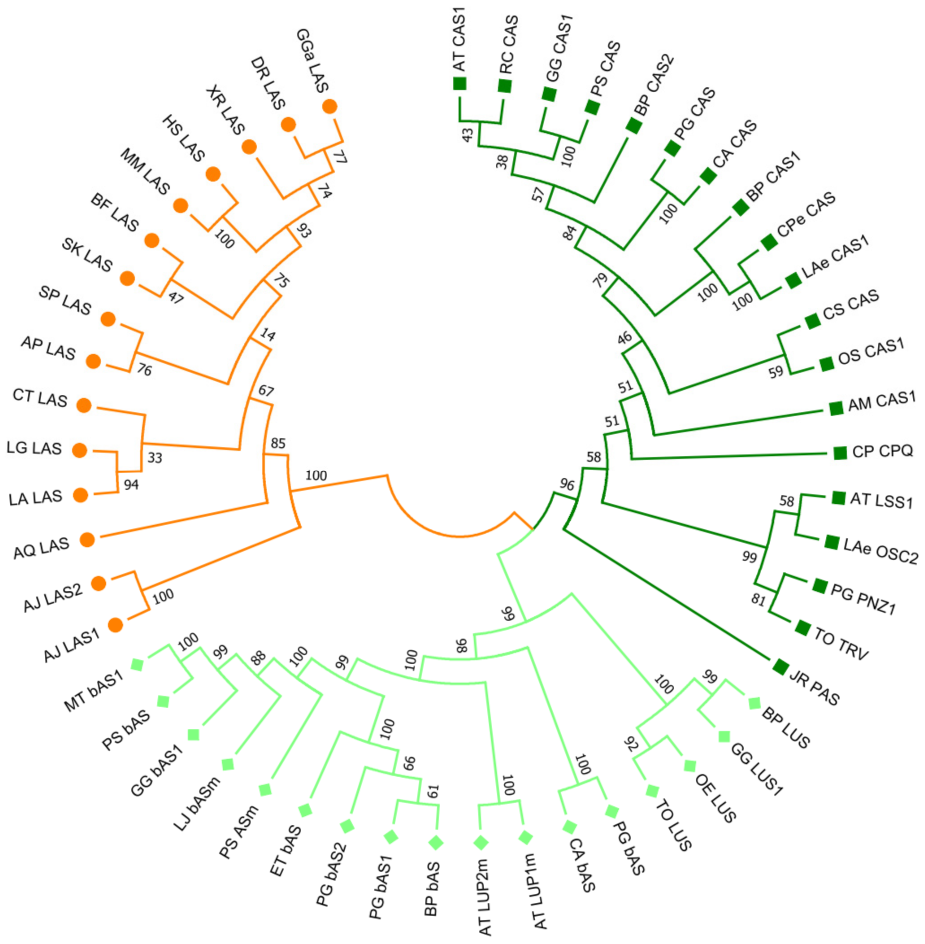

Figure S8 Phylogenetic tree of oxidosqualene cyclases (OSCs).

The evolutionary history was inferred using the Neighbor-Joining method [2]. The evolutionary distances were computed using the p-distance method [3]. Numbers above branches are supporting percentages of 1,000 bootstrap replicates [4]. All positions containing gaps and missing data were eliminated. The sea cucumber LAS genes (AJ_LASs) cluster with other animal LAS genes, suggesting that they belong to the animal-type LAS; while AJ_LASs do not cluster with other echinoderm LASs, possibly due to their fast evolutionary rates. Abbreviations can be referred to those in suppl. figure 7.

.

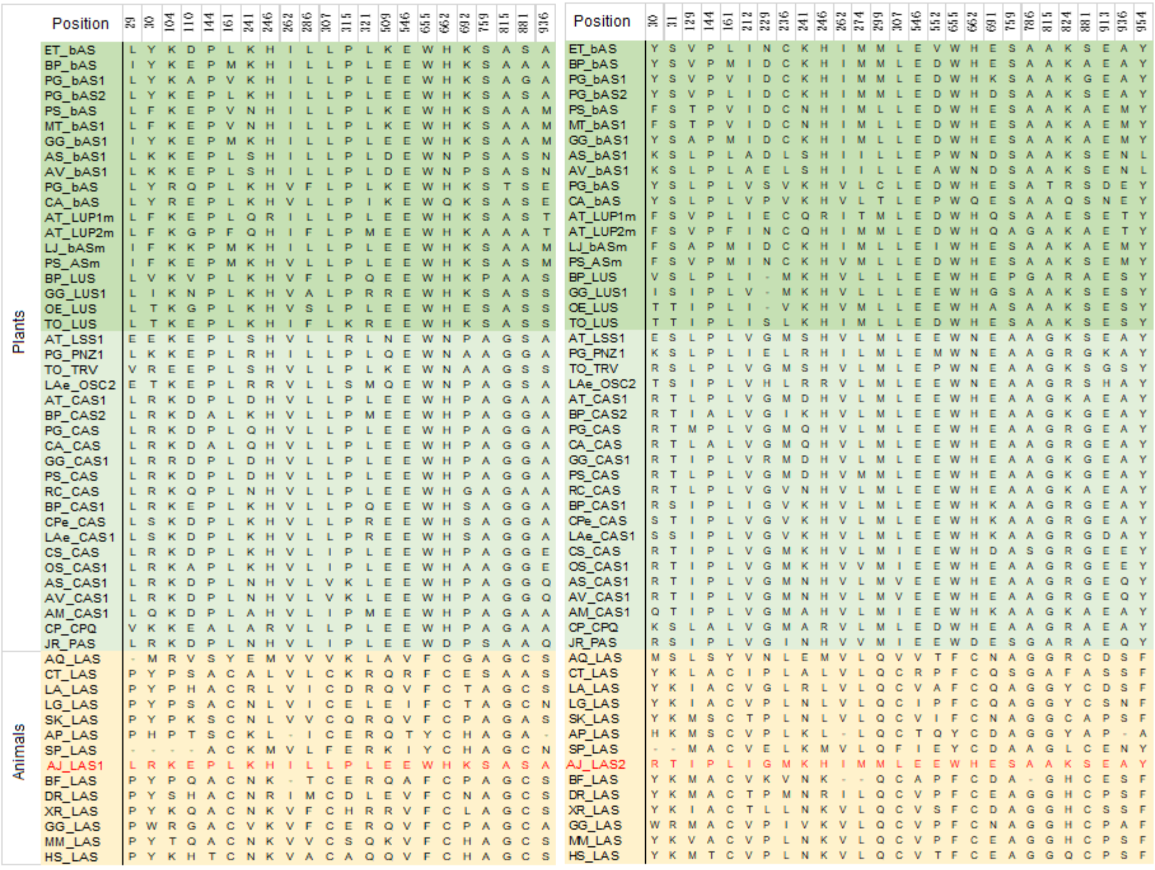

Figure S9 The display of putative plant-like sites of LASs identified in *A. japonicas* and comparison with other animals.

Abbreviations can be referred to those in suppl. figure 7.

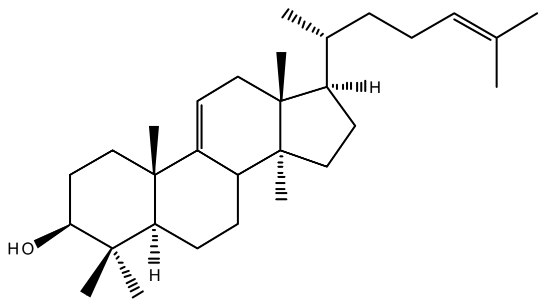 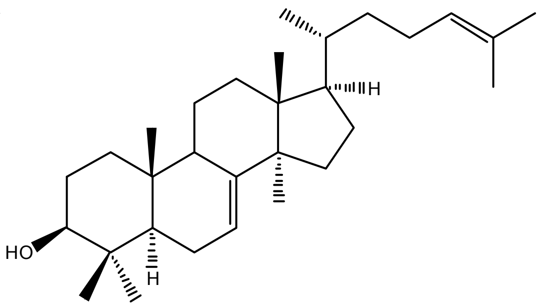

Parkeol Lanosta-7, 24 dienol

Figure S10 Chemical structures of LASs products in *A. japonicus.*

The parkeol came from the products of AJ_LAS1, as well as lanosta-7,24 dienol from AJ_LAS2 in *A. japonicus*.

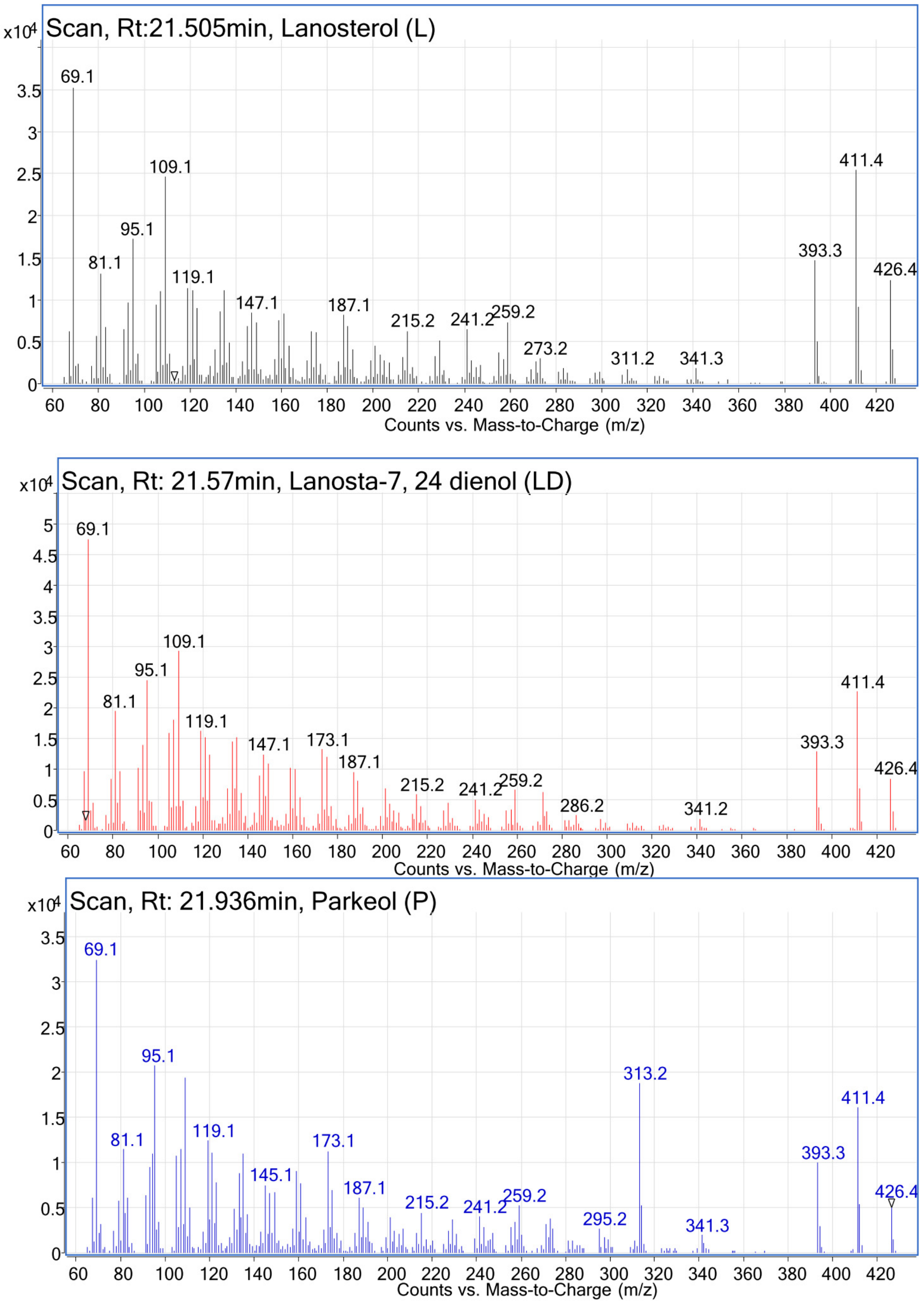

Figure S11 Mass spectra of Lanosterol (L), Lanosta-7, 24 dienol(LD) and Parkeol (P).

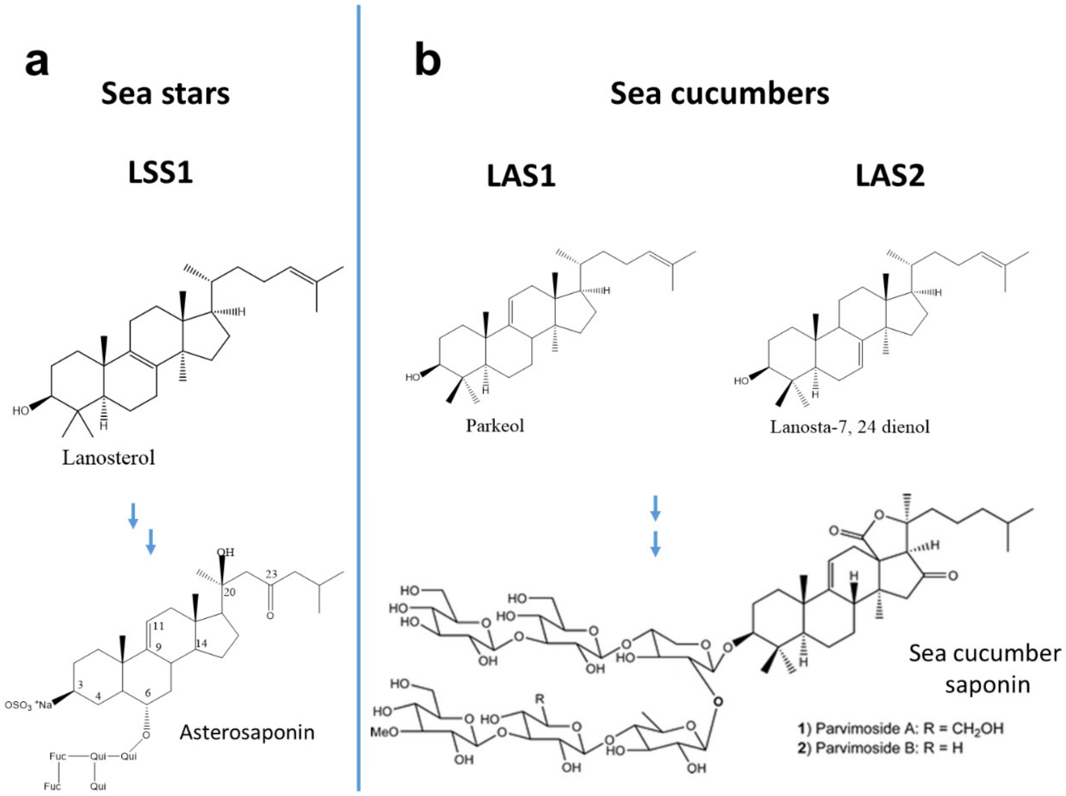

Figure S12 Putative pathway of sterol in sea stars and sea cucumbers.

Putative pathway of sterol precursors leading to sterols and saponins are shown for sea stars and sea cucumbers. Sea stars sapo\nins are steroidal (a) and sea cucumber saponins are triterpenes type (b).

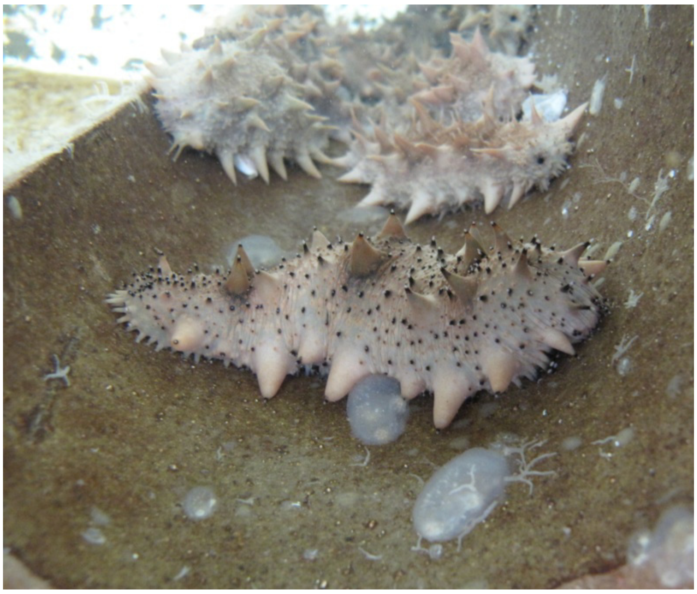

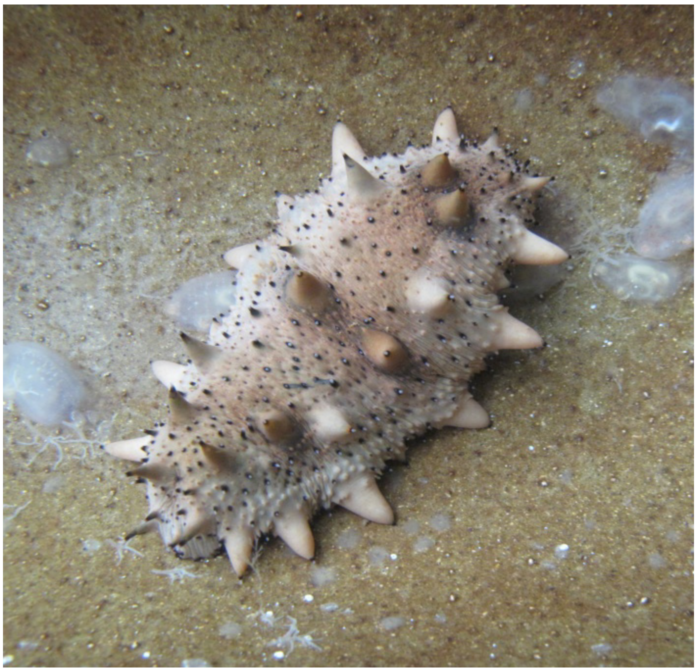

Figure S13 The photographs of sea cucumbers under aestivation.

The sea cucumber *A. japonicus* can enter into aestivation when sea water temperature rises above 25°C with a complete cessation of feeding and locomotion for up to 4 months, and return to active life when temperature decreases below 18°C.

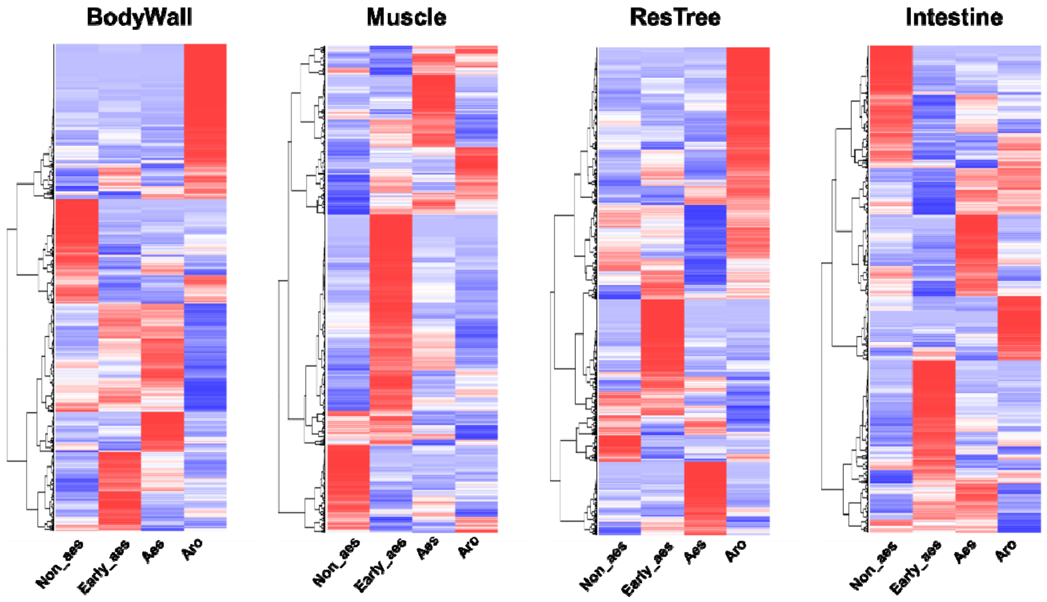

Figure S14 Expression patterns of differential expressed genes in four organs of the sea cucumber *A. japonicus* during aestivation.

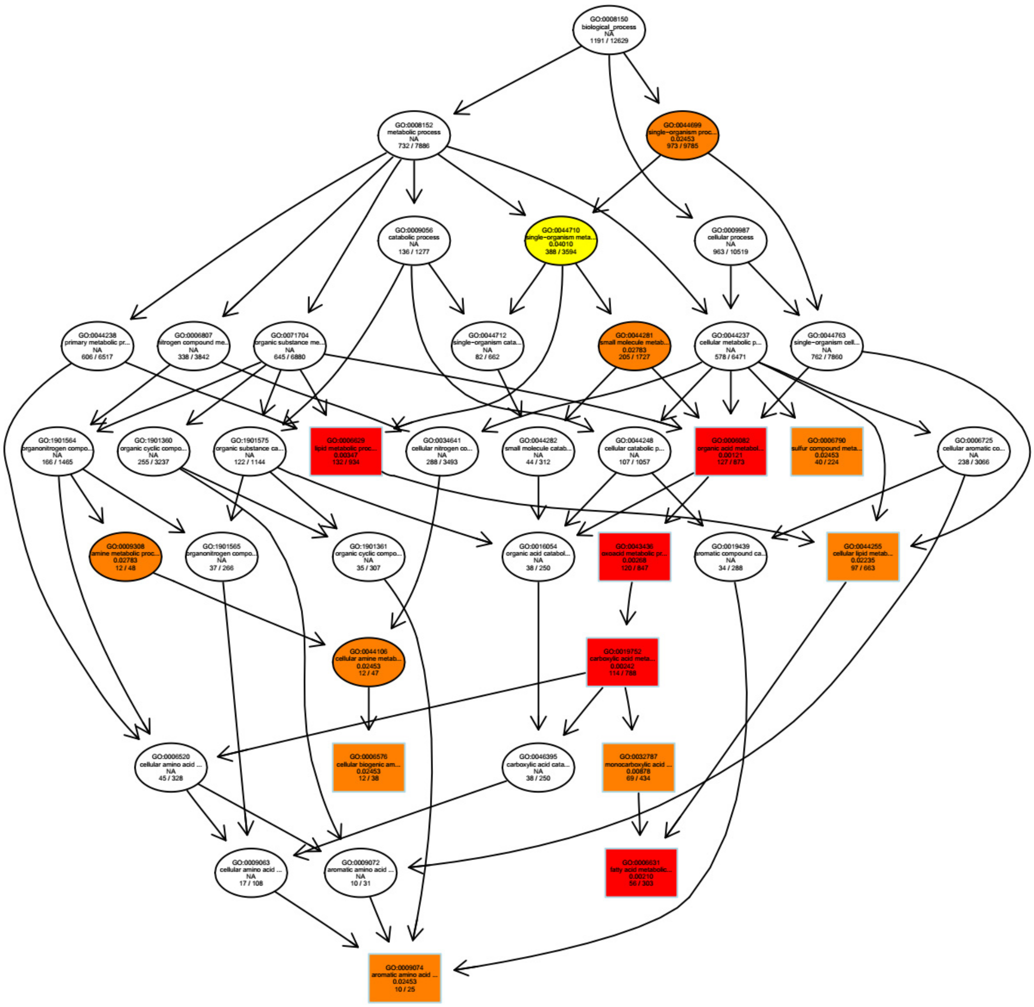

Figure S15 Directed acycline praph (DAG) of GO terms corresponding to biological process by enrichment analysis of DEGs in intestine during sea cucumber aestivation.

GO was performed using the OmicShare tools, a free online platform for data analysis (www.omicshare.com/tools). GO terms that FDR <0.05 are colored from yellow to red, with the increasing of the significance. DGEs are significantly enriched in the GO terms involving in metabolic processes.

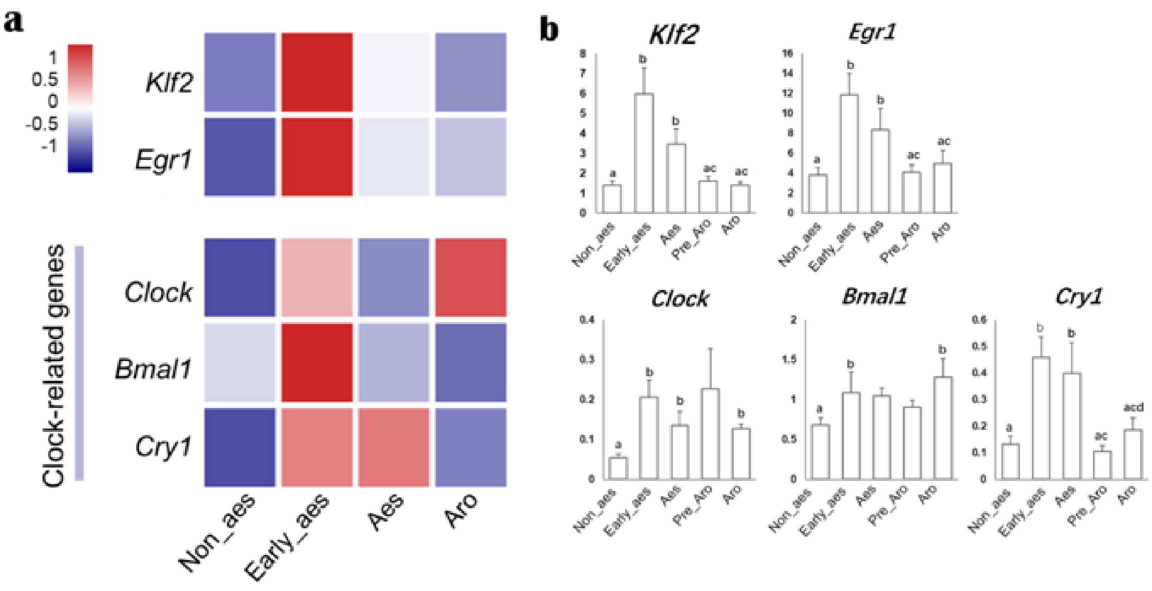

Figure S16 Expression profiles of *Klf2*, *Egr1* and clock-related genes in body wall of *A. japonicus* during aestivation.

(a) Heatmap presentationaccording to RNA-seq experiments.(b) Histogram presentation according to quantitative PCR results. Vertical bars represent the mean ± S.E. (N=12). Different letters indicate significant differences (P<0.05). Aestivation states: non-aestivation (Non_aes), early aestivation (early_ase), deep aestivation (aes), initial arousal from aestivation (pre_aro) and complete arousal from aestivation (aro).

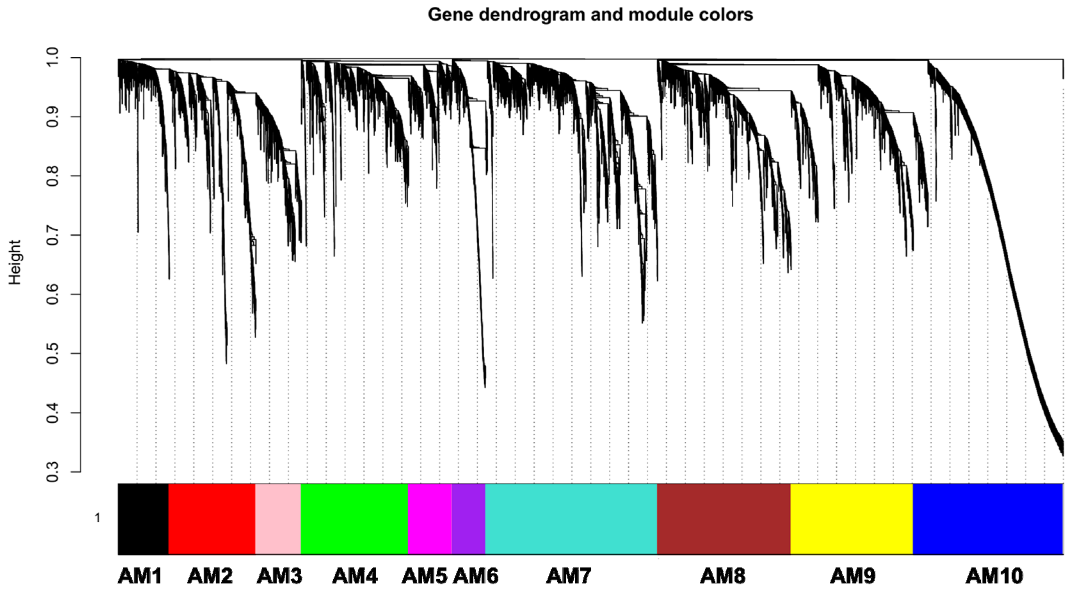

Figure S17 Gene co-expression network of aestivation.

Gene co-expression network constructed using 39 gene expression datasets from four organs (body wall, muscle, respiratory tree and intestine) across four different states during aestivation. Dendrograms are produced by average linkage hierarchical clustering of genes on the basis of topological overlap. Horizontal color bars represent different modules of co-expressed genes.

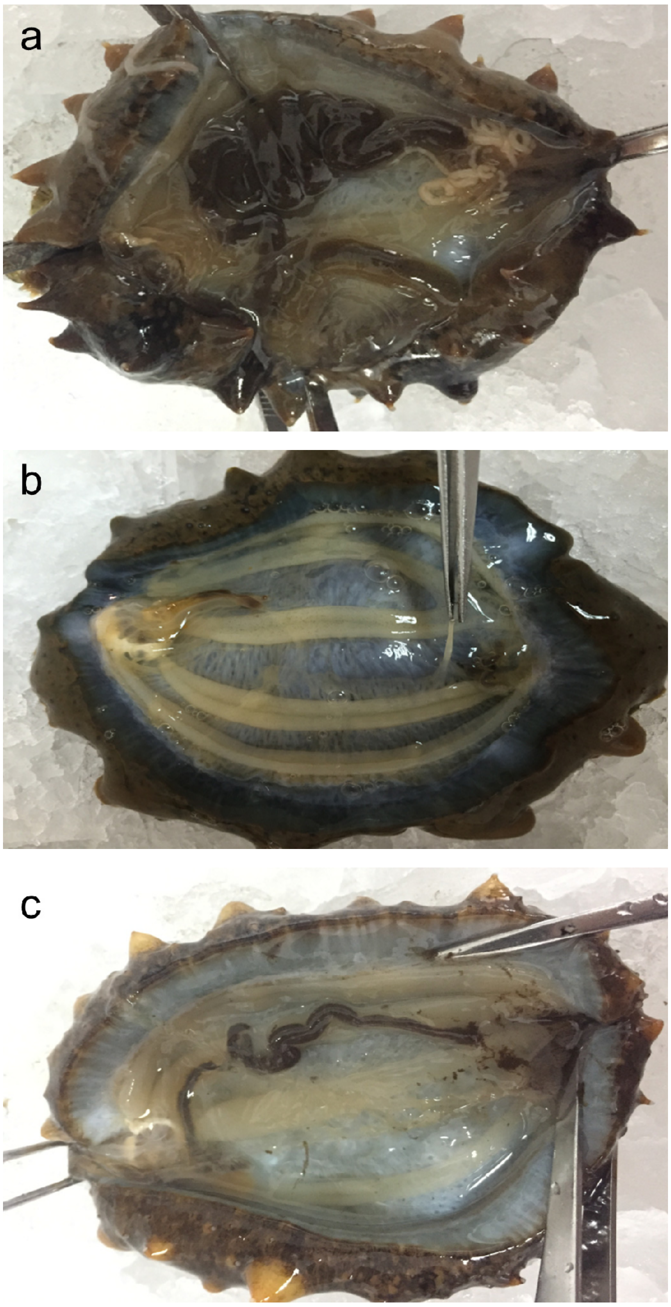

Figure S18 The photograph shows the intestine regeneration of *A. japonicus.*

(a) Normal intestine of sea cucumber. (b) After 10 days' regeneration, the intestine is preliminarily built but without digestive function. Its appearance is gauzy. (c) After 20 days' regeneration, the intestinal wall of sea cucumber is incrassated. Fodder appeared through the intestine, showing the recovered capacity of digestion.

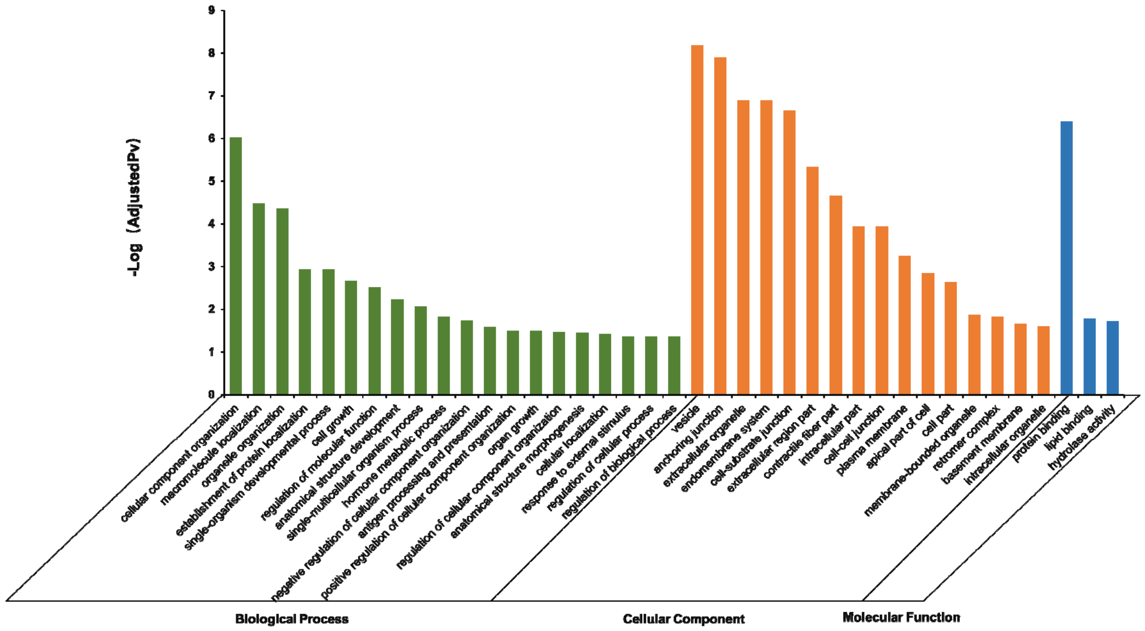

Figure S19 GO enrichment analysis of differential expressed genes during intestine regeneration.

Distribution of the DEGs in each GO term. in the significantly enriched GO terms include cellular component organization, macromolecule localization, cell/organ growth, hormone metabolic process and protein binding.

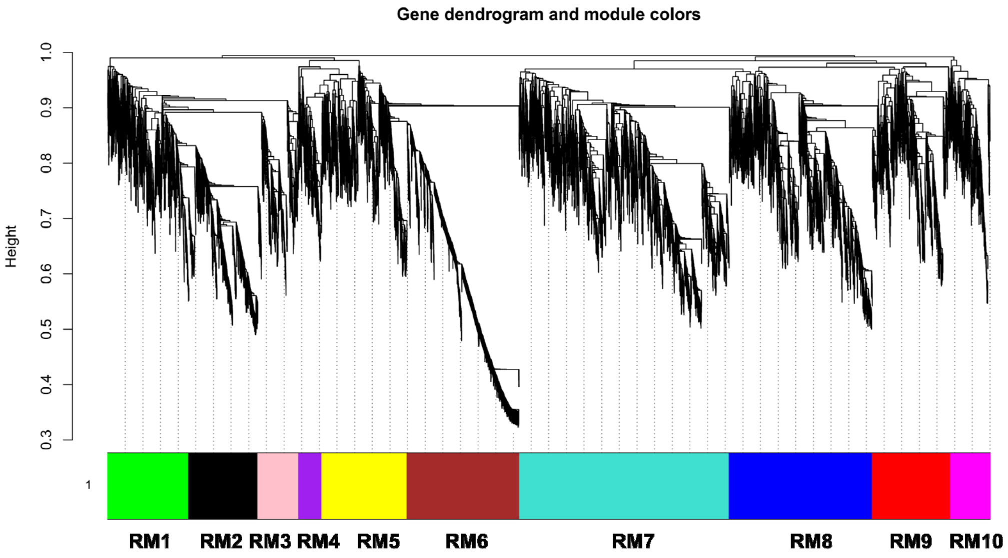

Figure S20 Gene co-expression network of intestine regeneration.

Gene co-expression network assigned co-expressed genes into 10 modules (RM1-RM10). RM7 and RM8 were identified as the regeneration-related modules by enrichment analysis.

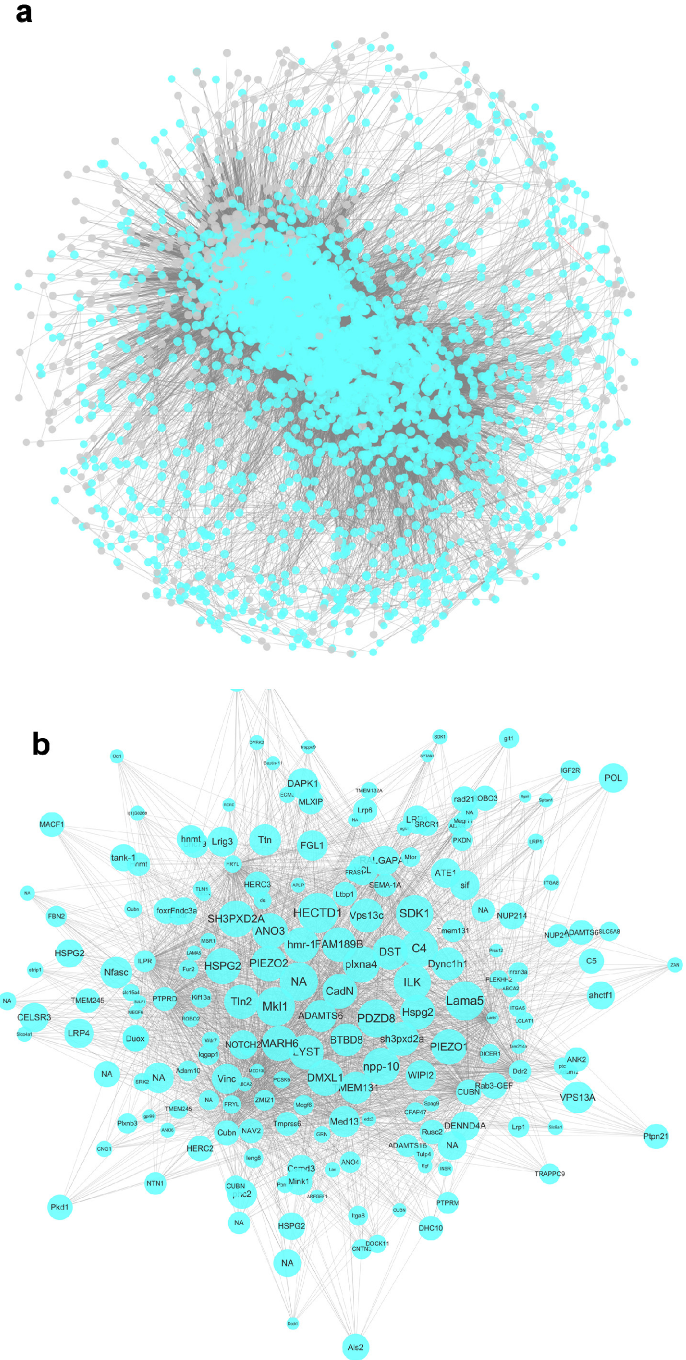

Figure S21 Network visualization of RM7.

(a) 76.6% of genes (turquoise box) in RM7 are differentially expressed during regeneration. (b) Top 200 genes in RM7 with the highest intramodular connectivity are chosen for network display.

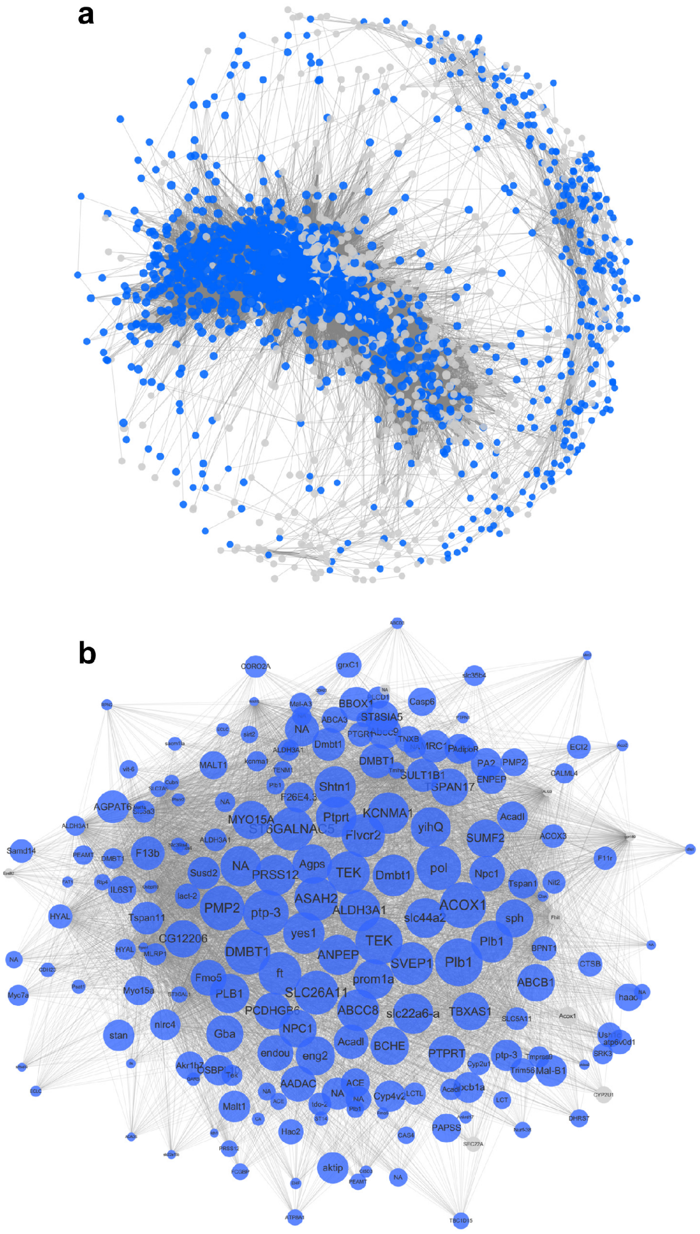

Figure S22 Network visualization of RM8.

(a) 64.6% of genes (blue box) in RM8 are differentially expressed during regeneration. (b) Top 200 genes in RM8 with the highest intramodular connectivity are chosen for network display.

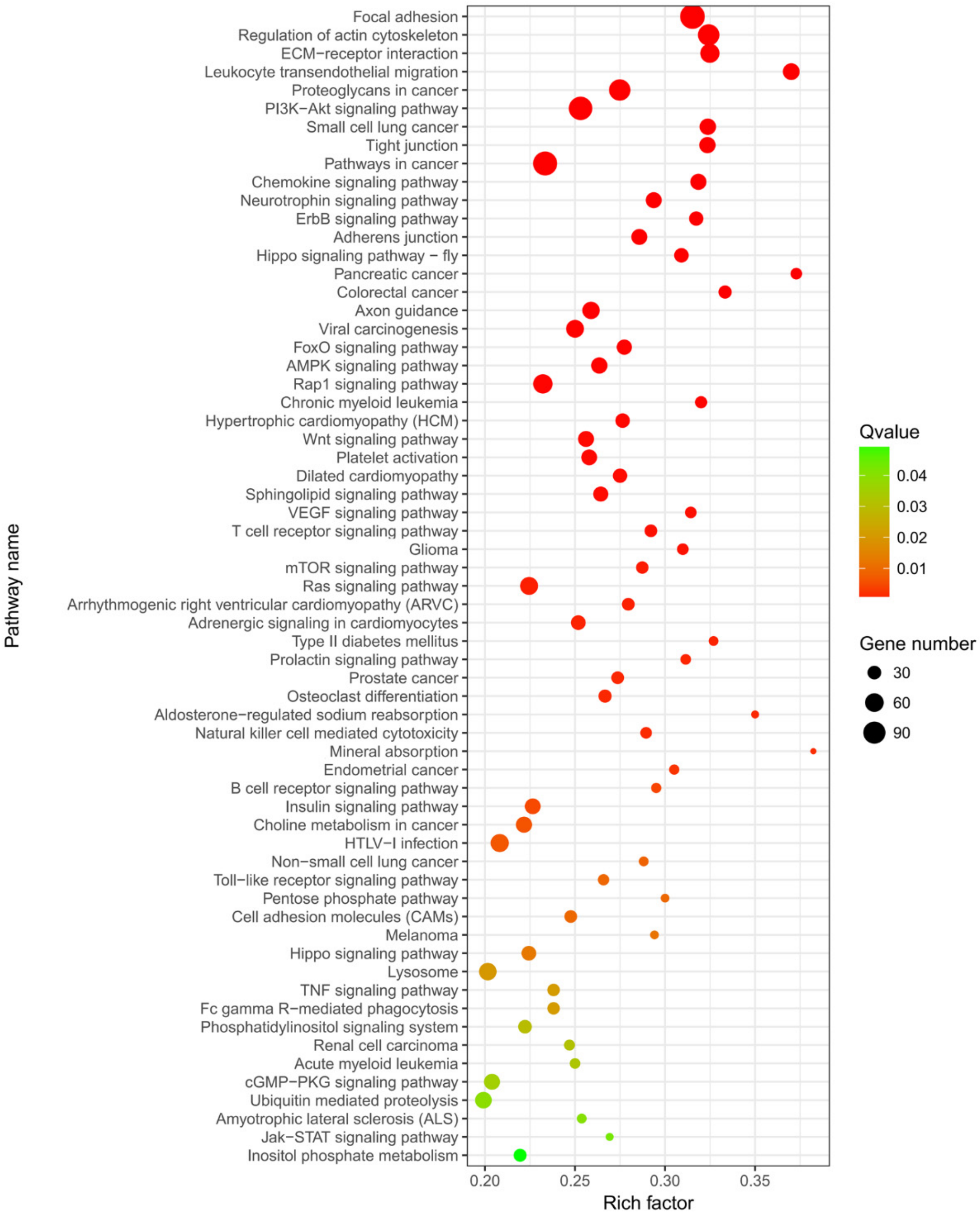

Figure S23 KEGG enrichment of the intestine regeneration-related module RM7.

Pathway enrichment analysis reveals that the a complex molecular regulation with diverse gene pathways involved, including PI3K-Akt, Wnt, Hippo, and mTOR signaling pathways for cell proliferation, differentiation and apoptosis.

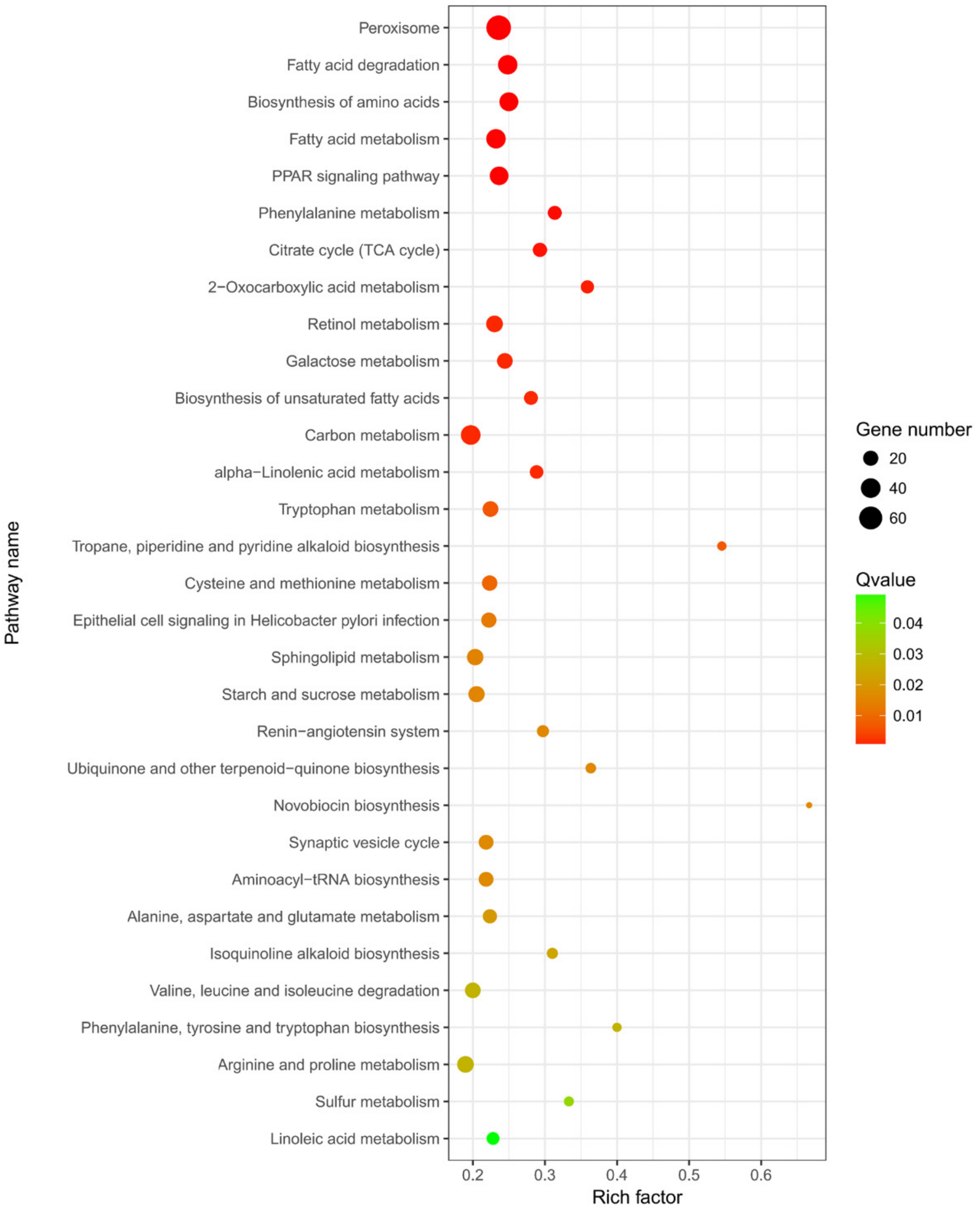

Figure S24 KEGG enrichment of the intestine regeneration-related module RM8.

Genes in RM8 are involved in numerous metabolism pathways such as carbon metabolism, fatty acid metabolism, carbon metabolism and retinol metabolism.

Supplementary Tables.

Table S1 Summary of the *Apostichopus japonicus* genome sequencing data.

Insert Size	Read Length(bp)	Raw Data (G)	Clean Data (G)	Sequence Depth
180 bp	100/100	41.99	41.99	44.1
350 bp	100/100	30.62	30.62	32.2
500 bp	100/100	34.40	34.40	36.1
450 bp	250/250	78.60	70.58	74.1
550 bp	300/300	12.07	10.72	11.3
5 Kbp	100/100	71.55	65.39	69.0
10 Kbp	100/100	21.40	19.54	20.5
15 Kbp	100/100	29.50	27.73	29.1
20 Kbp	100/100	29.21	28.19	29.6
Pac-bio	~9 kb	59.09	23.22	24.4
Total	-	408.43	352.38	370.4

Table S2 Summary statistics of the assembled *A. japonicus* genome.

Assembled genome size	952Mb
Number of chromosomes	22
Sequencing depth	370
Contig N50	45 kb
Scaffold N50	196 kb
ESTs covered by assembly (%)	97.39-99.31
Repeat rate (%)	26.68
Number of protein-coding genes	29,451
Mean gene length	8,918 bp
Mean exon length	202 bp
Mean intron length	1,134 bp
Average exon number per gene	7.4

Table S3 Statistics of the *A. japonicus* genome assembly.

	Contig		Scaffold	
	Size(bp)	Number	Size(bp)	Number
N90	26,742	17,580	62,511	4,784
N80	30,720	14,368	96,361	3,570
N70	35,005	11,563	127,135	2,710
N60	39,793	9,097	160,840	2,045
N50	45,411	6,930	195,518	1,507
Total	919,531,577	21,303	952,279,490	7,286

Table S4 Genome Assembly Evaluation by EST datasets.

Dataset	EST Length	Number	Total Length (bp)	Covered by Assembly		> 90% of Sequence Covered by One Scaffold		> 50% of Sequence Covered by One Scaffold	
						Number	Percent	Number	Percent
Trinity Assemble Unigene	All	1,238,063	776,283,354	1,218,959	98.46%	362,077	29.25%	586,611	47.38%
	> 200	1,238,063	776,283,354	1,205,710	97.39%	358,161	28.93%	581,447	46.96%
	> 500	409,578	527,488,811	404,977	98.88%	97,141	23.72%	196,794	48.05%
	> 1000	175,157	365,968,655	173,942	99.31%	23,783	13.58%	70,114	40.03%
	> 2000	62,671	210,105,631	62,035	98.99%	3,192	5.09%	17,060	27.22%

Table S5 Summary of the linkage map and anchored scaffolds of *A. japonicus.*

See the separate Excel file.

Table S6 Whole-genome gene annotation using different prediction methods.

Annotation method		Gene/EST Number
De novo based	FGENESH	50,794
	GENEMARK	130,797
	Augustus(cegma)	72,637
RNA-Seq based	PASA	221,600 (isoform)
Homolog based	exonerate(14 species)	281,801 (hits)
EVM integration		29,451

Table S7 Summary of gene functional annotations.

	Number	Percent
InterPro	25,982	88.25%
GO	15,770	53.57%
KEGG	16,359	55.57%
Swissprot	26,425	89.76%
Nr	29,269	99.38%
Annotated	29,451	100%
Unanotated	0	0%
Total	29,451	100%

Table S8 Gene characteristics of Echinodermata.

Species	Number	Average Transcript Length (bp)	Average CDS Length (bp)	Average Exons per Gene	Average Exon Length (bp)	Average Intron Length (bp)
Apostichopus japonicus	29,451	8,918	1,324	7.40	202	1,134
Strongylocentrotus purpuratus	27,750	19,568	2,143	8.9	282	1,217
Lytechinus variegatus	28,094	7,863	1,062	5.3	198	1,527
Patiria miniata	29,697	7,257	1,068	6.2	185	1,825
Acanthaster planci	24,747	8,071	1,375	6.8	203	1,161

Table S9 Statistics of the detected repeats in the *A. japonicus* genome and other echinoderms.

Class (Mb)	*Apostichorus japonicus* (952 Mb)		*Strongylocentrotus purpuratus* (991 Mb)		*Patiria miniate* (811 Mb)		*Lytechinus variegatus* (1061 Mb)		*Acanthaster planci* (384 Mb)	
	Lenth (Mb)	Rate (%)	Lenth (Mb)	Rate (%)	Lenth (Mb)	Rate (%)	Lenth (Mb)	Rate (%)	Lenth (Mb)	Rate (%)
Tandem_Repeat	19.34	1.97	40.23	4.06	11.38	1.40	25.31	2.39	3.41	0.89
DNA	34.56	3.52	105.74	10.67	32.74	4.04	211.90	19.97	40.12	10.45
LTR	9.63	0.98	12.04	1.22	8.82	1.09	32.56	3.07	14.82	3.86
LINE	20.33	2.07	37.88	3.82	9.18	1.13	54.94	5.18	18.28	4.76
SINE	5.89	0.60	15.12	1.53	13.88	1.71	32.40	3.05	4.22	1.10
RNA	0.76	0.08	0.16	0.02	0.10	0.01	0.15	0.01	26.17	0.01
Other	12.21	1.24	5.74	0.58	2.89	0.36	48.61	4.58	13.61	3.54
Unclassified (A)	151.26	15.39	135.20	13.64	255.36	31.49	18.69	0.02	5.17	1.35
Total	253.98	26.68	352.11	35.54	334.35	41.23	424.56	38.27	99,76	25.99

Table S10 Summary and comparison among three *A. japonicus* genome assemblies.

	Zhang's version [6]	Jo's version [7]	this assembly
Assembly size (Gb)	0.81	0.66	0.95
Contig N50 (kb)	190	5.5	45
Scaffold N50 (kb)	486	10.5	196
Number of scaffolds	3,281	132,607	7,290
Length of longest scaffold (bp)	2,494,933	131,537	1,156,770
Repeat rate (%)	27.2	27.2	26.6
GC content (%)	36.75	35.92	37.37
Number of protein-coding genes	30,350	21,771	29,451
Mean gene length (bp)	7,787	5,388	8,918
Mean exon length (bp)	193	209	202
Mean intron length (bp)	1,319	1,048	1,134

Table S11 Summary of notochord/stomochord/gill silt-related genes in three *A. japonicus* genome assemblies.

	Gene name	Zhang's version [6]	Jo's version [7]	the assembly of this study
notochord	*brachury*	mRNA.AJAP22596	g39634.t1	AJ283.8
	b-catenin	mRNA.AJAP17491, mRNA.AJAP00402	g65510.t1, g65511.t1	AJ7206.2, AJ5615.3
	hh	mRNA.AJAP09937	g42841.t1, g73769.t1	AJ1894.13
	calumenin	mRNA.AJAP14051, mRNA.AJAP06653, mRNA.AJAP06654	g6107.t1, g72140.t1	AJ3978.13, AJ3978.12
	leprecan	mRNA.AJAP13376	g37661.t1	AJ6909.2
	Admp (BMP2/4)	mRNA.AJAP17205, mRNA.AJAP07010	g67880.t1, g18791.t1	AJ566.3, AJ6686.2
	chordin	mRNA.AJAP13675	g72125.t1	AJ48.9, AJ1206.17
	b4GalT	mRNA.AJAP20115, mRNA.AJAP26309, mRNA.AJAP21792, mRNA.AJAP13686	g6121.t1, g10176.t1, g7929.t1	AJ4262.4, AJ437.4, AJ6677.1, AJ623.1, AJ2927.1, AJ2588.5, AJ7705.2, AJ819.6, AJ515.9
stomochord	*otx*	mRNA.AJAP05566, mRNA.AJAP13361	g30922.t1	AJ1239.3
	dmbx	mRNA.AJAP17215	g52085.t1	AJ593.2
	gsc	mRNA.AJAP01576, mRNA.AJAP21588	Not present	AJ446.4
	dkk	mRNA.AJAP22809	g10775.t1	AJ5557.3
	hex	mRNA.AJAP19023	g6975.t1	AJ4320.7
	nkx2.1	mRNA.AJAP07235	g5132.t1	AJ196.14
gill silt	*nkx2.2*	mRNA.AJAP03077	g25771.t1	AJ168.2
	pax1/9	mRNA.AJAP15401, mRNA.AJAP03074	g39612.t1, g59229.t1	AJ567.4
	mipol1	mRNA.AJAP03073	g31441.t1	AJ1177.4
	foxa1	mRNA.AJAP03072	g46003.t1	AJ1381.2

Table S12 Summary of biomineralization-related genes in three *A. japonicus* genome assemblies.

Genes	Zhang's version [6]	Jo's version [7]	the assembly of this study
can1	mRNA.AJAP03754	g37171.t1	AJ2597.6
colp3a	mRNA.AJAP02383	g72407.t1	AJ416.9
cyp1	mRNA.AJAP01093	g8019.t1	AJ5068.5
cyp2	mRNA.AJAP22588	g68287.t1	AJ240.12
msp130	mRNA.AJAP11747	g52463.t1	AJ4966.2,<br>AJ7701.3
p19L	mRNA.AJAP26626	Not present	AJ1381.2

Table S13 Summary of PSP94-like genes in the assembly generated by this study.

PSP94-like IDs in this assembly	*P* value of this study
AJ1576.2	1.17E-02
AJ1652.1	8.27E-05
AJ1652.4	1.81E-10
AJ1512.1	4.82E-07
AJ1512.2	2.33E-05
AJ1512.3	3.61E-03
AJ1512.4	2.04E-02
AJ1512.5	2.58E-04
AJ1512.6	8.12E-08
AJ1733.1	1

Note: Genes named of AJ1512.1-AJ1512.6 are located in the same scaffold, which is consistent with Zhang et al's report. *P* values indicate the significance of differential gene expression during intestine regeneration. According to our study, most of PSP94-like genes are also differentially expressed during intestine regeneration , supporting their potential roles in intestine regeneration as revealed by Zhang et al. [6].

Table S14 Summary of intestine regeneration-related genes (reported by Zhang et al. [6]) in the assembly of this study.

See the separate Excel file.

Table S15 Number and density of SNPs in eight *A. japonicus* individuals.

	Aj-1	Aj-2	Aj-3	Aj-4	Aj-5	Aj-6	Aj-7	Aj-8
Total	3,612,795	4,084,169	3,968,122	4,015,860	3,956,270	4,200,053	4,012,077	3,964,961
Rate(%)	1.2424	1.2085	1.1515	1.1896	1.1745	1.2318	1.1894	1.1459
Intergenic	2,477,730	2,790,084	2,716,835	2,751,081	2,706,442	2,871,823	2,742,984	2,712,871
Rate(%)	1.2789	1.2327	1.1782	1.2182	1.2013	1.2553	1.2152	1.1639
Gene region	1,135,065	1,294,085	1,251,287	1,264,779	1,249,828	1,328,230	1,269,093	1,252,090
Rate(%)	1.1696	1.1594	1.0977	1.1319	1.1202	1.1840	1.1371	1.1086
Exon	203,100	229,206	222,114	225,471	223,014	235,179	226,545	222,309
Rate(%)	1.0854	1.0753	1.0314	1.0596	1.0493	1.0976	1.0637	1.0331
Intron	931,965	1,064,879	1,029,173	1,039,308	1,026,814	1,093,051	1,042,548	1,029,781
Rate(%)	1.1896	1.1793	1.1131	1.1489	1.1368	1.2044	1.1544	1.1263

Table S16 Summary of the highly polymorphic genes identified from the *A. japonicus* genome.

See the separate Excel file.

Table S17 GO enrichment analysis of the identified highly polymorphic genes.

GO_ID	GO_Term	GO_Class	Pvalue	AdjustedPv
GO:0009056	catabolic process	BP	3.37E-06	0.00013998
GO:0051234	establishment of localization	BP	3.25E-05	0.00094602
GO:0044765	single-organism transport	BP	4.36E-05	0.00105709
GO:0044237	cellular metabolic process	BP	4.90E-05	0.00109661
GO:0009058	biosynthetic process	BP	8.13E-05	0.00164687
GO:0065008	regulation of biological quality	BP	0.000117	0.00199651
GO:0071704	organic substance metabolic process	BP	0.000179	0.00260407
GO:0006807	nitrogen compound metabolic process	BP	0.000547	0.00723783
GO:0007610	behavior	BP	0.000584	0.00738751
GO:0022414	reproductive process	BP	0.001035	0.0108
GO:0044710	single-organism metabolic process	BP	0.001039	0.0108
GO:0044238	primary metabolic process	BP	0.001842	0.01786634
GO:0044703	multi-organism reproductive process	BP	0.002695	0.02529792
GO:0032844	regulation of homeostatic process	BP	0.002785	0.02532177
GO:0009893	positive regulation of metabolic process	BP	0.003444	0.02941336
GO:0032504	multicellular organism reproduction	BP	0.003548	0.02941336
GO:0048609	multicellular organismal reproductive process	BP	0.003726	0.02941336
GO:0044708	single-organism behavior	BP	0.005063	0.03877122
GO:0023056	positive regulation of signaling	BP	0.005948	0.04400163
GO:0044702	single organism reproductive process	BP	0.006048	0.04400163
GO:0045184	establishment of protein localization	BP	0.00636	0.04513715
GO:0006810	transport	BP	0.007157	0.04932387
GO:0043227	membrane-bounded organelle	CC	7.54E-13	2.19E-10
GO:0043229	intracellular organelle	CC	1.44E-06	8.40E-05
GO:0044424	intracellular part	CC	3.08E-06	0.00013998
GO:0016021	integral component of membrane	CC	7.74E-06	0.00028162
GO:0031224	intrinsic component of membrane	CC	1.30E-05	0.00042009
GO:0043230	extracellular organelle	CC	0.000104	0.00188305
GO:0031982	vesicle	CC	0.000131	0.00212354
GO:0012505	endomembrane system	CC	0.000179	0.00260407

GO:0044464	cell part	CC	0.000222	0.00308175
GO:0030529	ribonucleoprotein complex	CC	0.000712	0.00862874
GO:0031090	organelle membrane	CC	0.000967	0.0108
GO:0043233	organelle lumen	CC	0.003092	0.02726633
GO:0044425	membrane part	CC	0.00374	0.02941336
GO:0032420	stereocilium	CC	0.007446	0.04932387
GO:0043234	protein complex	CC	0.007458	0.04932387
GO:0097159	organic cyclic compound binding	MF	3.08E-07	3.83E-05
GO:0043167	ion binding	MF	3.94E-07	3.83E-05
GO:1901363	heterocyclic compound binding	MF	6.03E-07	4.38E-05
GO:0036094	small molecule binding	MF	4.03E-05	0.00105709
GO:0022857	transmembrane transporter activity	MF	8.49E-05	0.00164687
GO:0022892	substrate-specific transporter activity	MF	0.000859	0.00999299
GO:0097367	carbohydrate derivative binding	MF	0.001175	0.01179546

Table S18 The gene representation of canonical animal cholesterol synthesis pathway in *A. japonicus* and other selected animal genomes.

See the separate Excel file.

Table S19 Oligo's used in the study. Underlined sequence indicates pYES2 vector sequence and the sequence in bold corresponds to OSC gene sequence. Middle F and R are overlapping oligos for the two fragments.

Experimental purpose	Oligo Name	Sequence
AJ-LAS1 cloning	AJ-LAS1 Gap.NT-F	ACTACTAGCAGCTGTAATACGACTCACTATAGGGAATATT **ATGCCTGGGCTGAGGAGAATTG**
	AJ-LAS1 middleR	ATAGCCATCGCAGCGTACGAGACGTCCCATATCTGCGTTCCG TTTGTACC
	AJ-LAS1 middleF	GGTACAAACGGAACGCAGATATGGGACGTCTCGTACGCTGC GATGGCTAT
	AJ-LAS1 Gap.CT-R	GAATGTAAGCGTGACATAACTAATTACATGATGCGGCCCT **TTATAACATGGGGGATCTTTTCCCA**
AJ-LAS2 cloning	AJ-LAS2 Gap.NT-F	ACTACTAGCAGCTGTAATACGACTCACTATAGGGAATATT **ATGCAGTCCGATAATGACCACA**
	AJ-LAS2 middleR	ATAGCAGCAAAGGAAGTGTCCCAAACTTGATTACCGTTAGT GCCCTGCAC
	AJ-LAS2 middleF	GTGCAGGGCACTAACGGTAATCAAGTTTGGGACACTTCCTT TGCTGCTAT
	AJ-LAS2 Gap.CT-R	GAATGTAAGCGTGACATAACTAATTACATGATGCGGCCCT **CTAAAATACCAATTTAGCAATCTGC**
Sequencing	AJ-LAS1 middleF	GAATGTCGTATCTCTACGGC
	AJ-LAS2 middleF	ATGAACTGATAATGGACCT
	GAL1.F	AATATACCTCTATACTTTAACGTC
	pYES2.rev	GCGTGAATGTAAGCGTGAC

Table S20 Summary of the read mapping of RNA-seq data from various stages of aestivation.

		Total reads	Total mapped reads	Mapped percentage
Intestine	Non-aes1	16,974,649	13,976,498	82.34%
	Non-aes2	15,798,554	12,971,002	82.10%
	Early-aes1	13,661,149	11,417,448	83.58%
	Early-aes2	16,350,781	13,708,648	83.84%
	Aes1	15,021,969	12,593,986	83.84%
	Aes2	21,301,584	18,019,895	84.59%
	Aes3	23,766,102	20,067,725	84.44%
	Aro1	16,034,078	13,286,104	82.86%
	Aro2	13,345,897	11,226,638	84.12%
	Aro3	20,724,821	17,610,549	84.97%
ResTree	Non-aes1	26,344,952	20,868,096	79.21%
	Non-aes2	21,568,160	17,145,915	79.49%
	Non-aes3	13,690,299	10,872,355	79.42%
	Early-aes1	22,241,524	16,852,828	75.77%
	Early-aes2	28,731,643	22,512,961	78.36%
	Early-aes3	21,893,195	17,284,719	78.95%
	Aes1	22,381,445	18,365,960	82.06%
	Aes2	20,875,229	16,556,455	79.31%
	Aes3	21,890,400	17,718,244	80.94%
	Aro1	19,333,443	15,555,919	80.46%
	Aro2	21,872,984	17,504,817	80.02%
	Aro3	22,638,405	18,391,890	81.24%
BodyWall	Non-aes1	19,778,088	15,703,378	79.40%
	Non-aes2	16,322,903	13,193,106	80.82%
	Non-aes3	17,535,996	13,801,391	78.70%
	Early-aes1	24,645,521	18,785,579	76.22%
	Early-aes2	20,376,260	15,909,496	78.08%
	Aes1	18,351,474	14,354,912	78.22%
	Aes2	21,950,210	17,556,001	79.98%
	Aro1	24,120,910	19,970,096	82.79%
	Aro2	15,409,135	12,406,727	80.52%
Muscle	Non-aes1	23,304,185	19,374,150	83.13%
	Non-aes2	22,162,383	18,307,148	82.61%
	Early-aes1	19,824,535	16,397,419	82.72%
	Early-aes2	22,300,307	18,625,664	83.53%
	Aes1	24,607,023	20,098,998	81.68%
	Aes2	16,935,644	14,478,369	85.49%
	Aro1	18,341,518	13,228,824	72.12%
	Aro2	22,968,284	18,927,828	82.41%

Table S21 Summary of differentially expressed genes (DEGs) in all four organs during aestivation.

See the separate Excel file.

Table S22 GO enrichment analysis of differentially expressed genes in body wall during sea cucumber aestivation.

GO_ID	GO_Term	GO Class	GO level	Pvalue	Adjusted Pv
GO:0001539	ciliary or bacterial-type flagellar motility	BP	4	5.67E-06	0.000799
GO:0007155	cell adhesion	BP	3	1.69E-05	0.001964
GO:0048856	anatomical structure development	BP	3	3.32E-05	0.003441
GO:0032502	developmental process	BP	2	6.03E-05	0.005581
GO:0002685	regulation of leukocyte migration	BP	4	0.000145	0.011868
GO:0044707	single-multicellular organism process	BP	3	0.000207	0.015611
GO:0097529	myeloid leukocyte migration	BP	4	0.000227	0.01682
GO:0003341	cilium movement	BP	6	0.000235	0.01728
GO:0006865	amino acid transport	BP	5	0.000246	0.017941
GO:0046942	carboxylic acid transport	BP	5	0.000287	0.019748
GO:0015711	organic anion transport	BP	5	0.000323	0.021691
GO:0009581	detection of external stimulus	BP	4	0.000328	0.021691
GO:0002688	regulation of leukocyte chemotaxis	BP	5	0.000332	0.021777
GO:0070286	axonemal dynein complex assembly	BP	7	0.000407	0.024657
GO:0030595	leukocyte chemotaxis	BP	4	0.000407	0.024657
GO:0044767	single-organism developmental process	BP	3	0.000424	0.025478
GO:0036159	inner dynein arm assembly	BP	8	0.000575	0.032734
GO:0048771	tissue remodeling	BP	4	0.000603	0.033517
GO:0006811	ion transport	BP	4	0.000603	0.033517
GO:0003008	system process	BP	4	0.000662	0.03536
GO:0032501	multicellular organismal process	BP	2	0.000676	0.03566
GO:0016601	Rac protein signal transduction	BP	8	0.000733	0.037842
GO:0002690	positive regulation of leukocyte chemotaxis	BP	5	0.000735	0.037842
GO:0020028	hemoglobin import	BP	5	0.00081	0.040031
GO:0009182	purine deoxyribonucleoside diphosphate metabolic process	BP	8	0.00081	0.040031
GO:0009216	purine deoxyribonucleoside triphosphate biosynthetic process	BP	8	0.00081	0.040031
GO:0090022	regulation of neutrophil chemotaxis	BP	6	0.00081	0.040031
GO:0030239	myofibril assembly	BP	5	0.00085	0.041534
GO:0060326	cell chemotaxis	BP	5	0.00095	0.045774
GO:0046849	bone remodeling	BP	5	0.001005	0.047774
GO:0005576	extracellular region	CC	2	1.24E-09	3.05E-07
GO:0030286	dynein complex	CC	5	1.83E-09	4.38E-07
GO:0044447	axoneme part	CC	4	2.73E-08	5.73E-06

GO:0005858	axonemal dynein complex	CC	5	3.74E-08	7.65E-06
GO:0005868	cytoplasmic dynein complex	CC	5	3.09E-07	5.79E-05
GO:0044421	extracellular region part	CC	2	1.06E-05	0.001318
GO:0036156	inner dynein arm	CC	6	2.71E-05	0.002914
GO:0005875	microtubule associated complex	CC	4	3.51E-05	0.003554
GO:0032838	cell projection cytoplasm	CC	4	4.01E-05	0.003923
GO:0005930	axoneme	CC	4	8.64E-05	0.007364
GO:0031012	extracellular matrix	CC	2	0.000159	0.012893
GO:0044459	plasma membrane part	CC	3	0.000253	0.018166
GO:0005886	plasma membrane	CC	3	0.000372	0.02304
GO:0005912	adherens junction	CC	4	0.00054	0.030983
GO:0071944	cell periphery	CC	3	0.000662	0.03536
GO:0030670	phagocytic vesicle membrane	CC	6	0.000733	0.037842
GO:0001891	phagocytic cup	CC	4	0.00081	0.040031
GO:0033017	sarcoplasmic reticulum membrane	CC	5	0.000885	0.042996
GO:0003774	motor activity	MF	8	0.000259	0.018402
GO:0003777	microtubule motor activity	MF	9	0.000292	0.019946
GO:0030492	hemoglobin binding	MF	4	0.00081	0.040031
GO:0042954	lipoprotein transporter activity	MF	5	0.00081	0.040031

Table S23 GO enrichment analysis of differentially expressed genes in intestine during sea cucumber aestivation.

GO_ID	GO_Term	GO Class	GO level	Pvalue	Adjusted Pv
GO:0006629	lipid metabolic process	BP	4	4.77E-10	2.76E-07
GO:0006082	organic acid metabolic process	BP	4	8.68E-09	3.24E-06
GO:0001523	retinoid metabolic process	BP	8	2.62E-08	8.27E-06
GO:0019752	carboxylic acid metabolic process	BP	6	2.92E-08	8.80E-06
GO:0006631	fatty acid metabolic process	BP	5	5.58E-08	1.61E-05
GO:0044255	cellular lipid metabolic process	BP	4	7.66E-08	1.97E-05
GO:0043436	oxoacid metabolic process	BP	5	1.13E-07	2.81E-05
GO:0032787	monocarboxylic acid metabolic process	BP	7	5.76E-07	0.000105
GO:0034754	cellular hormone metabolic process	BP	4	6.36E-06	0.000958
GO:0010876	lipid localization	BP	4	8.91E-06	0.001287
GO:0001676	long-chain fatty acid metabolic process	BP	6	1.72E-05	0.00225
GO:0015908	fatty acid transport	BP	5	1.91E-05	0.002413
GO:0033559	unsaturated fatty acid metabolic process	BP	6	2.05E-05	0.002533
GO:0042572	retinol metabolic process	BP	5	2.76E-05	0.003303
GO:0006869	lipid transport	BP	4	4.67E-05	0.005491
GO:0009074	aromatic amino acid family catabolic process	BP	6	6.43E-05	0.006968
GO:0044281	small molecule metabolic process	BP	4	0.000118	0.012074
GO:0006720	isoprenoid metabolic process	BP	5	0.000201	0.019131
GO:0015718	monocarboxylic acid transport	BP	6	0.000246	0.023019
GO:0070266	necroptotic process	BP	7	0.000289	0.025645
GO:0015909	long-chain fatty acid transport	BP	6	0.000289	0.025645
GO:0006790	sulfur compound metabolic process	BP	4	0.000324	0.028082
GO:0097300	programmed necrotic cell death	BP	6	0.00034	0.028725
GO:0042445	hormone metabolic process	BP	3	0.000418	0.033342
GO:0016115	terpenoid catabolic process	BP	7	0.000429	0.033781
GO:0045834	positive regulation of lipid metabolic process	BP	4	0.000521	0.03966
GO:0009066	aspartate family amino acid metabolic process	BP	6	0.000578	0.043084
GO:0044710	single-organism metabolic process	BP	3	0.000693	0.049521
GO:0031526	brush border membrane	CC	5	0.000199	0.019131
GO:0004622	lysophospholipase activity	MF	6	2.16E-06	0.000356
GO:0004620	phospholipase activity	MF	6	2.78E-06	0.000448

GO:0016491	oxidoreductase activity	MF	3	1.39E-05	0.001893
GO:0016298	lipase activity	MF	5	1.86E-05	0.002382
GO:0016725	oxidoreductase activity, acting on CH or CH2 groups	MF	4	0.000104	0.011077
GO:0016717	oxidoreductase activity, acting on paired donors, with oxidation of a pair of donors resulting in the reduction of molecular oxygen to two molecules of water	MF	5	0.00037	0.029862
GO:0050253	retinyl-palmitate esterase activity	MF	6	0.000502	0.038695

Table S24 GO enrichment analysis of differentially expressed genes in muscle during sea cucumber aestivation.

GO_ID	GO_Term	GO Class	GO level	Pvalue	Adjusted Pv
GO:0030317	sperm motility	BP	4	4.33E-07	0.000583
GO:0070286	axonemal dynein complex assembly	BP	7	5.13E-05	0.025894
GO:0021831	embryonic olfactory bulb interneuron precursor migration	BP	6	9.52E-05	0.03663
GO:0006956	complement activation	BP	4	0.000133	0.044863
GO:0043277	apoptotic cell clearance	BP	5	0.000176	0.047431
GO:0030286	dynein complex	CC	5	2.26E-09	1.83E-05
GO:0005858	axonemal dynein complex	CC	5	7.85E-09	3.17E-05
GO:0005868	cytoplasmic dynein complex	CC	5	6.05E-08	0.000122
GO:0031012	extracellular matrix	CC	2	2.17E-07	0.00035
GO:0005578	proteinaceous extracellular matrix	CC	3	1.04E-06	0.001206
GO:0005576	extracellular region	CC	2	4.37E-06	0.004412
GO:0005605	basal lamina	CC	3	7.48E-06	0.006717
GO:0044420	extracellular matrix part	CC	2	2.55E-05	0.019668
GO:0036157	outer dynein arm	CC	6	3.16E-05	0.019668
GO:0042575	DNA polymerase complex	CC	4	3.16E-05	0.019668
GO:0003777	microtubule motor activity	MF	9	5.07E-05	0.025894

Table S25 GO enrichment analysis of differentially expressed genes in respiratory tree during sea cucumber aestivation.

GO_ID	GO_Term	GO Class	GO level	Pvalue	AdjustedPv
GO:0042572	retinol metabolic process	BP	5	1.36E-05	0.001417
GO:0050778	positive regulation of immune response	BP	4	1.82E-05	0.001825
GO:0002253	activation of immune response	BP	3	1.98E-05	0.001964
GO:0001867	complement activation, lectin pathway	BP	5	3.39E-05	0.00309
GO:0042445	hormone metabolic process	BP	3	7.03E-05	0.005752
GO:0006959	humoral immune response	BP	4	8.82E-05	0.006999
GO:0002221	pattern recognition receptor signaling pathway	BP	6	0.000129	0.009326
GO:0006956	complement activation	BP	4	0.000234	0.016292
GO:0030261	chromosome condensation	BP	4	0.000317	0.021133
GO:0020028	hemoglobin import	BP	5	0.000385	0.024627
GO:0097070	ductus arteriosus closure	BP	6	0.000385	0.024627
GO:0044699	single-organism process	BP	2	0.000482	0.029659
GO:0048844	artery morphogenesis	BP	5	0.000781	0.044589
GO:0060840	artery development	BP	5	0.000843	0.047158
GO:0002224	toll-like receptor signaling pathway	BP	7	0.000888	0.048976
GO:0031526	brush border membrane	CC	5	1.52E-07	2.81E-05
GO:0045177	apical part of cell	CC	3	5.25E-07	7.72E-05
GO:0016324	apical plasma membrane	CC	4	6.66E-07	9.44E-05
GO:0005903	brush border	CC	4	1.12E-05	0.001222
GO:0009986	cell surface	CC	3	7.25E-05	0.005871
GO:0098590	plasma membrane region	CC	4	7.50E-05	0.006012
GO:0005615	extracellular space	CC	3	0.00012	0.008922
GO:0005886	plasma membrane	CC	3	0.000624	0.037256
GO:0050253	retinyl-palmitate esterase activity	MF	6	2.52E-05	0.002406
GO:0004623	phospholipase A2 activity	MF	6	0.000103	0.007993
GO:0030492	hemoglobin binding	MF	4	0.000385	0.024627
GO:0042954	lipoprotein transporter activity	MF	5	0.000385	0.024627
GO:0016491	oxidoreductase activity	MF	3	0.000396	0.025123
GO:0004622	lysophospholipase activity	MF	6	0.000509	0.031104
GO:0004872	receptor activity	MF	2	0.000805	0.045341

Table S26 Primer sequences used for quantitative PCR validation.

Gene symbol	Primer sequences-F (5′–3′)	Primer sequences-R (5′–3′)
Klf2	CCACCAGCCTTTACTCAGCAT	GTGTTTGTGCAACCAATCCCT
Egr1	CCAAGCTCCTCGACACCAGT	GGACTTCCACCCACGATAGACT
Clock	ACTCATCGCGTCGTCAGCTA	GAAATCTCGGACAGGCAACC
Bmal1	GGTCAAGTCTGATGTGGCTCC	TTGGTCCTCTGTCTTCACGATG
Cry1	TCAACTCCCGTCTGTTTCTGG	CGGTTCGCAGTCTTCCTCA
Dpolm	ACCTGACAGTGACATGCGACC	AGCTCTGTCTGATTTACTTCGAGAT
Grb2	ATCTTTCACATATTGCGAGCCAG	ATGACCATTCCGATGCCCTAA

Table S27 Module enrichment analysis of overrepresented aestivation-related genes.

color	BodyWall-FDR	Muscle-FDR	Intestine-FDR	ResTree-FDR
AM7	5.58E-176	0.003894	1	9.21E-25
AM2	1	0.998946	4.96E-106	1.80E-45
AM3	1	0.998946	5.40E-11	2.19E-50
AM6	1.05E-83	0.998946	1	1
AM1	1	0.998946	1.52E-17	1
AM4	1	0.201392	0.124178	5.03E-07
AM8	1	0.003894	1	1
AM5	1	0.998946	1	1
AM9	1	0.998946	1	1
AM10	1	0.998946	1	1

Table S28 Summary of transcription factors in the AM7 network.

geneID	Kwithin	UNI_name	UNI_Evalue	Number*
AJ983.7	102.155384	EGR1_XENTR	7E-59	4
AJ2180.2	62.9801003	KLF2_HUMAN	8E-49	4
AJ537.29	23.202925	NFE2_HUMAN	3E-44	4
AJ4515.2	12.6726782	AKR_CHICK	1E-24	4
AJ130.28	83.308794	CR3LA_DANRE	6E-29	3
AJ851.6	79.7816558	RREB1_CHICK	5E-49	3
AJ4965.4	78.6657639	ATF6A_HUMAN	1E-38	3
AJ3840.7	46.3973246	PEBB_HUMAN	9E-65	3
AJ594.9	34.309651	ARID2_HUMAN	2E-38	3
AJ557.11	33.5340241	CR3L3_RAT	3E-28	3
AJ1073.2	32.6402878	ZMIZ1_HUMAN	4E-173	3
AJ46.33	30.2624709	SMBT2_HUMAN	4E-125	3
AJ870.41	2.62689747	TYW1_DANRE	8E-46	3
AJ285.14	139.086501	EVI1_HUMAN	2E-62	2
AJ894.16	91.0378344	SOX14_DANRE	4E-57	2
AJ76.23	86.057176	E75_CHOFU	5E-37	2
AJ4977.11	85.0118334	ALX4_HUMAN	8E-36	2
AJ471.13	74.1701169	HES1B_XENLA	6E-27	2
AJ5604.1	60.942267	ARID2_HUMAN	2E-29	2
AJ6629.1	44.5984609	SMRC2_HUMAN	2E-165	2
AJ1308.10	41.429722	ARI1A_HUMAN	4E-74	2
AJ1747.17	41.1519435	FOXN3_MOUSE	7E-34	2
AJ802.14	35.0149765	NFIL3_HUMAN	4E-20	2
AJ1846.6	34.8873875	ZN236_HUMAN	1E-82	2
AJ569.14	31.3568718	MRF_HUMAN	3E-62	2
AJ887.8	28.752419	SMAD1_BOVIN	0	2
AJ4156.3	25.0529411	PIAS3_RAT	6E-107	2
AJ1911.1	21.164901	EHF_BOVIN	1E-42	2
AJ2469.6	18.7579821	KDM5A_HUMAN	2E-160	2
AJ2422.1	16.5918688	MLXIP_HUMAN	9E-41	2
AJ3658.3	16.258889	NFAT5_RAT	5E-75	2
AJ2443.1	12.4563674	MLXPL_HUMAN	8E-40	2
AJ5725.1	11.3284555	MLXPL_HUMAN	1E-54	2
AJ2372.8	11.117842	LMBL1_CHICK	3E-176	2
AJ1095.19	8.35102751	ATF2_CHICK	8E-38	2
AJ1124.12	5.07291809	GLAS_DROVI	1E-42	2
AJ4517.4	181.142822	NR5A2_HUMAN	3E-47	1
AJ1501.4	165.650824	GFI1_RAT	5E-92	1
AJ1108.7	136.348966	NR1D2_MOUSE	6E-35	1
AJ5709.2	123.255669	SP5_MOUSE	2E-55	1
AJ1430.4	110.398337	DIMM_DROME	5E-26	1

AJ865.5	107.848433	SNAI2_XENLA	2E-75	1
AJ1393.7	98.9418945	PTF1A_DANRE	1E-56	1
AJ4301.2	90.6951866	BARH2_HUMAN	3E-33	1
AJ1359.13	78.9661132	RXRAA_DANRE	7E-61	1
AJ4320.7	77.5490054	HHEX_PATMI	8E-34	1
AJ3034.11	66.7440243	CAN5_HUMAN	0	1
AJ673.21	33.7768205	NFKB1_HUMAN	5E-70	1
AJ1171.8	30.3498158	SHR2_STRPU	1E-69	1
AJ3106.4	28.6721018	CREM_CANFA	8E-59	1
AJ1262.8	27.5227391	SIX1B_DANRE	1E-121	1
AJ2539.7	25.0415104	KDM5D_HUMAN	0	1
AJ4035.19	23.8302391	PB1_HUMAN	0	1
AJ1603.1	20.2605683	MLXPL_RAT	2E-25	1
AJ1132.6	18.8888162	FOXO_DROAN	2E-63	1
AJ2257.2	15.1754867	SHR2_STRPU	1E-167	1
AJ2257.3	11.9657272	SHR2_STRPU	4E-68	1
AJ718.19	10.4299196	KLF11_HUMAN	3E-63	1
AJ1247.1	163.244814	PO4F3_MOUSE	6E-93	0
AJ1961.1	151.145909	ZNF79_HUMAN	1E-63	0
AJ2365.1	86.4550165	RARB_COTJA	1E-52	0
AJ6525.1	36.0726915	MTA1_HUMAN	0	0
AJ1939.3	26.9123664	ZEB2_MOUSE	3E-44	0
AJ845.3	24.8692768	MYT1L_MOUSE	5E-46	0
AJ4529.9	23.2807463	ZNF14_PONAB	5E-93	0
AJ240.16	20.5370093	RERE_HUMAN	5E-166	0
AJ876.9	18.600375	MIER1_XENLA	3E-70	0
AJ379.9	17.889244	ZN271_PONAB	8E-124	0
AJ4235.1	11.427742	XBP1_MOUSE	9E-27	0
AJ2849.4	11.4038415	ZN454_HUMAN	1E-32	0
AJ2880.2	10.0449635	SUH_BOVIN	3E-176	0
AJ5242.5	9.40795982	HMBX1_HUMAN	7E-24	0
AJ161.16	7.67531538	ZFP2_HUMAN	5E-71	0
AJ2283.9	7.16939206	ZBED4_HUMAN	5E-30	0
AJ851.5	6.85506123	ZN341_HUMAN	2E-77	0
AJ195.18	6.75262249	USF2_RAT	9E-75	0
AJ5868.1	5.84162192	PMS1_HUMAN	1E-97	0

*Number in this column shows how many organs these TFs are differentially expressed in during aestivation.

Table S29 KEGG enrichment analysis of AM7.

MapID	MapTitle	Pvalue	AdjustedPv	DEGs Ratio
map04330	Notch signaling pathway	8.22E-19	2.32E-16	0.94
map04320	Dorso-ventral axis formation	5.55E-18	7.83E-16	0.90
map04919	Thyroid hormone signaling pathway	2.16E-16	2.03E-14	0.86
map04670	Leukocyte transendothelial migration	6.48E-11	3.66E-09	0.82
map04360	Axon guidance	2.42E-09	1.14E-07	0.80
map04141	Protein processing in endoplasmic reticulum	1.41E-07	4.43E-06	0.72
map04062	Chemokine signaling pathway	2.34E-06	5.49E-05	0.73
map04630	Jak-STAT signaling pathway	3.06E-06	6.65E-05	0.71
map04610	Complement and coagulation cascades	5.45E-05	0.001098	0.73
map04520	Adherens junction	9.25E-05	0.001631	0.84
map04510	Focal adhesion	0.000119	0.001972	0.85
map04151	PI3K-Akt signaling pathway	0.000168	0.002355	0.80
map04970	Salivary secretion	0.000175	0.002355	0.89
map04810	Regulation of actin cytoskeleton	0.000202	0.002586	0.75
map04012	ErbB signaling pathway	0.000253	0.002857	0.63
map00534	Glycosaminoglycan biosynthesis - heparan sulfate	0.000496	0.005176	0.55
map05202	Transcriptional misregulation in cancer	0.000671	0.006524	0.68
map04514	Cell adhesion molecules (CAMs)	0.000978	0.008622	0.78
map04722	Neurotrophin signaling pathway	0.001021	0.008725	0.72
map05200	Pathways in cancer	0.001126	0.009338	0.69
map04015	Rap1 signaling pathway	0.001235	0.009953	0.78
map04620	Toll-like receptor signaling pathway	0.002217	0.014539	0.61
map00513	High-mannose type N-glycan biosynthesis	0.0027	0.016919	0.59
map04668	TNF signaling pathway	0.00282	0.016921	0.73
map04380	Osteoclast differentiation	0.00282	0.016921	0.64
map04740	Olfactory transduction	0.003207	0.018839	0.87
map04370	VEGF signaling pathway	0.004025	0.02174	0.81
map04918	Thyroid hormone synthesis	0.004086	0.02174	0.71
map04014	Ras signaling pathway	0.004734	0.023838	0.76
map04662	B cell receptor signaling pathway	0.007345	0.034522	0.86
map04917	Prolactin signaling pathway	0.007345	0.034522	0.64
map04664	Fc epsilon RI signaling pathway	0.008975	0.040175	0.77
map05215	Prostate cancer	0.011432	0.048844	0.63

Table S30 Summary of hyper-methylated genes (HMGs) in intestine during sea cucumber aestivation.

See the separate Excel file.

Table S31 Summary of RNA-seq data from various stages of intestine regeneration.

	Total reads	Total mapped reads	Mapped percentage
0d-1	25,690,760	20,621,818	80.27%
0d-2	25,659,527	20,698,623	80.67%
0d-3	24,157,739	19,853,535	82.19%
10d-1	31,413,872	25,669,338	81.71%
10d-2	22,330,288	15,720,336	70.39%
10d-3	21,256,361	17,385,910	81.79%
20d-1	21,851,466	18,565,031	84.96%
20d-2	36,231,981	30,457,686	84.06%

Table S32 Summary of DEGs during intestine regeneration.

See the separate Excel file.

Table S33 GO enrichment analysis of intestine regeneration-related DEGs.

GO_ID	GO_Term	GO_Class	Pvalue	AdjustedPv
GO:0016043	cellular component organization	BP	1.73E-08	9.47E-07
GO:0033036	macromolecule localization	BP	8.51E-07	3.26E-05
GO:0006996	organelle organization	BP	1.26E-06	4.40E-05
GO:0045184	establishment of protein localization	BP	4.67E-05	0.001165
GO:0044767	single-organism developmental process	BP	4.87E-05	0.001165
GO:0016049	cell growth	BP	0.0001	0.002136
GO:0065009	regulation of molecular function	BP	0.000159	0.003048
GO:0048856	anatomical structure development	BP	0.000325	0.005934
GO:0044707	single-multicellular organism process	BP	0.0005	0.008711
GO:0042445	hormone metabolic process	BP	0.000966	0.014935
GO:0051129	negative regulation of cellular component organization	BP	0.00131	0.018576
GO:0019882	antigen processing and presentation	BP	0.002087	0.025787
GO:0051130	positive regulation of cellular component organization	BP	0.002758	0.03232
GO:0035265	organ growth	BP	0.002785	0.03232
GO:0051128	regulation of cellular component organization	BP	0.003099	0.034914
GO:0009653	anatomical structure morphogenesis	BP	0.003301	0.036117
GO:0051641	cellular localization	BP	0.003545	0.037719
GO:0009605	response to external stimulus	BP	0.004421	0.043301
GO:0050794	regulation of cellular process	BP	0.004522	0.043301
GO:0050789	regulation of biological process	BP	0.004522	0.043301
GO:0031982	vesicle	CC	1.71E-11	6.54E-09
GO:0070161	anchoring junction	CC	6.66E-11	1.28E-08
GO:0043230	extracellular organelle	CC	1.08E-09	1.30E-07
GO:0012505	endomembrane system	CC	1.35E-09	1.30E-07
GO:0030055	cell-substrate junction	CC	2.85E-09	2.19E-07
GO:0044421	extracellular region part	CC	9.60E-08	4.60E-06
GO:0044449	contractile fiber part	CC	5.24E-07	2.23E-05
GO:0044424	intracellular part	CC	3.63E-06	0.000116
GO:0005911	cell-cell junction	CC	3.93E-06	0.000116
GO:0005886	plasma membrane	CC	2.02E-05	0.000553
GO:0045177	apical part of cell	CC	6.40E-05	0.001442
GO:0044464	cell part	CC	0.000115	0.002326

GO:0043227	membrane-bounded organelle	CC	0.000798	0.013288
GO:0030904	retromer complex	CC	0.000975	0.014935
GO:0005604	basement membrane	CC	0.001659	0.021911
GO:0043229	intracellular organelle	CC	0.001984	0.025332
GO:0005515	protein binding	MF	6.26E-09	4.00E-07
GO:0008289	lipid binding	MF	0.001131	0.016663
GO:0016787	hydrolase activity	MF	0.00141	0.019281

Table S34 Module enrichment analysis of intestine regeneration-related genes.

Module color	Module size	Number of DEGs in the module	Ratio	Hyper	FDR
RM7	3562	2729	0.766143	0	0
RM8	2427	1568	0.646065	2.84E-116	1.28E-115
RM6	1911	703	0.36787	1	1
RM4	395	138	0.349367	0.999804	1
RM9	1328	422	0.317771	1	1
RM10	671	192	0.28614	1	1
RM5	1444	382	0.264543	1	1
RM3	694	125	0.180115	1	1
RM2	1175	168	0.142979	1	1
RM1	1377	84	0.061002	1	1

Table S35 KEGG enrichment analysis of the intestine regeneration-related module RM7.

ID	MapTitle	Pvalue	AdjustedPv
map04510	Focal adhesion	9.95E-23	2.91E-20
map04810	Regulation of actin cytoskeleton	1.49E-17	2.18E-15
map04512	ECM-receptor interaction	8.65E-14	6.31E-12
map04670	Leukocyte transendothelial migration	1.36E-13	7.93E-12
map05205	Proteoglycans in cancer	1.15E-11	5.60E-10
map04151	PI3K-Akt signaling pathway	2.83E-11	1.18E-09
map05222	Small cell lung cancer	6.74E-10	2.46E-08
map04530	Tight junction	1.10E-09	3.57E-08
map05200	Pathways in cancer	3.51E-09	9.56E-08
map04062	Chemokine signaling pathway	3.60E-09	9.56E-08
map04722	Neurotrophin signaling pathway	1.77E-07	3.45E-06
map04012	ErbB signaling pathway	3.35E-07	6.11E-06
map04520	Adherens junction	5.24E-07	8.93E-06
map04391	Hippo signaling pathway - fly	5.51E-07	8.93E-06
map05212	Pancreatic cancer	6.12E-07	9.41E-06
map05210	Colorectal cancer	6.49E-07	9.47E-06
map04360	Axon guidance	1.92E-06	2.43E-05
map05203	Viral carcinogenesis	3.83E-06	4.48E-05
map04068	FoxO signaling pathway	5.39E-06	5.83E-05
map04152	AMPK signaling pathway	5.82E-06	6.07E-05
map04015	Rap1 signaling pathway	9.43E-06	9.49E-05
map05220	Chronic myeloid leukemia	1.38E-05	0.000134
map05410	Hypertrophic cardiomyopathy (HCM)	2.02E-05	0.000184
map04310	Wnt signaling pathway	2.50E-05	0.000207
map04611	Platelet activation	2.56E-05	0.000207
map05414	Dilated cardiomyopathy	3.16E-05	0.000249
map04071	Sphingolipid signaling pathway	3.29E-05	0.000253
map04370	VEGF signaling pathway	5.14E-05	0.000375
map04660	T cell receptor signaling pathway	6.15E-05	0.000438
map05214	Glioma	7.10E-05	0.000494
map04150	mTOR signaling pathway	0.000129	0.000875
map04014	Ras signaling pathway	0.00016	0.001064
map05412	Arrhythmogenic right ventricular cardiomyopathy (ARVC)	0.000175	0.001136
map04261	Adrenergic signaling in cardiomyocytes	0.000208	0.001322
map04930	Type II diabetes mellitus	0.000217	0.001347
map04917	Prolactin signaling pathway	0.000223	0.001359
map05215	Prostate cancer	0.000283	0.001656
map04380	Osteoclast differentiation	0.000289	0.001656
map04960	Aldosterone-regulated sodium reabsorption	0.000305	0.001695

map04650	Natural killer cell mediated cytotoxicity	0.000308	0.001695
map04978	Mineral absorption	0.000392	0.002081
map05213	Endometrial cancer	0.000499	0.002601
map04662	B cell receptor signaling pathway	0.000898	0.004445
map04910	Insulin signaling pathway	0.001022	0.004974
map05231	Choline metabolism in cancer	0.001315	0.006296
map05166	HTLV-I infection	0.00144	0.006781
map05223	Non-small cell lung cancer	0.001903	0.008822
map04620	Toll-like receptor signaling pathway	0.002086	0.009371
map00030	Pentose phosphate pathway	0.002153	0.009526
map04514	Cell adhesion molecules (CAMs)	0.002222	0.009683
map05218	Melanoma	0.002868	0.012136
map04390	Hippo signaling pathway	0.003172	0.012862
map04142	Lysosome	0.005047	0.019915
map04668	TNF signaling pathway	0.005504	0.020872
map04666	Fc gamma R-mediated phagocytosis	0.005504	0.020872
map04070	Phosphatidylinositol signaling system	0.007981	0.029879
map05211	Renal cell carcinoma	0.008587	0.031341
map05221	Acute myeloid leukemia	0.008985	0.032389
map04022	cGMP-PKG signaling pathway	0.010003	0.035192
map04120	Ubiquitin mediated proteolysis	0.011336	0.039405
map05014	Amyotrophic lateral sclerosis (ALS)	0.011757	0.04039
map04630	Jak-STAT signaling pathway	0.012562	0.042651
map00562	Inositol phosphate metabolism	0.015026	0.049858

Table S36 KEGG enrichment analysis of in the intestine regeneration-related module RM8.

ID	MapTitle	Pvalue	AdjustedPv
map04146	Peroxisome	1.33E-12	4.25E-10
map00071	Fatty acid degradation	3.24E-08	5.17E-06
map01230	Biosynthesis of amino acids	9.23E-08	9.82E-06
map01212	Fatty acid metabolism	4.47E-07	3.57E-05
map03320	PPAR signaling pathway	1.02E-06	5.42E-05
map00360	Phenylalanine metabolism	7.39E-06	0.000262
map00020	Citrate cycle (TCA cycle)	1.67E-05	0.000532
map01210	2-Oxocarboxylic acid metabolism	3.32E-05	0.000963
map00830	Retinol metabolism	6.14E-05	0.001576
map00052	Galactose metabolism	6.98E-05	0.001576
map01040	Biosynthesis of unsaturated fatty acids	7.42E-05	0.001576
map01200	Carbon metabolism	7.49E-05	0.001576
map00592	alpha-Linolenic acid metabolism	8.01E-05	0.001576
map00380	Tryptophan metabolism	0.000418	0.006662
map00960	Tropane, piperidine and pyridine alkaloid biosynthesis	0.000469	0.007119
map00270	Cysteine and methionine metabolism	0.000639	0.009268
map05120	Epithelial cell signaling in Helicobacter pylori infection	0.000978	0.012995
map00600	Sphingolipid metabolism	0.001218	0.014945
map00500	Starch and sucrose metabolism	0.001318	0.015574
map04614	Renin-angiotensin system	0.00143	0.015985
map00130	Ubiquinone and other terpenoid-quinone biosynthesis	0.001521	0.016024
map00401	Novobiocin biosynthesis	0.001752	0.016486
map04721	Synaptic vesicle cycle	0.001809	0.016486
map00970	Aminoacyl-tRNA biosynthesis	0.001809	0.016486
map00250	Alanine, aspartate and glutamate metabolism	0.002368	0.020417
map00950	Isoquinoline alkaloid biosynthesis	0.002767	0.023229
map00280	Valine, leucine and isoleucine degradation	0.003385	0.026763
map00400	Phenylalanine, tyrosine and tryptophan biosynthesis	0.003457	0.026763
map00330	Arginine and proline metabolism	0.003476	0.026763
map00920	Sulfur metabolism	0.005238	0.037133
map00591	Linoleic acid metabolism	0.007294	0.049504

Table S37 The accession numbers and IDs of the genes displayed in Figure 5d.

See the separate Excel file.

References

1. Dolezel, J., Bartos, J., Voglmayr, H., Greilhuber, J. Nuclear DNA content and genome size of trout and human. *Cytom. Part A.* **51**, 127-128 (2013).
2. Saitou, N. & Nei, M. The neighbor-joining method: a new method for reconstructing phylogenetic trees. *Mol. Biol. Evol.* **4**, 406-425 (1987).
3. Nei, M. &, Kumar, S. Molecular evolution and phylogenetics. *Heredity* **86**, 385-385 (2001).
4. Felsenstein, J. Confidence limits on phylogenies: an approach using the bootstrap. *Evolution* **39**: 783-791 (1985).
5. Thompson, J. D., Gibson, T. J. & Higgins, D.G. Multiple sequence alignment using ClustalW and ClustalX. *Curr. Protoc. Bioinformatics* **Ch. 2**: Unit 2.2-3 (2002)
6. Zhang, X., *et al*. The sea cucumber genome provides insights into morphological evolution and visceral regeneration. *PLoS Biol.* **15**, e2003790 (2017).
7. Jo, J., *et al*. Draft genome of the sea cucumber *Apostichopus japonicus* and genetic polymorphism among color variants. *Gigascience* **6**, 1-6 (2017).
